# Enteropathogenic *Escherichia coli*-mediated fast and coordinated Ca^²+^ responses regulate NF-κB activation

**DOI:** 10.7554/eLife.108953

**Published:** 2026-07-22

**Authors:** Fangrui Guo, Roberto Ornelas Guevara, Linda Oussaedine, Geneviève Dupont, Laurent Combettes, Guy Tran Van Nhieu

**Affiliations:** 1 Team 'Ca²⁺ signaling and Microbial Infections,' Institute for Integrative Biology of the Cell (I2BC), CEA, CNRS UMR9198, Université Paris-Saclay Gif-sur-Yvette France; 2 Institut National de la Santé et de la Recherche Médicale Gif-sur-Yvette France; 3 Unit of Theoretical Chronobiology, Université Libre de Bruxelles Brussels Belgium; Tufts University School of Medicine United States; Harvard T.H. Chan School of Public Health United States

**Keywords:** local Ca²⁺ responses, modeling of coordinated Ca2+ responses, inflammatory signaling pathways, *E. coli*, Human

## Abstract

Enteropathogenic *Escherichia coli* (EPEC) is a major bacterial enteropathogen causing infectious diarrhea among children in developing countries. Here, we found that EPEC induced isolated Ca^2+^ responses in epithelial cells, triggered by extracellular ATP (eATP). These responses were dependent on type III secretion (T3S) and down-regulated by the bacterial secreted protease EspC, consistent with eATP released by the T3S translocon pore-forming activity in host membranes. By performing high-speed Ca^2+^ imaging, we uncovered that at the onset of infection, low eATP levels triggered Ca^2+^-responses involving the whole cell but showing small amplitude and fast kinetics usually associated with local Ca^2+^ responses. The findings, supported by theoretical modeling, evoke a conceptual shift whereby low amounts of inositol 1, 4, 5-trisphosphate (IP_3_) induced by low eATP levels and subsequent moderate Ca^2+^ release enable the fast coordination of IP_3_ receptor cluster activation throughout the cell. Importantly, these yet undescribed coordinated fast responses occurred over prolonged time periods and defined a cell state with dampened activation of the pro-inflammatory transcriptional activator NF-kB associated with a decrease in its Ca^2+^-dependent O-linked β-*N*-acetylglucosamine modification.

## Introduction

EPEC are diarrheagenic *E. coli* strains that cause significant morbidity and mortality in children under two years of age. While infection rates have significantly declined in industrialized nations, EPEC remains a major public health concern in low-income countries (Lozer et al., 2013). EPEC forms attaching and effacing (A/E) lesions on intestinal epithelial cells and lacks the ability to produce Shiga toxins or heat-labile (LT) and heat-stable (ST) enterotoxins (Croxen et al., 2013; Gomes et al., 2016; Hazen et al., 2016). The ability of EPEC to form A/E lesions is determined by the locus of Enterocyte Effacement (LEE), a large genomic pathogenicity island that encodes the essential genetic elements required for this process (Pearson et al., 2016). The LEE region of EPEC (E2348/69) encodes components of the type III secretion system (T3SS), a molecular apparatus that translocates at least 25 bacterial effector proteins into the host cell. The EPEC type III secretion system (T3SS) consists of a basal body and a needle-like structure, resembling those found in *Salmonella* and *Shigella*, but with a distinct sheath-like extension at the needle tip, primarily composed of EspA, and about ten times longer than the T3SS needles from other bacterial species. This EspA filament acts as a molecular bridge, extending from the bacterium to the host cell membrane, allowing insertion of the EspB and EspD translocon components into the host cell membrane, enabling type III effectors injection in the cell cytosol (Creasey et al., 2003; Monjarás Feria et al., 2012; Sal-Man et al., 2012). Osmoprotection assays suggest that the translocon forms a pore with an internal diameter ranging between 3–5 nm, allowing the passage of unfolded effector proteins into the host cell (Chatterjee et al., 2015). However, the T3SS translocon shows weak pore-forming activity during EPEC infection of epithelial cells, presumably because it forms a sealed conduct between the T3SS and host cell membranes (Guignot and Tran Van Nhieu, 2016). EspC, a secreted serine protease from the autotransporter family, targets EspA and EspD and down-regulates pore formation activity associated with cytotoxicity (Guignot et al., 2015). EPEC T3SS effector proteins translocated into host cells lead are responsible for attaching and effacing (A/E) lesions and intimate bacterial adhesion to the host cells associated with the formation of an actin-rich pedestal formation (Chen and Frankel, 2005).

The detection of pathogenic bacteria by intestinal epithelial cells plays an important role in initiating pro-inflammatory responses. Recognition of bacterial surface components by pattern recognition receptors triggers pro-inflammatory signaling pathways involving the transcriptional activator NF-κB and the production of cytokines, such as interleukin-8 and tumor necrosis factor-α (TNF-α) (Edwards et al., 2011). However, EPEC suppresses these signaling pathways early in infection through the coordinated action of several T3SS effector proteins. Among the first translocated T3SS effectors, Tir interacts with TNF-α receptor-associated factors (TRAF2 and TRAF6), recruiting the tyrosine phosphatases SHP-1 and SHP-2 (Mills et al., 2008; Ruchaud-Sparagano et al., 2011; Yan et al., 2013). Several non-LEE T3SS effectors, including NleE, NleB, NleH1, NleH2, NleC, and NleD, further contribute to inhibiting NF-κB and MAPK signaling. NleE and NleB stabilize the interaction between NFκB and its inhibitory subunit IκB, preventing its degradation, thereby keeping NFκB in an inactive state. The ability of NleE to inhibit NFκB signaling depends on its S-adenosyl-L-methionine (SAM)-dependent methyltransferase activity (Zhang et al., 2012). NleE-mediated methylation of TAB2/3 prevents IKK activation (Zhang et al., 2012). NleB selectively blocks NFκB activation through GlcNAcylation of the TNF-α receptor (TNFR) adaptor protein and TNFR1-associated death domain (TRADD) (Li et al., 2013; Pearson et al., 2013). The T3SS effectors NleH1, NleH2, and NleC also interfere with NFκB nuclear translocation (Gao et al., 2009). NleC specifically cleaves P65 RelA (Giogha et al., 2015; Ruchaud-Sparagano et al., 2011; Yen et al., 2010). Interestingly, NleF has been implicated in the activation of NF-κB, underscoring the complexity of the regulation of inflammation during bacterial infection and suggesting the timing of various T3SS effectors’ activity (Pallett et al., 2014).

A similar complexity applies to T3SS effectors regulating cell death and survival pathways during EPEC infection of epithelial cells. Tir was found to elicit a rapid Ca²^+^ influx across the host cell membrane, through the activation of a host plasma membrane Ca²^+^ channel, the mechanosensitive transient receptor potential vanilloid 2 (TRPV2) leading to pyroptosis (Zhong et al., 2022). However, the NleA effector blocks the delivery of TRPV2 channels to the cell surface, thereby dampening Tir-induced Ca²^+^ influx. The effector NleF also directly binds caspase-4 to inhibit its activity (Zhong et al., 2020). The extrinsic apoptotic pathway is triggered by EPEC pili (Abul-Milh et al., 2001). However, the NleD and NleB effectors inhibit this pathway by cleaving JNK and GlcNAcylating the death domain adaptor proteins TRADD and FADD, respectively (Baruch et al., 2011; Pearson et al., 2013). While EspC prevents cytotoxicity linked to pore-formation by the T3SS translocon during the early EPEC infection phases, it was shown to promote intrinsic apoptosis through an increase in intracellular Ca^2+^ and calpain activation (Serapio-Palacios and Navarro-Garcia, 2016).

Central to inflammation and cell death/survival pathways induced by EPEC, bacterial-induced Ca^2+^ signals have been a matter of debate. EPEC infection is known to perturb host Ca²^+^ signaling, but the source and sequence of Ca²^+^ signals during infection remain controversial. EPEC was shown to induce Ca²^+^ influx associated with a loss of mitochondrial membranes permeability leading to cell death (Zhong et al., 2020; Ramachandran et al., 2020; Zhong et al., 2022), but was also reported to trigger IP_3_-mediated Ca²^+^ release possibly involved in bacterial-induced cytoskeletal rearrangements (Baldwin et al., 1991; Baldwin et al., 1993; Foubister et al., 1994; Bain et al., 1998).

Here, we investigated the characteristics and implications of EPEC-induced Ca²^+^ responses in epithelial cells. We characterized yet undescribed Ca²^+^ signals induced by EPEC and low ATP levels, presenting the fast dynamics and small amplitude of local Ca²^+^ responses but involving large cell area. We found that these responses likely result from the coordination of elementary responses via rapid Ca^2+^-induced Ca²^+^ release over large cell area, challenging generally admitted concepts on Ca²^+^ diffusion. Importantly, we show that these newly described responses have functional implications by dampening the cell ability to respond to inflammatory signals.

## Results

### EPEC induces Ca^²+^ responses that depend on type III secretion-mediated eATP release

Despite their critical role in cellular processes key to bacterial infection, EPEC-induced Ca²^+^ responses remain to be characterized. We, therefore, set up to perform a detailed single-cell imaging of Ca²^+^ responses elicited by cells infected by EPEC.

**Figure 1.**
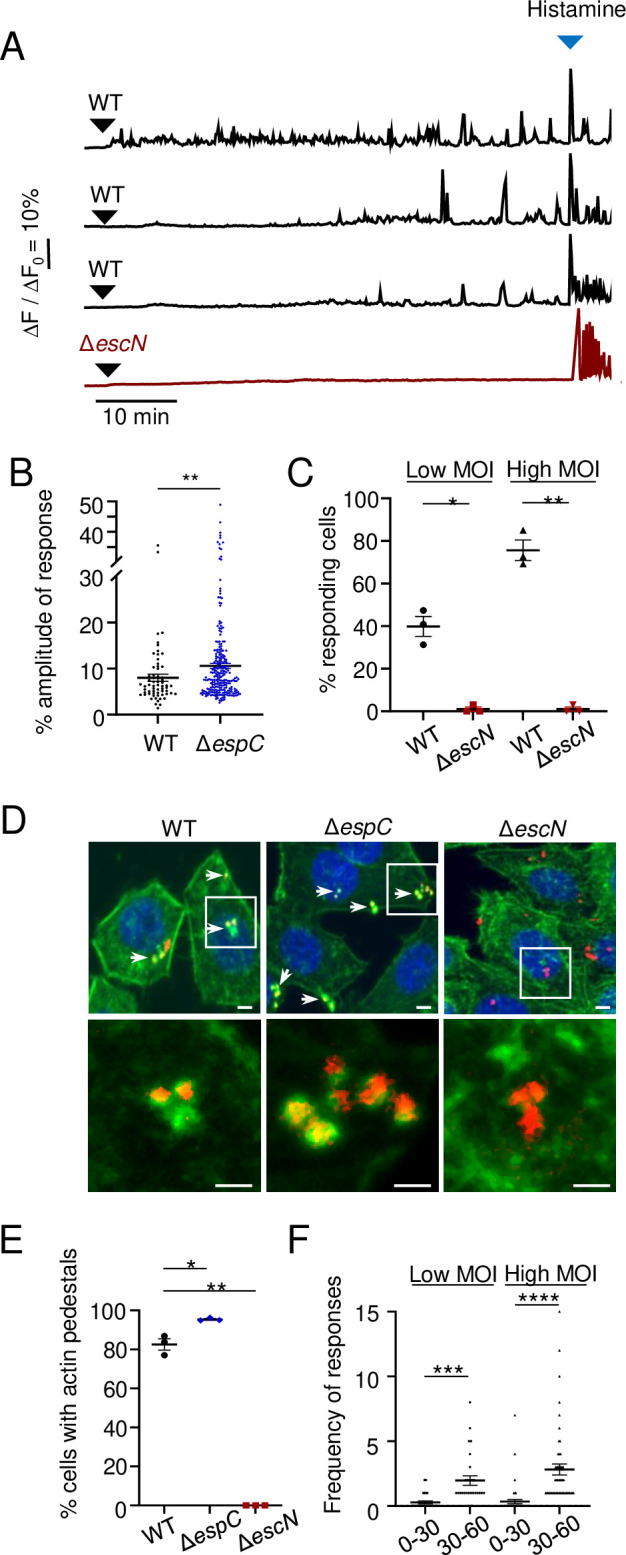
Enteropathogenic *Escherichia coli* (EPEC) induces isolated Ca^2+^ responses of limited amplitude in epithelial cells. HeLa cells were loaded with the fluorescent indicator Cal-520, challenged with the indicated bacteria, and subjected to live-cell Ca^2+^ imaging at a frequency of one acquisition every 10 s (**A–C, F**) or fixed and processed for fluorescence microscopy analysis (**D–E**) (Materials and methods). (**A**) Representative traces of Ca^2+^ variations in single cells. The black arrowheads indicate the time of bacterial challenge. The blue arrowheads indicate stimulation with 3 µM histamine. (**B**) Response amplitude expressed as a percent of the maximal histamine response amplitude (N=3, n>63). (**C**) Percent of cells exhibiting Ca^2+^ responses (N=3, cells >66). (**D, E**) Cells challenged with red fluorescent protein (RFP)-expressing bacteria for 1 hr. (**D**) Representative confocal micrographs. Staining with DAPI (blue), phalloidin-Alexa 488 (green). The lower panels show a higher magnification of the insets in the top panels. Scale bar = 10 µm. (**E**) Percentage of bacteria-associated actin-rich pedestals (N=3, cells >273). (**F**) Average number of responses per cell during the first 30 min (0–30) and last 30 min (30-60) of bacterial challenge. Low MOI: 10 bacteria/cell. High multiplicity of infection (MOI): 50 bacteria/cell. Bar: mean. (N=3, cells >63). Mann–Whitney test. *p<0.05; **p<0.01; ***p<0.001; ****p<0.0001. Figure 1—source data 1.All the source data for the graphs (B,C,E,F) in Figure 1.

As shown in Figure 1, EPEC induced Ca²^+^ transients often corresponding to a single peak of varying amplitude detected over several minutes, corresponding to 6.2±0.8% (mean ± SEM) of the maximal histamine response (Figure 1A and B). These Ca²^+^ responses were dependent on a functional T3SS, since they were not observed for the T3SS-deficient *escN* mutant (Figure 1A and C). Only 40±4.7% (mean ± SEM) of cells, however, elicited responses when challenged with wild-type EPEC at a low multiplicity of infection (MOI) of 20 bacteria per cell, a value that raised to 76 ± 4.8% (mean ± SEM) when using a high MOI of 80 bacteria per cell (Figure 1C). In contrast, even at the low MOI, more than 83% of cells showed actin pedestals, indicating that Ca²^+^ responses were elicited only in a fraction of cells targeted by EPEC-type T3SS (Figure 1D and E). The frequency of Ca²^+^ responses per cell increased over the incubation time with an average frequency of responses per cell raising by 6.9–8.3-fold from the first to the last 30 min of EPEC challenge (Figure 1F). This increased frequency suggested the accumulation of an agonist in the extracellular medium during the course of the infection triggering IP_3_-mediated Ca²^+^ release. When pooling all single-cell responses, we could observe a steady increase in the average cytosolic Ca^2+^ concentration of the cell population, as previously reported (Ramachandran et al., 2020; Figure 1—figure supplement 1B). However, when performing single-cell imaging, we did not observe an increase in cytosolic Ca²^+^ basal levels even at high MOI after 2 hr incubation with wild-type bacteria (Figure 1—figure supplement 1).

**Figure 1—figure supplement 1.**
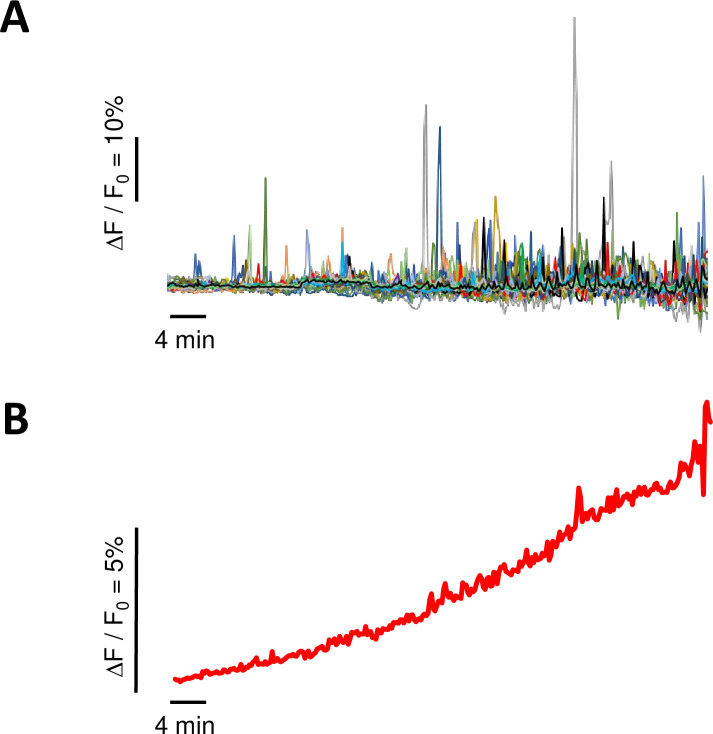
Enteropathogenic *Escherichia coli* (EPEC) induces Ca^2+^ responses with a frequency increasing during the time of infection. Ca^2+^ imaging was performed on HeLa cells loaded with the Ca^2+^ fluorescent indicator Cal-520 and challenged with EPEC at a multiplicity of infection (MOI) of 100. (**A**) Traces of Ca^2+^ variations in single cells. (**B**) Trace corresponding to the average of traces shown in B (N=2, cells = 43). Note that cells do not show an increase in basal Ca^2+^ levels that could be mistakenly interpreted from the averaging of Ca^2+^ responses over the cell population.

Together, these results suggest that EPEC induces isolated Ca²^+^ responses that depend on the T3SS for the release of limiting amounts of a Ca²^+^ agonist.

### EPEC-mediated Ca²^+^ responses depend on ATP released in the extracellular medium via the T3SS translocon

In previous works, we showed that the EPEC T3SS translocon forms pores in host cell plasma membranes that were down-regulated by the bacterial secreted serine protease EspC (Guignot and Tran Van Nhieu, 2016). We posit that low amounts of ATP released in the extracellular medium by the T3SS translocon were responsible for the isolated Ca²^+^ responses of reduced amplitude elicited by EPEC. According to this view, by removing T3SS translocons from host cell membranes, EspC would down-regulate EPEC-mediated Ca^2+^ signaling explaining the low ratio of Ca^2+^ responding cells relative to cells forming actin pedestals.

**Figure 2.**
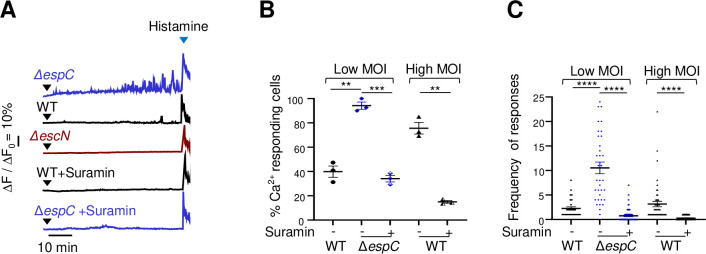
Enteropathogenic *Escherichia coli* (EPEC)-induced Ca^2+^ responses are elicited by ATP released by the type III secretion system (T3SS) translocon. HeLa cells were loaded with the fluorescent indicator Cal-520 or with 200 μM suramin for 30 min, challenged with the indicated bacteria, and subjected to live-cell Ca^2+^ imaging for a 60 min-duration at a frequency of one acquisition every 10 s. (**A**) Representative traces of Ca^2+^ variations in single cells. The black arrowheads indicate the time of bacterial challenge. The blue arrowheads indicate stimulation with 3 µM histamine. (**B**) Percent of cells exhibiting Ca^2+^ responses (N=3, cells >70). (**C**) Average number of responses per cell. Low multiplicity of infection (MOI): 10 bacteria/cell. High MOI: 50 bacteria/cell. Bar: mean. (N=3, cells >30). Mann–Whitney test. **p<0.01; ***p<0.001; ****p<0.0001. Figure 2—source data 1.All the source data for the graphs (B & C) in Figure 2.

Consistent with this and as shown in Figure 2A–C, an *espC* mutant induced more Ca²^+^ responses than wild-type EPEC, with 94±3% responding cells and a frequency of 10.5±1.2 responses per cell over the 60 min analysis, compared 40±4.7% responding cells and less than 2 responses per cell for the *espC* mutant and wild-type EPEC, respectively. Also, the average amplitude of Ca²^+^ responses induced by the *espC* mutant was higher than that of wild-type EPEC, suggesting more eATP release (Figure 1B). Accordingly, cell treatment with the purinergic receptors’ antagonists Suramin and PPADS, as well as with hexokinase to deplete eATP, inhibited Ca²^+^ responses induced by the wild-type and *espC* mutant strains (Figure 2B, C, Figure 2—figure supplement 1A-C). Treatment with the PLC inhibitor U73122, but not its inactive analog U73343, also resulted in inhibition of EPEC-mediated Ca²^+^ responses (Figure 2—figure supplement 1A, B). As expected for ATP-mediated Ca^2+^ release, sample treatment with EGTA, a cell impermeant chelator of extracellular Ca^2+^ did not decrease the percent of Ca^2+^ responding cells triggered by wild-type EPEC or the *espC* mutant (Figure 2—figure supplement 1C). The frequency of responses per cell was also not inhibited and even appeared to increase upon EGTA treatment in cells challenged with wild-type EPEC and the *espC* mutant (Figure 2—figure supplement 1D, E). In control experiments, Suramin treatment did not affect actin pedestal structures induced by these strains (Figure 2—figure supplement 2).

**Figure 2—figure supplement 1.**
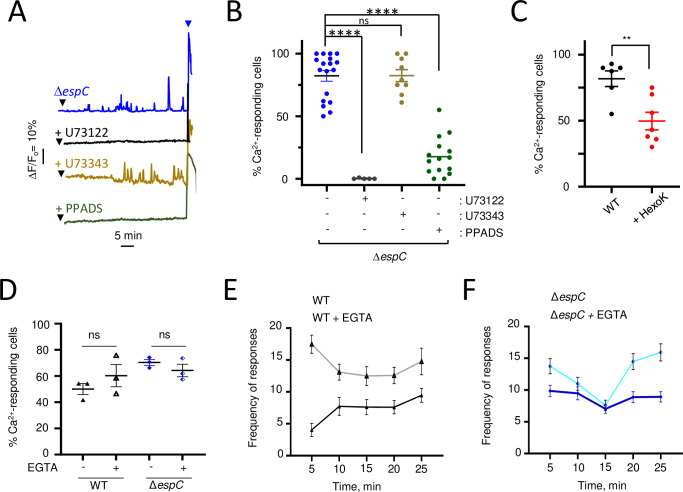
Enteropathogenic *Escherichia coli* (EPEC)-induced Ca^2+^ responses do not depend on Ca^2+^ influx but on Ca^2+^ release. HeLa cells were loaded with the fluorescent indicator Cal-520, challenged with a high multiplicity of infection (MOI) of EPEC wild-type (WT) or a low MOI of the *ΔespC* mutant and subjected to live-cell Ca^2+^ imaging at a frequency of one acquisition every 10 s (**A-C**). (**A**) Representative traces of Ca^2+^ variations in single cells. The black arrowheads indicate the time of bacterial challenge. The blue arrowheads indicate stimulation with 3 µM histamine. (**B**) Percent of cells exhibiting Ca^2+^ responses. Cells challenged with: *ΔespC* (N=4, cells >400); +U73122: *ΔespC* in the presence of 10 μM U73122 (N=4, cells >160);+U73343: *ΔespC* in the presence of 10 μM U73343 (N=2, cells = 230); +PPADS: *ΔespC* in the presence of 20 μM PPADS (N=4, cells >400). (**C**) Cells challenged with wild-type EPEC in the absence or presence of +hexokinase (200 units/ml) and 5 mM glucose (+HexoK, N=3, cells >120). (**D–F**) +EGTA: cells treated with 4 mM EGTA. (N=3, cells >30). (**D**) Percent of cells showing Ca^2+^ responses. (**E**, **F**) Frequency of Ca^2+^ responses per cell. Mann–Whitney test. ns: not significant. **p<0.01; ****p<0.0001. Figure 2—figure supplement 1—source data 1.All the source data for the graphs (B,C,D,E,F) in Figure 2—figure supplement 1.

**Figure 2—figure supplement 2.**
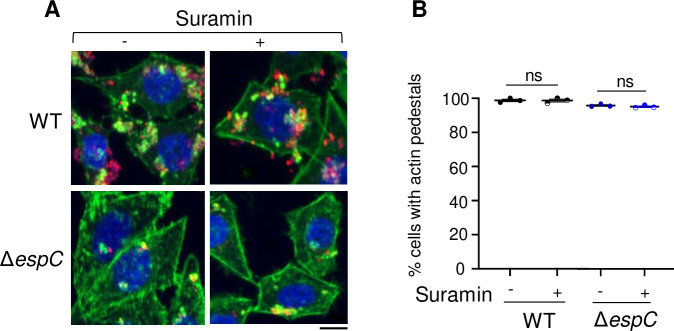
Suramin does not affect enteropathogenic *Escherichia coli* (EPEC)-induced actin pedestals. HeLa cells were challenged with the indicated red fluorescent protein (RFP)-expressing bacterial strains in the presence or absence of 100 μM suramin. Samples were fixed and processed for fluorescence staining. (**A**) Representative micrographs. Blue: DAPI; green: Phalloidin-Alexa488; red: bacteria. Scale bar = 10 µm. (**B**) Percent of cells exhibiting actin-rich pedestals. High multiplicity of infection (MOI): 50 bacteria/cell. Low MOI *ΔespC*: 10 bacteria/cell. (N=3, n>150). Mann–Whitney test. ns: not significant. Figure 2—figure supplement 2—source data 1.Source data for the graph B in Figure 2—figure supplement 2.

Together, these results suggest that Ca²^+^ responses induced by EPEC are mediated by ATP released in the extracellular medium via pores formed by the T3SS translocon and are down-regulated by EspC.

### EPEC induces coordinated Ca²^+^ responses from single IP_3_R clusters

We previously showed that *Shigella* induced local Ca^2+^ responses dependent on the T3SS and Ca^2+^ release (Tran Van Nhieu et al., 2013), suggesting that insertion of the Type III translocon was responsible for bacterial-induced local Ca^2+^ signals. We, therefore, set up to investigate whether EPEC could also trigger T3SS-dependent local Ca^2+^ responses.

**Figure 3.**
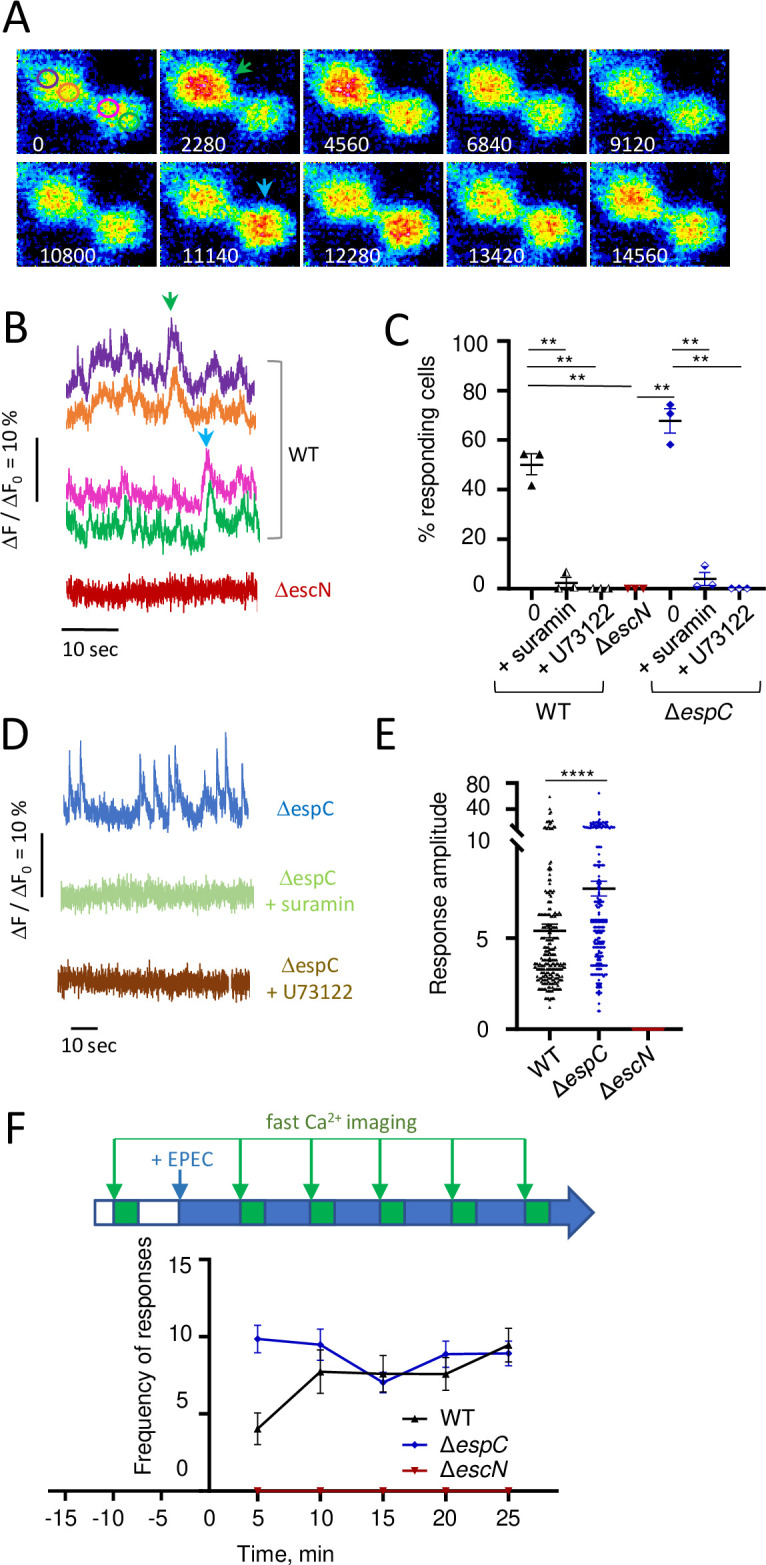
Enteropathogenic *Escherichia coli* (EPEC) induces rapid and coordinated elementary Ca^2+^ responses. HeLa cells were loaded with the fluorescent indicator Cal-520, challenged with the indicated bacteria, and subjected to high-speed Ca^2+^ imaging at a frequency of one acquisition every 57 ms for a duration of 110 s. (**A**) Representative time-series of pseudocolored fluorescent micrographs of cells challenged with wild-type EPEC. The numbers indicate the elapsed time in ms from an arbitrarily determined origin. Scale bar = 10 µm. (**B, D**) Traces of Ca^2+^ variations in 2 subcellular regions of the same cell. (**B**) WT: Traces corresponding to the regions depicted in the Figure 0 of panel **A**. The arrowheads point to the Ca^2+^ responses shown in Panel A with the corresponding color. (**C**) Percent of cells exhibiting Ca^2+^ responses (N>3, cells >134). +Suramin: treatment with 200 μM Suramin. +U73122: treatment with 10 μM U73122. (**E**) Response amplitude expressed as a percent of the maximal response amplitude induced by treatment with 3 μM histamine (N=3, cells >166). (**F**) Average number of responses per cell. (**C, E**) Bar: mean. Mann–Whitney test. **p<0.01; ****p<0.0001. (**F**) High-speed Ca^2+^ imaging was performed every 5 min for 110 s following infection with the indicated bacterial strain as depicted the scheme. The average number of responses per cell is indicated (N>3, cells >29). Figure 3—source data 1.All the source data for the graphs (C, E) in Figure 3.

To explore this, we performed rapid Ca^2+^ imaging at a frequency of 57 ms acquisition per frame to sample elementary Ca^2+^ release events. As shown in Figure 3, by performing high-speed Ca^2+^ imaging, we detected fast Ca^2+^ increases associated with cell challenge with EPEC. These fast Ca^2+^ responses did not correspond to other responses previously reported since they involved the whole cell or a large cell area but showed a small amplitude and fast dynamics usually associated with local Ca^2+^ responses (Figure 3B). As observed for the ATP-dependent responses shown in Figure 2, the *espC* mutant triggered a higher percent of Ca^2+^ responding cells than wild-type EPEC (Figure 3C). Also, the response amplitude was higher for the *espC* mutant with an average amplitude corresponding to 7.7 ± 0.4% (mean ± SEM) of the maximal agonist response, compared to 5.4±0.4% (mean ± SEM) for wild-type EPEC (Figure 3E). These responses occurred repeatedly at a high frequency of up to 4.5 responses per minute during several minutes following bacterial challenge (Figure 3D and F) and were dependent on Type III Secretion, as evidenced by the lack of response in cells infected with the *ΔescN* strain (Figure 3B and C). EPEC-induced fast Ca^2+^ responses were dependent on Ca^2+^ release since they were inhibited by U73122, a PLC inhibitor (Figure 3C and D). These similarities with the EPEC-induced eATP-dependent Ca^2+^ responses suggested that the atypical fast responses were triggered by low amounts of ATP released in the extracellular medium following insertion in host cell plasma membranes of discrete numbers of EPEC T3SS translocons. Consistently, these atypical EPEC-induced fast responses were abolished in the presence of the ATP receptor inhibitor Suramin (Figure 3C and D).

Together, these results show that at the onset of infection, EPEC induces an atypical pattern of fast Ca^2+^ responses involving the whole or a large area of the cell, likely resulting from low ATP levels released by the insertion of a discrete number of translocons in host cell membranes.

### EPEC-induced fast Ca^2+^ responses are triggered by low ATP levels

Previous studies have described local Ca²^+^ increases triggered by submaximal agonist concentrations and leading to limited IP_3_-mediated Ca^2+^ release. These local Ca²^+^ responses are typically small, of short durations, and localized to subcellular regions. Among these, the so-called ‘Blips’ correspond to elementary events of opening of a single IP_3_ receptor channel usually lasting between 50 and 100 ms, whereas ‘Puffs’ involve the synchronized activation of multiple IP_3_ receptor channels in localized clusters and last several hundreds of ms (Swillens et al., 1999). In contrast to these described local Ca^2+^ signals, EPEC-induced fast and small responses could occur throughout the cell, suggesting the coordination of elementary responses over large cell area. Since our findings suggested that these responses were elicited by low amounts of eATP released by a discrete number of T3SS translocons, we investigated whether low ATP levels could elicit similar responses.

**Figure 4.**
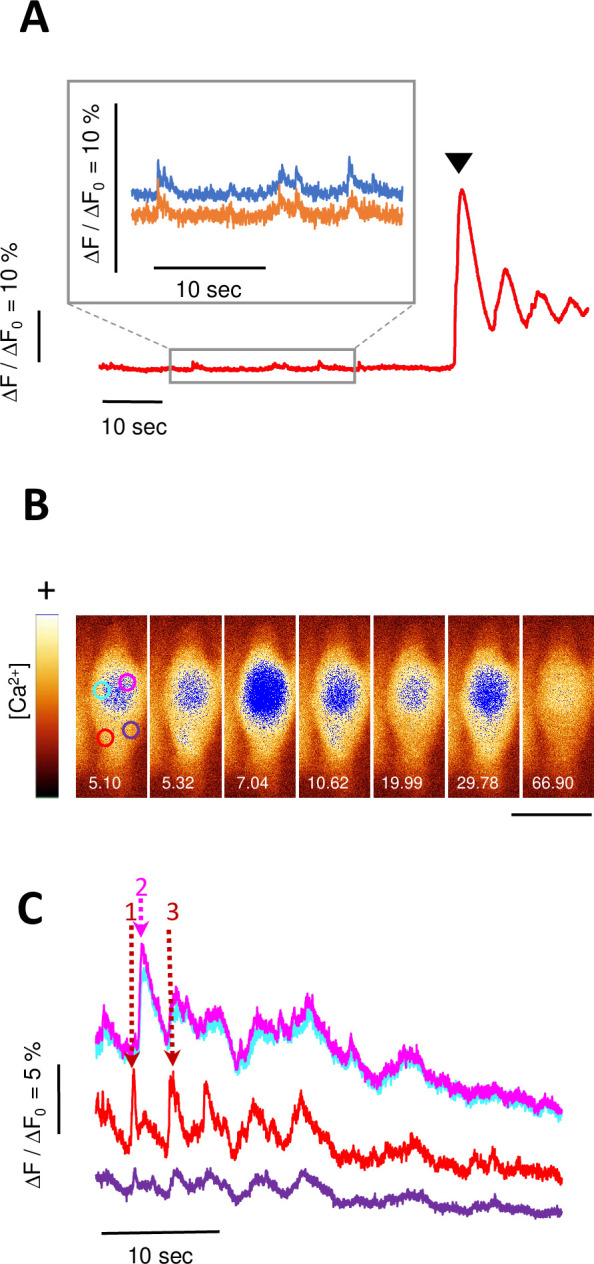
Enteropathogenic *Escherichia coli* (EPEC)-induced coordinated elementary Ca^2+^ responses are reproduced by low ATP levels. (**A–C**) HeLa cells were loaded with the fluorescent indicator Cal-520, challenged with 150 nM ATP and subjected to Ca^2+^ imaging. Image acquisition every 52 ms (**A**) or 22 ms (**B, C**). (**A**) Traces of Ca^2+^ variations corresponding to a single cell (red trace), or subcellular regions within the same cell (inset). (**A**) arrowhead: challenge with 2 μM ATP. (**B**) Time series of fluorescent micrographs pseudocolored using the ‘glow’ Fiji lookup table, where the blue pixels correspond to an arbitrarily set threshold value. The numbers indicate the elapsed time in seconds. Blue: high-intensity pixels showing the large top cell area with CCRICs and the local lower puff area. Scale bar = 10 µm. (**C**) Traces corresponding to Ca^2+^ variations in the subcellular regions depicted in Panel **B**. The responses are labelled 1–4, with the response 1 corresponding to the puff (Panel **B**, red ROI) impulsing the response 3 in the same region. Responses 2 and 4 correspond to CCRICs in Panel **B**, blue and green ROIs. Note the diffusion of the responses from the initial release area in other areas inferred from the dampening of the response amplitude.

HeLa cells treated with 150 nM ATP showed Ca^2+^ responses that were indistinguishable from fast responses elicited by EPEC, with an average percent of Ca^2+^ responding cells of 61.2±5.8% (mean ± SEM) and a frequency of 3.9 responses per cell over 60 s (Figure 4A, C, Figure 4—figure supplement 1A). These fast Ca^2+^ responses had an amplitude that did not exceed 10% of the maximal agonist response and occurred over several minutes (Figure 4A), with a duration of 2.1±1.0 sec (mean ± SEM) (N=4, 128 responses). As observed for EPEC, fast Ca^2+^ responses induced by low ATP levels involved the whole cell or large cell area encompassing the nuclear and perinuclear area and corresponding to at least 30% of the cell area as illustrated in Figure 4B. In this large area, all ROIs corresponding to 1 square micron showed a superimposable profile, as illustrated by traces in Figure 4C. Similar fast and small coordinated Ca^2+^ responses were also observed when cells were challenged with 100 nM of histamine, another Ca^2+^ agonist, with 44±7% (mean ± SEM) of cells showing responses, suggesting that these are generic responses triggered by low levels of IP_3_ (N=2, cells = 340; Figure 4—figure supplement 1). EPEC and low concentrations of eATP induced similar responses in polarized intestinal epithelial cells (Figure 4—figure supplement 2).

**Figure 4—figure supplement 1.**
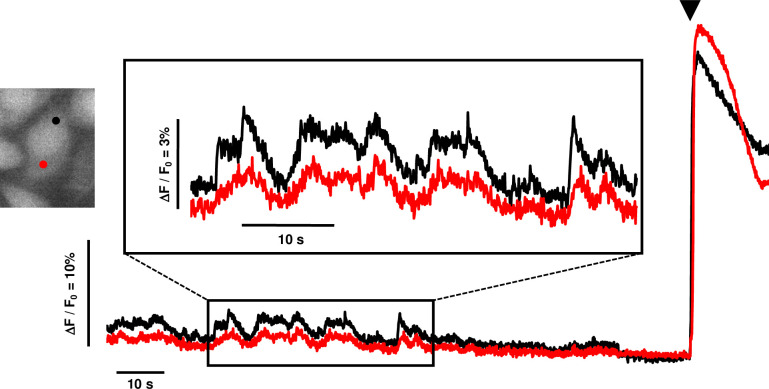
Low concentrations of histamine also induce small and fast coordinated Ca^2+^ responses. HeLa cells were loaded with the fluorescent indicator Cal-520, challenged with 100 nM histamine and subjected to Ca^2+^ imaging, with image acquisition performed every 57 ms. Left: fluorescence micrograph of the single cell with black and red ROIs corresponding to the traces of Ca^2+^ variations in matching color. The box corresponds to a higher magnification of the inset in traces shown at the bottom. Arrowhead: challenge with 10 μM histamine. Traces are representative of 340 cells analyzed from two independent experiments, with a frequency of 4.5±0.4 peaks·min⁻¹ (mean ± SEM) and peak duration of 4.45±0.19 s (mean ± SEM).

**Figure 4—figure supplement 2.**
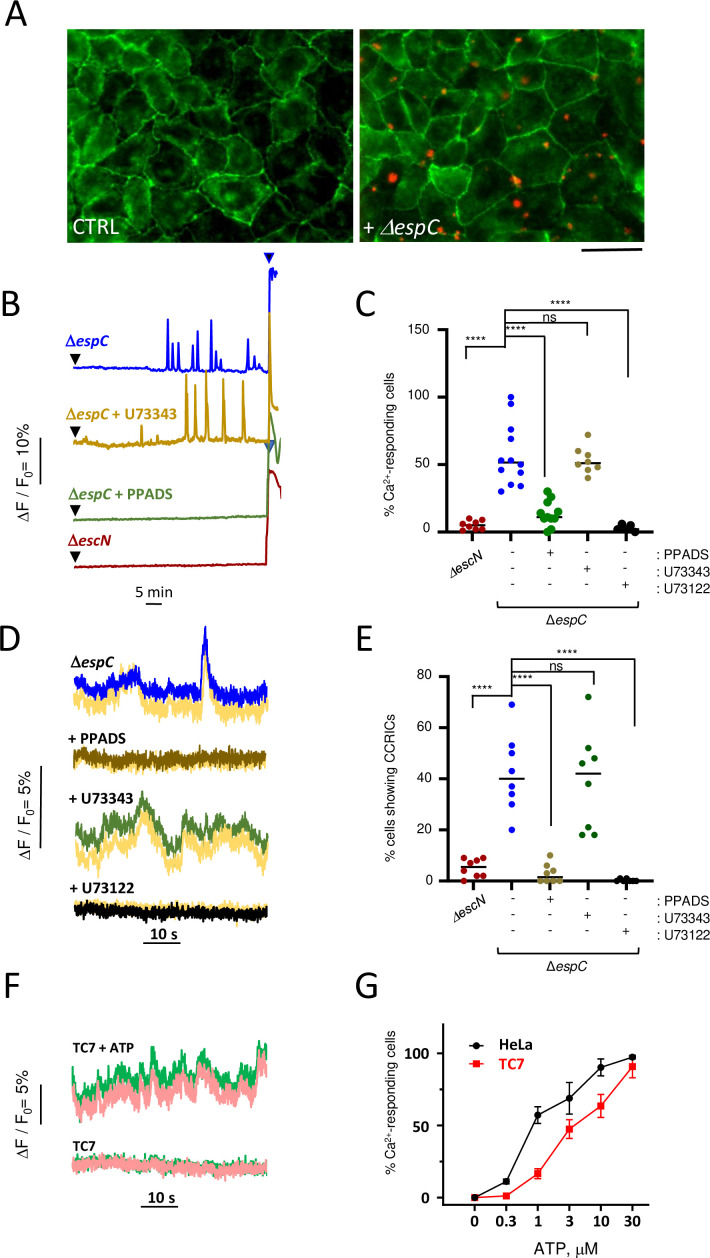
Enteropathogenic *Escherichia coli* (EPEC) induces CRICCs in polarized intestinal cells via eATP release. Polarized TC7 cells were loaded with the fluorescent indicator Cal-520 were challenged with the EPEC *ΔespC* strain expressing the red fluorescent protein (RFP) (**A-E**) or with ATP (**F, G**). (**A**) Representative confocal micrographs. Samples were fixed and processed for fluorescence microscopy analysis. Green: ZO-1 staining. Red: RFP fluorescence. Scale bar = 10 µm. (**B-G**) Samples were subjected to live-cell Ca^2+^ imaging at a frequency of one acquisition every 5 s. (**B, D**) Representative traces of Ca^2+^ variations in single cells challenged with the indicated strain and inhibitor. The black arrowheads indicate the time of bacterial challenge. The blue arrowhead indicates stimulation with 3 µM histamine. (**C, E**) Percent of cells exhibiting Ca^2+^ responses (**C**) (N=4, cells >241) or CCRICs (**E**) (N=3, cells >66) following challenge with the indicated bacterial strain and inhibitor. Each value corresponds to a replicate. Mann–Whitney test. ****p<0.0001. (**F**) Representative traces of Ca^2+^ variations following challenge with 150 nM ATP (TC7+ATP) or in buffer alone (TC7). The pink and green traces correspond to Ca^2+^ variations in ROIs within the same cell. (**G**) Percent of cells exhibiting Ca^2+^ responses following challenge with the indicated ATP concentration. Each value represents the mean ± SEM of at least 140 cells in three independent experiments. Figure 4—figure supplement 2—source data 1.All the source data for the graphs (C,E,G) in Figure 4—figure supplement 2.

**Figure 4—figure supplement 3.**
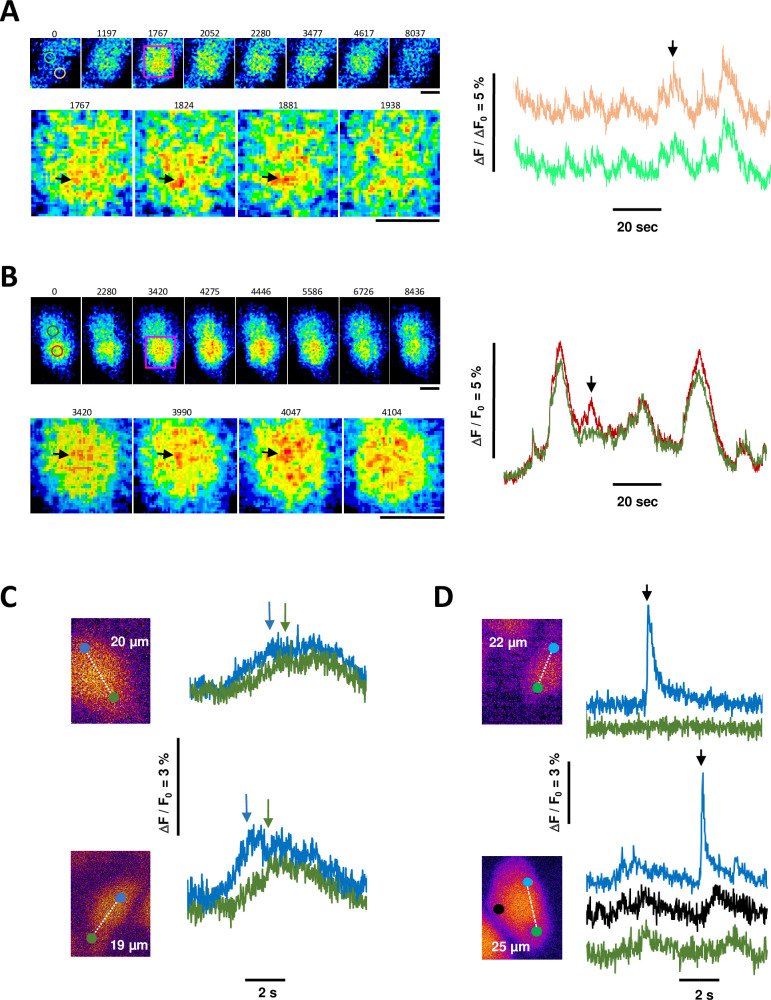
CCRICs induced by enteropathogenic *Escherichia coli* (EPEC) result from Ca^2+^ released by highly transient or mobile IP_3_R clusters. HeLa cells were loaded with Cal-520 and challenged with wild-type EPEC at a multiplicity of infection (MOI) of 50 bacteria/cell. (**A, B**) Globally coordinated responses associated with small and highly mobile clusters. **Time series micrographs**, Cal-520 intensity depicted in pseudocolor. Numbers: time in ms. Lower panels are magnification of the purple box in the upper panel, with a time interval of 57 ms. Scale bar = 5 μm. Note the high mobility of small clusters/channels. The black arrows point at larger and less mobile clusters. **Traces**, variations of Ca^2+^ in ROI depicted at T=0 in the micrographs in the corresponding color. The black arrow points at the response peak illustrated by the time series. (**C, D**) micrographs, Cal-520 fluorescence depicted in the Fiji red lute. Images were taken every 22 ms. Solid circles: ROIs where the variations of Ca2+ are shown in traces in the corresponding panel on the right. The blue ROI corresponds to the Ca2+ release source based on the higher response amplitude, and the green ROI to a distal region. The number indicates the distance between the ROIs shown by the dashed line. (**C**) Arrows point at the peak of the response in the ROI with the corresponding color. Note the delay in response elicitation between the source- (blue) and distal (green) ROI. (**D**) Puff-like responses (black arrowhead). Note the absence of Ca^2+^ variations in the distal region (Top), and the simultaneous elicitation of a puff and CCRICs in different ROIs (Bottom).

**Figure 4—figure supplement 4.**
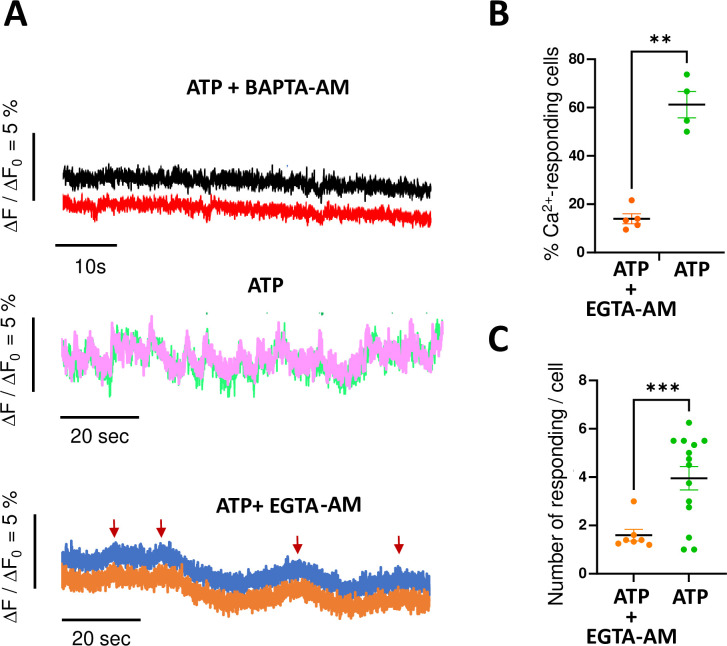
Intracellular Ca^2+^ chelation inhibits CCRICs. HeLa cells were loaded with the fluorescent indicator Cal-520 in the presence or absence of 20 μM EGTA-AM or BAPTA-AM. Samples were stimulated with 150 nM ATP and subjected to high-speed Ca^2+^ imaging at a frequency of one acquisition every 22 ms. (**A**) Representative traces of Ca^2+^ variations in two subcellular regions of the same cell in samples treated in the presence or absence of the indicated inhibitor. No Ca^2+^ responses were observed for cells treated with BAPTA-AM (N=3, >65 cells). EGTA-AM treatment led to an inhibition of Ca^2+^ responses, associated with small variations in the Ca^2+^ baseline that were arbitrarily scored as flattened Ca^2+^ pseudo-responses (ATP + EGTA AM, red arrows). (**B**) Percent of cells exhibiting Ca^2+^ responses (N>3, cells >62). (**C**) Average number of responses per cell. (N>3, n>62). Mann–Whitney test. **p<0.01; ***p<0.001. Figure 4—figure supplement 4—source data 1.All the source data for the graphs (B,C) in Figure 4—figure supplement 4.

More detailed scrutinizing showed that in their initial mounting phase, these fast Ca^2+^ responses were created by the opening of discrete clusters involving an area of ca. 0.04 μm^2^ that had a transient activity, or possibly were highly mobile, since they were seldom detected at a similar location for three consecutive 22 ms acquisition frames (Figure 4—figure supplement 3A, B). These discrete clusters showed similar Ca^2+^ kinetics suggesting the coordination of Ca^2+^ release of single IP_3_R clusters throughout the area that we will hereafter termed CCRICs for ‘Coordinated Ca^2+^ Responses from IP_3_R Clusters.’ Treatment with BAPTA-AM to chelate intracellular Ca^2+^ led to a complete inhibition of CCRICs (N=3, n>150 cells; Figure 4—figure supplement 4A). Ca^2+^ responses could still be detected upon cell treatment with EGTA-AM consistent with its lower k_on_ rate for Ca^2+^, but with a significant inhibition of the percentage of Ca^2+^ responding cells as well as of the frequency of responses per cell, suggesting that coordination could occur via Ca^2+^ diffusion and Ca^2+^-induced Ca^2+^ release (Figure 4—figure supplement 4).

In rare instances (less than 3%), typical local ‘Puff’ responses elicited by these ATP concentrations could also be detected often occurring at the cell periphery (Figure 4B, red region and 4 C, red arrow; Figure 4—figure supplement 3D, blue trace) (N>20, cells >500). As expected from the small concentrations of Ca^2+^ released at puff sites, no increase in cytosolic Ca^2+^ was detected in a distal cell region (Figure 4—figure supplement 3D, top), indicating that isotropic Ca^2+^ diffusion from a puff release site cannot account for Ca^2+^ increase over large cell area. Puffs could also be detected concomitantly with CCRICs in different ROIs of the same cell (Figure 4—figure supplement 3D, bottom). In contrast to puffs, CCRICs often showed responses of comparable amplitude in distal regions over the whole cell (Figure 4C, Figure 4—figure supplement 3A, B), suggesting the contribution from IP_3_R cluster activation by Ca^2+^-induced Ca^2+^ release (CICR). Within a given cell, the vast majority of CCRICs appeared quasi-synchronized at the fastest acquisition rate of 22 ms/frame that we could achieve. However, in few instances, a delay could be detected in the elicitation of a peak in distant region of a cell (Figure 4—figure supplement 3C). These observations suggest that the quasi-synchronization of CCRICs result from the fast diffusion of Ca^2+^ leading to the activation of IP_3_R clusters over large cell area, which may be delayed in some instances. Scrutinizing of CCRICs showed that while their profiles were comparable, the amplitude of these responses varied in different regions of the cell, with often a single 1 μm^2^ region, likely corresponding to the initial firing cluster, showing a prominent amplitude and other regions with smaller amplitude for a given response (Figure 4B and C). For example, in Figure 4C, the highest amplitude is observed in the red region for peaks 1 and 3, whereas it is observed in the purple region for peak 2. Thus, for a given CCRIC, the respective contribution of local IP_3_R cluster activation and isotropic diffusion of Ca^2+^ from other release sites in Ca^2+^ increase may vary in different regions of the cell.

### CCRICs are coordinated by the rapid diffusion of Ca^2+^ at low concentrations in cell area with a high density of IP_3_ clusters

**Figure 5.**
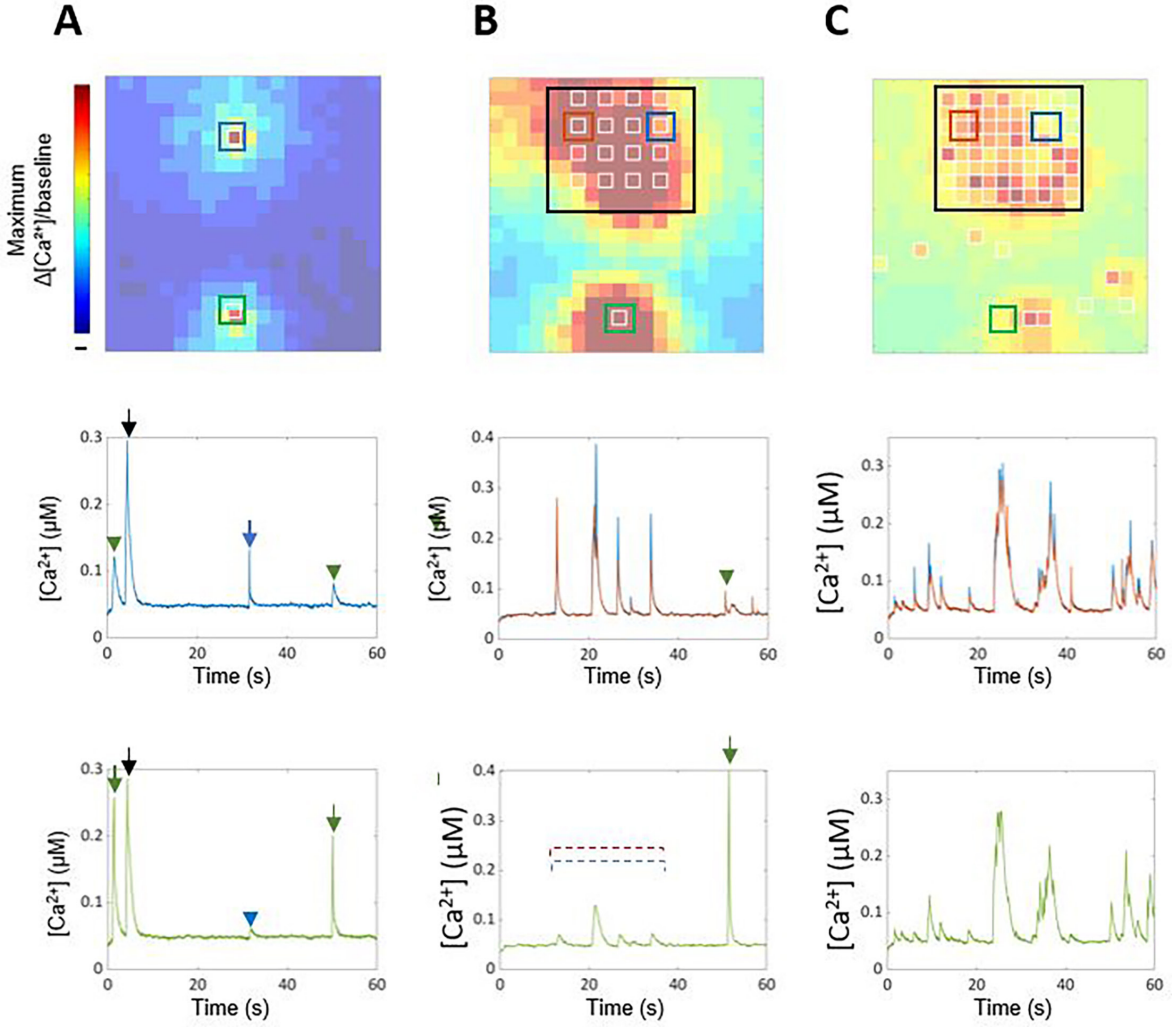
Modeling of coordinated elementary Ca^2+^ responses. **Top**, Ca^2+^ variations in subcellular area within a single cell are represented in pseudocolor. Shown are the maximum values of Δ[Ca^2+^]/[Ca^2+^]_b_ reached in each compartment during a 60 s simulation. Empty white squares: IP_3_R clusters. **Graphs**: traces correspond to Ca^2+^ variations in the region with the matching color. Colored arrows: Ca^2+^ response due to the activation of an IP_3_ cluster in the region with the matching color. Colored arrowhead and dashed red and blue lanes: Ca^2+^ variations due to the diffusion of a Ca^2+^ response from or nearby to the region with the matching color. Black arrows: Ca^2+^ response due Ca^2+^-activated Ca^2+^ release. (**A**) Low density of IP_3_R clusters with local responses detected. (**B, C**) Empty black box: area with a high density IP_3_R clusters. (**C**) similar to **B**, but following IP_3_R cluster sensitization due to increased Ca^2+^ responses.

In Figure 5, we used modeling to further investigate the mechanism of coordination of these fast Ca^2+^ responses. Based on our previous studies (Voorsluijs et al., 2019; Ornelas-Guevara et al., 2023), the model provides a fully stochastic spatial description of Ca^2+^ release dynamics from IP_3_R clusters in a two-dimensional representation of a HeLa cell. The simulation domain extends on 10×10  µm^2^ and is discretized into a 20×20 grid of compartments (0.5×0.5  µm^2^ each), each representing a cytosolic subvolume of 10^-16 ^L. Each compartment contains at most one cluster of IP_3_R, whose dynamics is described as a whole. Besides, cytosolic Ca^2+^ concentration can also vary because of uptake by SERCA, release by a leak, or diffusion (Appendix 1).

In Figure 5A, low ATP levels lead to low IP_3_ levels activating a limited number of IP_3_ clusters, opening stochastically and releasing small amounts of Ca^2+^. In a given area of the cell, the Ca^2+^ variation integrates Ca^2+^ release from clusters within this area, as well as Ca^2+^ diffusing from or to other cell area. For a given response, the initially firing cluster is contained in an area characterized by the highest Ca^2+^ peak amplitude (Figure 5A, blue and green arrows) that dampens in a distal area (Figure 5A, blue and green arrowheads). If the density of IP_3_R clusters is low, as expected for ER compartment at the cell periphery, the spatial segregation of the initial firing cluster and resulting Ca^2+^ diffusion to other area is clearly detected (Figure 5A). In instances, however, the model predicts temporally coordinated responses of similar amplitude, suggesting Ca^2+^-induced Ca^2+^ release from secondary clusters (Figure 5A, black arrow). For both types of Ca^2+^ dynamics, a large value of the Ca^2+^ diffusion coefficient of 100 μm^2^/s is a key parameter that needs to be taken into account in the model. While it is generally admitted that Ca^2+^ diffuses very slowly due to the Ca^2+^ buffers in the cell (~30 μm^2^/s), the low levels of Ca^2+^ released by CCRICs may not be subjected to the diffusion limitations observed at higher Ca^2+^ levels, because of the relatively moderate affinity of buffers for Ca^2+^. How the effective diffusion coefficient of Ca^2+^ is affected by the Ca^2+^ concentration is explained in more detail in Appendix 2.

If the IP_3_R cluster density is high, as expected in the large perinuclear and nuclear area corresponding to the bulk of the ER, the coordination between individual clusters is very fast (Figure 5B). As a result, the identification of initial firing clusters goes beyond the technical capacities of the imaging set-up, and ROI within this area show comparable profiles of fast Ca^2+^ responses (Figure 5B). Upon prolonged incubation with increasing Ca^2+^ responses and sensitization of IP_3_R clusters, the coordination of responses linked to Ca^2+^-induced Ca^2+^ release becomes predominant throughout the cell (Figure 5C). Moreover, at high IP_3_ concentrations, the model reproduces the propagation of a large-amplitude Ca^2+^ wave, as expected (Appendix 3).

### Low eATP levels dampen NF-κB activation

Our findings indicate that the novel Ca²^+^ response pattern is not exclusive to EPEC infection and can be replicated by low levels of eATP or histamine, suggesting a broader physiological relevance. From an immunological perspective, CCRICs may, therefore, play a critical role in various signaling pathways during bacterial infection. eATP is a well-characterized danger signal contributing to the elicitation of pro-inflammatory signals in various tissues in response to infections (Savio et al., 2018). Previous studies linked intracellular Ca²^+^ signaling and NF-κB activation, a key transcription factor that triggers inflammatory responses (Smedler and Uhlén, 2014). We, therefore, set up to investigate the effects of CCRICs triggered by low eATP levels on NF-κB activation, by performing Western blot analysis against the phosphorylated forms of IκBα (p- IκBα) and P65 (p-P65).

**Figure 6.**
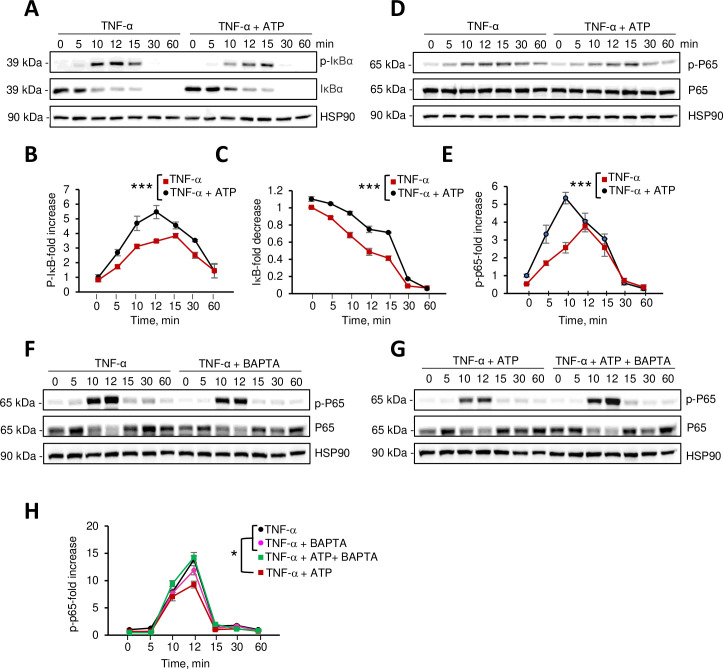
Low ATP levels dampen NF-kappaB activation. HeLa cells were stimulated with 10 ng/ml TNF-α alone or in the presence of 150 nM ATP or 20 μM BAPTA-AM (**F–H**). At the indicated time points, cell lysates were analyzed by Western blot using the indicated antibodies. (**A, D, F, G**) Representative blots. (**B, C, E, H**) Densitometry analysis of the indicated antibody signal normalized to that of HSP90 (**B, C**) or total P65 (**E, H**), expressed as fold-increase to basal levels of p-IκB (**B**), IκB (**C**), or p-p65 (**E, H**) at time = 0. Values correspond to the mean ± SEM of three or four independent experiments. p-p65: anti-phospho P65 antibody. p-IκB: anti-phospho IκB antibody. ANCOVA test. *p<0.05; **p<0.01; ***p<0.001. Figure 6—source data 1.PDF file containing original Western blots for Figure 6A, D, F and G, indicating the relevant bands and treatments. Figure 6—source data 2.Original files for western blot analysis displayed in Figure 6A, D, F and G. Figure 6—source data 3.All the source data for the graphs (B,C,E,H) in Figure 6.

As shown in Figure 6A and B, in control samples, TNF-α induced IκB-α phosphorylation peaking 10 min following challenge. In contrast, in the presence of low ATP levels, IκB-α showed a delayed phosphorylation with a 2.1-fold decrease at 10 min post-challenge (Figure 6A and B). Consistently, the rates of IκB-α degradation were also slower in the presence of ATP relative to control (Figure 6A and C). As expected from the IκB-α results, TNF-α induced the phosphorylation of the NF-κB P65 subunit and ATP led to a delay and decrease in P65 phosphorylation (Figure 6D and E).

**Figure 6—figure supplement 1.**
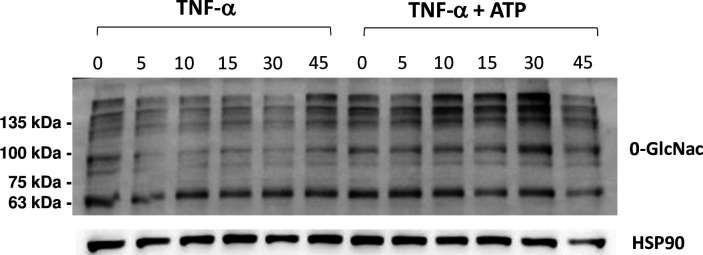
Effects of ATP on TNF-α-induced profiles of O-GlcNacylation in HeLa cells. HeLa cells were stimulated with 10 ng/ml TNF-α alone or co-stimulated with 10 ng/ml TNF-α and 150 nM ATP. Representative blots of cell lysates analyzed by Western blot at the time points indicated in minutes using the indicated antibodies. Figure 6—figure supplement 1—source data 1.PDF file containing original Western blots for Figure 6—figure supplement 1 indicating the relevant bands and treatments. Figure 6—figure supplement 1—source data 2.Original files for Western blot analysis displayed in Figure 6—figure supplement 1.

In control experiments, we did not detect differences in P65 phosphorylation in response to TNF-α stimulation when cells were treated with BAPTA-AM to chelate intracellular Ca^2+^ (Figure 6F and H). However, cell treatment with BAPTA-AM prevented the dampening of P65 phosphorylation triggered by low ATP levels (Figure 6G and H), suggesting that CCRICs down-regulated TNF-α-induced NF-κB activation.

### Low eATP levels down-regulate NF-κB activation through Ca^2+^-dependent O-GlcAcylation

We next investigated how CCRICs could regulate NF-κB activation. O-linked β-*N*-acetylglucosamine (O-GlcNAc) transferase (OGT) was reported to regulate NF-κB signaling by post-translationally modifying the p65 subunit (Ruan et al., 2017). Interestingly, OGT is regulated by Ca^2+^ signaling, suggesting that CCRICs could affect NF-κB activation via O-GlcNAcylation.

**Figure 7.**
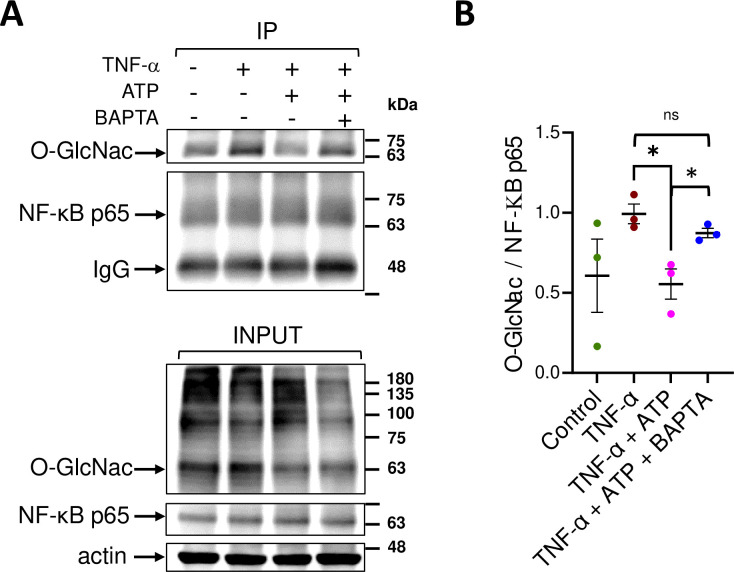
Low ATP levels down-regulate NF-kB O-GlcNAcylation in a Ca^2+^-dependent manner. HeLa cells were stimulated with 10 ng/ml TNF-α alone or in the presence of 150 nM ATP with or without 20 μM BAPTA-AM for 12 min. Cell lysates were subjected to P65 immunoprecipitation. (**A**) Representative blots with the indicated antibodies. IP: immunoprecipitates; L: total cell lysates. (**B**) Densitometry analysis of the O-GlucNAc signal in P65 immunoprecipitates normalized to that of TNF-α alone. Mann–Whitney test. N=4. *p<0.05. ns: not significant. Figure 7—source data 1.PDF file containing original Western blots for Figure 7 (IP and Input) indicating the relevant bands and treatments. Figure 7—source data 2.Original files for Western blot analysis displayed in Figure 7 (IP and Input).

As shown in Figure 6—figure supplement 1, TNF-α in the presence of 150 nM eATP stimulated the levels of O-GlcNacylation, specifically for proteins with an apparent molecular weight superior to 100 kDa that was not observed with TNF-α alone. To further investigate the effects of low eATP levels on NF-κB O-GlcNacylation, we performed immunoprecipitation of P65 RelA on lysates of cells stimulated for 12 min with TNF-α alone or co-stimulated with TNF-α and 150 nM eATP. As shown in Figure 7, TNF-α induced increased O-GlcNacylation of P65 relative to non-stimulated cells, but this increase was inhibited by low eATP levels. Inhibition of P65 O-GlcNacylation by eATP was Ca^2+^-dependent, since it was not observed in the presence of BAPTA-AM (Figure 7).

The results indicate that low eATP levels inhibit O-GlcNacylation of P65 induced by TNF-α in a Ca^2+^-dependent manner and suggest that differential O-GlcNacylation of P65 relative to higher molecular weight proteins.

## Discussion

We report here the characterization of CCRICs, corresponding to yet undescribed Ca^2+^ responses. CCRICs showed rapid kinetics with an average duration of ca 2.1 s and amplitude corresponding to an increase in Ca^2+^ cytosolic concentration of a few hundred nM, seemingly smaller than that of puffs (Figure 4—figure supplement 3D), often occurring repeatedly with a frequency of up to 12 CCRICs/min over the whole cell. Our modeling studies support the notion that CCRICs implicate the rapid coordination of IP_3_R clusters via CICR in large cell area, challenging established concepts in the Ca^2+^ signaling field.

Ca²^+^ diffusion in the cytosol is regulated by mobile and immobile Ca²^+^-binding proteins acting as buffers and forming localized microdomains with steep Ca²^+^ concentration gradients. At the mouth of an open IP_₃_R channel, Ca²^+^ concentration can reach 100 μM, while just 1–2 μm away, it may drop below 1 μM. Diffusion is restricted by Ca^2+^ buffers with K_D_’s that are generally lower than this concentration. Consequently, Ca²^+^ signaling is generally admitted to be spatially restricted, typically influencing regions within approximately 5 μm of the release site (Foskett et al., 2007). The distribution of Ca²^+^-binding proteins and the spatial arrangement of release channels allow IP₃R-mediated [Ca²^+^]_i_ signals to exhibit diverse spatial and temporal properties, making this system highly adaptable (Vandeput et al., 2007). High-resolution optical imaging of fluorescent Ca²^+^ indicators in intact cells indicates that IP_₃_-mediated [Ca²^+^]_i_ signals are structured levels (Foskett et al., 2007). At low IP₃ levels, individual IP₃R opens stochastically at discrete release sites, causing localized elevations in cytoplasmic [Ca²^+^]. At higher IP₃ levels, Ca²^+^ release spreads between IP₃R clusters, propagating waves that travel at tens of microns per second, coordinating intracellular signaling ultimately leading to a global Ca²^+^ response. In reference models describing intracellular Ca^2+^ dynamics, cell regions with a high IP_3_R density initiate the Ca^2+^ response from which Ca^2+^ waves propagate with a diffusion coefficient of 10–30 μm^2^/s (Falcke, 2003).

In contrast to these described global and local Ca²^+^ responses, we found CCRICs to be highly temporally coordinated over large area, suggesting the fast diffusion of Ca^2+^ and propagation of Ca^2+^ by CICR. While challenging generally admitted concepts on the poor diffusion of Ca^2+^, this view is fully supported by our theoretical modeling implicating the fast diffusion of Ca^2+^, with a diffusion coefficient of at least 100 μm^2^/s that can be expected at low cytoplasmic [Ca²^+^]. Indeed, at these low Ca²^+^ concentrations not exceeding a few hundred nM, the majority of Ca²^+^ buffers are not expected to efficiently bind to Ca²^+^ and to significantly interfere with Ca²^+^ diffusion because of their relative low affinity.

We found that CCRICs implicate a large cell area, including the nuclear and perinuclear area. The large CCRIC area likely involves the bulk of the endoplasmic reticulum, while peripheral area may contain smaller ER compartments. In our model considering fast Ca^2+^ diffusion when buffers are far from saturation, the higher density of IP_3_R clusters in the CCRIC area accounts for the high coordination of the responses, relative to the lack of coordination in peripheral area where lower IP_3_R density is expected. Consistently, while CRICCs were detected in the vast majority of cells at these very low agonist concentrations, in rare instances, local ‘puff-like’ responses were also detected at the cell periphery. These observations are in contrast to previously described Ca^2+^ puffs preceding global responses reported to occur preferentially in perinuclear area (Thomas et al., 2000). These earlier studies, however, involved higher agonist concentrations (1–5 μM ATP) expected to lead to the release of higher IP_3_ concentrations, which may preferentially stimulate larger IP_3_R clusters at the perinuclear region because of the higher density of IP_3_Rs. In addition, larger IP_3_ clusters may release higher amounts of Ca^2+^ for which, as opposed to CCRICs, diffusion would be restrained by Ca^2+^ buffers, thereby favoring the spatial confinement of the response.

We showed that a low dose of eATP triggering CCRICs delayed and dampened NF-κB activation linked to a reduction in O-GlcNAcylation of the NF-κB p65 subunit. One major open question is the mechanism by which CCRICS down-regulate NF-κB activation. NF-κB activity can be modulated by O-GlcNAcylation, a reversible glycosylation modification catalyzed by O-GlcNAc transferase (OGT) and O-GlcNAcase (OGA) (Liu and Ramakrishnan, 2021). Previous studies demonstrated that Ca²^+^ signals activate Ca²^+^-regulated enzymes like CaMKII, which in turn phosphorylates and activate OGT, promotes O-GlcNAcylation (Ruan et al., 2017). In other studies, OGT-mediated O-GlcNAcylation could modulate NF-κB signaling pathway (Dong et al., 2023). O-GlcNAcylation of P65 could also inhibit its interaction with IκB-α, promote p65 nuclear translocation, and increase NF-κB transcriptional activity (Liu and Ramakrishnan, 2021). Reduced O-GlcNAcylation of P65 at residues S550 and S551 was shown to result in decreased NF-κB activation and nuclear translocation (Motolani et al., 2023). These findings are in line with our results suggesting that CCRICs elicited by low-level eATP, downregulate p65 O-GlcNAcylation and NF-κB activation, possibly by modulating OGT activity or OGT-p65 interactions. Reduced O-GlcNAcylation of p65 may affect its phosphorylation patterns indirectly, perhaps by altering the interaction of NF-κB with kinases or phosphatases involved in its activation (Özcan et al., 2010).

Our findings indicate that eATP differentially regulates inflammatory signaling pathways in epithelial cells by dampening NF-κB activation at low levels and stimulating its activation at high concentrations. These results extend the key role of eATP from a danger-associated molecular pattern (DAMP) to a fine-tuner of inflammatory responses depending on its concentration.

## Materials and methods

**Key resources table:** 

Reagent type (species) or resource	Designation	Source or reference	Identifiers	Additional information
Strain, strain background (Wild-type Enteropathogenic *Escherichia coli*)	EPEC WT	Levine et al., 1985, DOI: https://doi.org.10.1093/infdis/152.3.550	Strains WT E2348/69	
Strain, strain background (Enteropathogenic *Escherichia coli* escN mutant)	*ΔescN*	Garmendia et al., 2004, DOI: https://doi.org/10.1111/j.1462-5822.2004.00459.x		*escN* isogenic mutant of WT E2348/69
Strain, strain background (Enteropathogenic *Escherichia coli* espC mutant)	*ΔespC*	Guignot et al., 2015, DOI: https://doi.org.10.1371/journal.ppat.1005013		*espC* isogenic mutant of WT E2348/69
Cell line (*Homo sapiens*)	HeLa	ATCC	ATCC CCL2	RRID:CVCL_0030
Cell line (*Homo sapiens*)	Caco2/TC7	Chantret et al., 1994, DOI: https://doi.org/10.1242/jcs.107.1.213		
Antibody	anti-Z0-1 polyclonal antibody	Thermo Fisher Scientific	Cat# 40–2200 RRID:AB_2533456	IF (1:50)
Antibody	NF-κB p65 Rabbit monoclonal antibody	Cell Signaling Technology	Cat# 8242 RRID:AB_10859369	WB (1:1000)
Antibody	Phospho-NF-κB p65 Rabbit monoclonal antibody	Cell Signaling Technology	Cat# 5733 RRID:AB_10706937	WB (1:1000)
Antibody	Phospho-IκB-α Mousse monoclonal antibody	Cell Signaling Technology	Cat# 4088 RRID:AB_1904009	WB (1:1000)
Antibody	IκB-alpha Rabbit polyclonal antibody	Cell Signaling Technology	Cat# 9242 RRID:AB_331623	WB (1:1000)
Antibody	anti-rabbit IgG-Alexa 488	Thermo Fisher Scientific	Cat# A-11034 RRID:AB_2576217	IF (1:200)
Antibody	HSP90 Mousse monoclonal antibody	Santa Cruz Biotechnologies	#sc-13119 RRID:AB_675659	WB (1:1000)
Antibody	NF-kB p65 Rabbit polyclonal antibody	Abcam	ab16502 RRID:AB_443394	WB (1:1000)
Antibody	O-GlcNAc Mousse monoclonal antibody	Abcam	ab2739 RRID:AB_303264	
Antibody	Actin Rabbit polyclonal antibody	Cell Signaling Technology	Cat# 4967 RRID:AB_330288	(1:200)
Other	pRFP	Addgene	# 26924 RRID:Addgene_26924	Plasmid
Chemical compound, drug	Alexa Fluor 488 Phalloidin	Cell Signaling	#8878	Fluorescent indicator dye (1:400)
Chemical compound, drug	Cal-520	AAT Bioquest	AAT #21130	Fluorescent indicator dye
Software, algorithm	Prism7	Graphpad	RRID:SCR_002798	
Other	DAPI stain	Merck	#102362276001	(1 µg/ml)

### Cell and bacterial culture

HeLa cells were from ATCC (RRID:CVCL_0030). Human colon adenocarcinoma Caco-2/TC-7 cells were a gift from M. Rousset, whose team isolated the cell line (Chantret et al., 1994). We did not confirm the identity of these cells. HeLa cells and Caco-2/TC-7 cells were maintained in Dulbecco’s Modified Eagle Medium (DMEM; Gibco, Thermo Fisher Scientific) supplemented with 10% fetal bovine serum (FBS; Gibco, Thermo Fisher Scientific) and DMEM containing 20% FBS, supplemented with non-essential amino acids, respectively. Cells were grown at 37  °C in a humidified incubator with 10% CO_₂_ and were devoid of mycoplasma. Wild-type Enteropathogenic *Escherichia coli* (EPEC WT), *ΔescN*, and *ΔespC* strains were cultured in Luria-Bertani (LB) broth at 37  °C with kanamycin at a final concentration of 15 μg/ml in a shaking incubator. All strains were transformed with the pRFP plasmid (Addgene plasmid # 26924), which encodes red fluorescent protein and carries a Kanamycin resistance gene for selection. When applicable, ampicillin (100  μg/ml) and kanamycin (50 μg/ml) were included. The red fluorescence enabled visualization of the bacteria during downstream analyses.

### EPEC infection of cells

HeLa cells were seeded in 6-well plates at a density of 4.5×10⁵ cells per well one day before infection. TC-7 cells were seeded at a density of 5×10⁵ cells/well in 6-well plates and allowed to polarize for 4 days prior to bacterial challenge, replacing medium every day. EPEC WT, *ΔescN,* and *ΔespC* strains grown in the exponential phase were resuspended and primed for 5 hr before challenging HeLa cells in DMEM medium. Cells were challenged with bacteria at an OD_600_=0.2 (Low MOI) or 0.8 (High MOI).

### Ca^2+^ imaging

HeLa cells were seeded onto 25 mm-diameter glass coverslips. Cells were preloaded with the fluorescent indicator dye Cal-520 (AAT #21130) for 30 min at room temperature, followed by two PBS washes and one time with DMEM. And placed coverslips, imaging was performed in an observation chamber in DMEM without phenol red, supplemented with 25 mM HEPES. Following a 3 min baseline acquisition, add the required bacteria strains and OD_600_ into the chamber. Following a 10 min incubation at room temperature to allow bacterial attachment. Imaging was then carried out at 35 °C to allow for bacterial type III secretion, using a Nikon Eclipse TE200 inverted fluorescence microscope with a 60× objective for 1 hr of infection. Fluorescence signals were acquired at 485 nm with excitation and 535 nm emission parameters. Image control and data acquisition were managed by Simple32 software (Compix Inc), depending on the experiment requirement, imaging at 22 ms, 57 ms or 10 s acquisition intervals. 3 μM of histamine and 2 μM of ionomycin were applied to check the ability of cells to show a calcium response. Images were captured using a CMOS camera (Hamamatsu) and analyzed using the same software.

### Immunofluorescence analysis

Cells were washed three times with PBS and fixed with 3.7% paraformaldehyde (PFA), permeabilized with 0.1% Triton X-100 for 5 min, and washed with PBS. Blocking was performed using 3% FBS in PBS for 30 min at room temperature. Cells were incubated with anti-Z0-1 polyclonal antibody (Thermo Fisher Scientific Cat# 40–2200, RRID:AB_2533456) for 1 hr at a 1:50 dilution in PBS containing 1% FBS for an hour, followed by anti-rabbit IgG-Alexa 488 (Thermo Fisher Scientific Cat# A-11034, RRID:AB_2576217) or Alexa Fluor 488 Phalloidin (Cell Signaling #8878) at a 1:200 dilution and DAPI (1 µg/ml; Merck, #102362276001) for another hour. Samples were mounted using Dako mounting medium (Agilent) and imaged using a Nikon Ti2 confocal microscope equipped with a 60× objective and Nikon acquisition software.

### Modeling

We developed a fully stochastic spatial model to simulate Ca^2+^ release dynamics from IP_3_R clusters in a two-dimensional representation of a HeLa cell. The simulation domain measures 10×10  µm^2^ and is discretized into a 20×20 grid of compartments (0.5×0.5  µm^2^ each), each representing a cytosolic subvolume of 10^-16 ^L. Ca^2+^ exchange between the endoplasmic reticulum (ER) and the cytosol occurs through IP_3_R-mediated release, SERCA uptake, and ER Ca^2+^ leak, following the framework by Voorsluijs et al., 2019 and Ornelas-Guevara et al., 2023. Ca^2+^ diffusion is implemented stochastically as a kinetic process between adjacent compartments (Kraus et al., 1996), with a diffusion coefficient of 100  µm^2^/s to reflect moderate endogenous buffering. Each IP_3_R cluster functions as a single unit with four possible states: Open (O), Closed (C), and two inhibited states (i1 and i2), transitioning in response to local [Ca^2+^] and [IP_3_]. This phenomenological description captures key characteristics of Ca^2+^ puffs and their transition to global signals via Ca^2+^ diffusion and calcium-induced calcium release.

We perform all simulations using the Gillespie algorithm (Gillespie, 1976) where each event, reaction or diffusion, is selected stochastically based on its propensity. See Appendices for additional information about the model.

### Western blot analysis

To obtain total cell extracts, cells were lysed in sample buffer 1 x (62.5 mM Tris pH = 8, 2% SDS, 10% glycerol, 0.05% bromophenol blue, 5% β-mercaptoethanol) and boiled at 95 °C for 5 min. Proteins from total lysates were separated by SDS PAGE and transferred to a nitrocellulose membrane (0.45 μM AmershamTM ProtranTM). Western blot analysis was performed according to standard procedure using the following primary antibodies diluted in PBS containing 0.1% Tween-20 and 5% non-fat milk: IκB-α (Cell Signaling Technology Cat# 9242, RRID:AB_331623) at a 1:1000 dilution, Phospho-IκB-α (Cell Signaling Technology Cat# 4088, RRID:AB_1904009) at a 1:1000 dilution, NF-κB p65 (Cell Signaling Technology Cat# 8242, RRID:AB_10859369) at a 1:5000 dilution, Phospho-NF-κB p65 (Cell Signaling Technology Cat# 5733, RRID:AB_10706937) at a 1:1000 dilution, HSP90 (Santa Cruz Biotechnologies #sc-13119, RRID:AB_675659) at a 1:1000 dilution. The secondary HRP-conjugated anti-mouse (Cytiva) and anti-rabbit (Sigma) antibodies were used at a 10^–4^ dilution.

### Immunoprecipitation assays

HeLa cells were seeded in 150 cm^2^ dishes (7.4×10⁶ cells/dish) and cultured in DMEM supplemented with 10% FBS. Cells were pretreated with 20 µM BAPTA for 30 min at room temperature where indicated, then stimulated with 10 ng/mL TNF-α alone or in combination with 150 nM ATP for an additional 12 min. After treatment, cells were washed with ice-cold PBS containing 1 mM NaF and 1 mM Na₃VO₄, lysed in ice-cold lysis buffer (50 mM Tris-HCl pH 7.5, 0.5% Triton X-100, 100 mM NaCl, 1 mM DTT, protease inhibitor cocktail without EDTA), and incubated on a rotating wheel at 4 °C for 1 hr. Lysates were clarified by centrifugation at 13,000 rpm for 30 min at 4 °C. Supernatants were incubated with 5 µl of anti-NF-kB p65 antibody (Abcam, ab16502, RRID:AB_443394) for 2 hr at 4 °C with rotation, followed by overnight incubation with pre-equilibrated protein A/G beads. Immunocomplexes were washed once with lysis buffer and twice with PBS, then eluted in Laemmli buffer by boiling at 100 °C for 5 min. Input and IP samples were analyzed by Western blotting using antibodies against O-GlcNAc (Abcam Cat# ab2739, RRID:AB_303264), anti-NF-kB p65, and actin (Cell Signaling Technology Cat# 4967, RRID:AB_330288).

### Statistical analysis

All quantitative data are presented as mean ± SEM from at least three independent experiments. Statistical significance was assessed using unpaired two-tailed Student’s t-tests with unequal variance, unless otherwise specified. GraphPad Prism 7 (GraphPad Software, RRID:SCR_002798) was used for statistical analysis, and *p*-values <0.05 were considered statistically significant.

All data generated or analyzed during this study are included in the manuscript and supporting files.

## Figures and Tables

**Appendix 1—figure 1.**
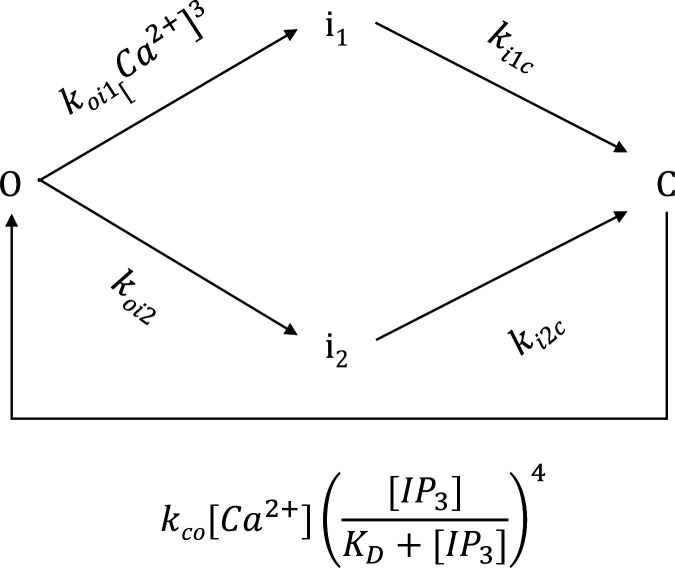
Model describing the dynamics of a cluster of IP_3_Rs. From Ornelas-Guevara et al., 2023. Ca²^+^ diffusion is modeled as a stochastic jump between adjacent compartments (Kraus et al., 1996). The propensity of a Ca²^+^ ion to move depends on the deterministic diffusion coefficient (100  µm²/s) and concentration gradients, assuming isotropic and homogeneous diffusion. All reaction and diffusion events are simulated using the Gillespie algorithm. At each step, an event is selected probabilistically based on its propensity (See propensity functions in Appendix 1—table 1).

**Appendix 2—figure 1.**
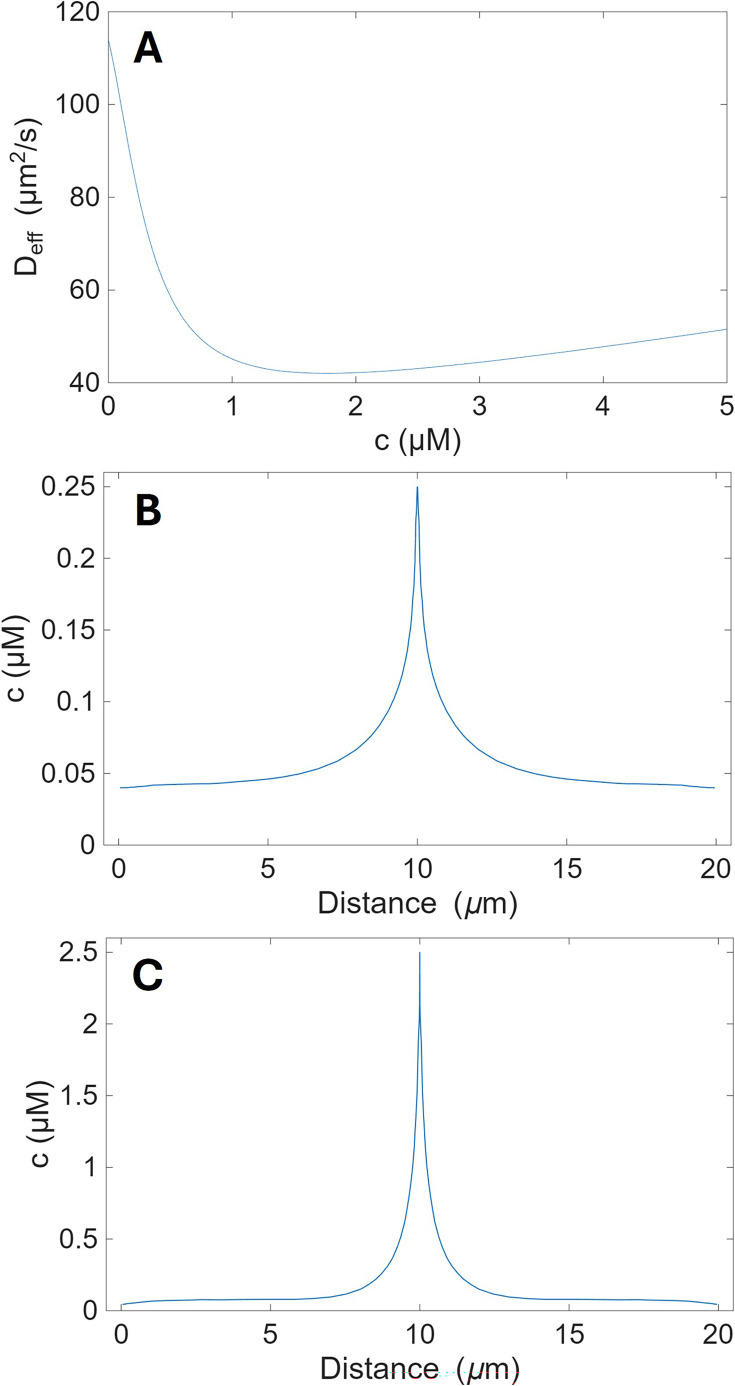
Dependence of the effective Ca2+ diffusion coefficient on [Ca2+] in thepresence of cytosolic buffers and impact on [Ca2+] profiles. (**A**) Effective diffusion coefficient of Ca^2+^ (D_eff_) as a function of free cytosolic [Ca^2+^] (**c**) for a mixture of three buffers. Buffer 1 is immobile and low-affinity (K_d_ = 10 µM, total concentration 15 µM, diffusion coefficient 0 µm²/s). Buffer 2 is mobile and has moderate affinity (K_d_ = 10 µM, total concentration 85 µM, diffusion coefficient 15 µm²/s). Buffer 3 is highly mobile and has a high affinity (K_d_ = 0.1 µM, total concentration 15 µM, diffusion coefficient 120 µm^2^/s). (**B, C**) Spatial profiles of free [Ca^2+^] 10 ms after a constant point-source release in the centre of a 10×10 µm^2^ domain representing a cell, obtained from a two-dimensional reaction–diffusion simulation with explicit Ca^2+^–buffer binding using the same buffer parameters as in (**A**). Only Ca^2+^ release and binding to buffers were included in the simulation. The rate of Ca^2+^ release is 10 times larger in C than in B. [Ca^2+^] peaks at 0.25 µM (**B**) and 2.5 µM (**C**).

**Appendix 3—figure 1.**
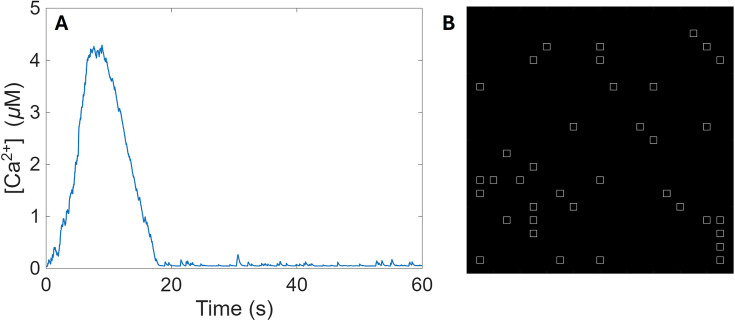
Global Ca²^+^ response at higher [IP_3_]. (**A**) Time course of the averaged cytosolic [Ca²^+^] obtained from the stochastic IP_3_R cluster model for a uniform [IP_3_]=0.1 µM and an effective diffusion coefficient $D_{\mathrm{eff}}=40\Micro m^{2}/s$. All other parameters are identical to those used in the simulations shown in Figure 5. Under these conditions, as opposed to CCRICs, the model produces a ‘classical’ global Ca²^+^ response with larger amplitude and long duration. (**B**) 2D cell geometry (10×10 µm²) used in the simulations. Squares indicate the random positions of IP₃R clusters.

## Data Availability

All data generated or analyzed during this study are included in the manuscript and its supporting files.

## Additional files

MDAR checklist

## Appendix 1

### Description of the computational model

Following previous modeling studies of Ca^2+^ dynamics (Voorsluijs et al., 2019; Ornelas-Guevara et al., 2023), we implement a fully stochastic model of intracellular Ca^2+^ dynamics using the Gillespie algorithm. The model captures IP_3_-induced Ca^2+^ release from IP_3_R clusters, SERCA-mediated reuptake, ER Ca^2+^ leak, and cytosolic Ca^2+^ diffusion. The simulated HeLa cell is represented as a 10×10  µm^2^ square, discretized into a 20×20 grid. Each compartment (0.5×0.5  µm^2^) corresponds to a cytosolic volume of 10^-16 ^l.

Each IP_3_R cluster is represented as a single unit with four discrete states: Open (**O**), Closed (**C**), and two inhibited states (**i₁** and **i₂**). The transition from **C** to **O** depends on both [Ca^2+^] and [IP_3_], while the transition from **O** to **i1** depends on [Ca^2+^], following the model described in Ornelas-Guevara et al., 2023 (see Figure 1). Various spatial distributions of the IP_3_R clusters are investigated in Figure 5A-C, where the locations of the clusters are indicated by white boxes. SERCA activity and ER Ca^2+^ leak are present in all compartments.

**Appendix 1—table 1.** Propensity functions and action of each process. $N_{ca,i}$ and $N_{ca,j}$ represent the number of ions in the current box (i) and in an adjacent box (j) selected randomly.

Process	Propensity function	Action
1. Ca^2+^ diffusion	$4\frac{D_{C}}{\Delta x^{2}}N_{ca,i}$	$N_{ca,i}- 1$ $N_{ca,j}+1$
2. SERCA pumps	$\Omega \frac{v_{p}N_{ca,i}\left (N_{ca,i}- 1\right)}{\left (K_{p}\Omega \right)^{2}+N_{ca,i}\left (N_{ca,i}- 1\right)}$	$N_{ca,i}- 1$
3. Leak from the ER	$\Omega \, \frac{v_p\left[{Ca}^{2+}\right]_b^{2}} {K_p^{2}+\left[{Ca}^{2+}\right]_b^{2}}$	$N_{ca,i}+1$
4. $C\rightarrow O$	$k_{co}\frac{N_{ca,i}}{\Omega }\left (\frac{\left [IP_{3}\right ]}{\left [IP_{3}\right ]+K_{D}}\right)^{4}\left (N_{Vc}- N_{o,i}- N_{i1,i}- N_{i2,i}\right)$	$N_{o,i}=1$ $N_{c,i}=0$
5. $O\rightarrow i_{1}$	$k_{oi1}N_{o,i}\frac{N_{ca,i}\left (N_{ca,i}- 1\right)\left (N_{ca,i}- 2\right)}{\Omega ^{3}}$	$N_{o,i}=0$ $N_{i1,i}=1$
6. $O\rightarrow i_{2}$	$k_{oi1}N_{o,i}$	$N_{o,i}=0$ $N_{i2,i}=1$
7. $i_{1}\rightarrow C$	$k_{i1c}N_{i1,i}$	$N_{i1,i}=0$ $N_{c,i}=1$
8. $i_{2}\rightarrow C$	$k_{i2c}N_{i2,i}$	$N_{i2,i}=0$ $N_{c,i}=1$
9. Ca^2+^ release	$\Omega \Sigma \frac{N_{o,i}}{N_{Vc}}$	$N_{ca,i}+1$

We use an extensivity parameter Ω to convert molecular counts to µM concentrations:

$$\displaystyle \Omega =N_{A}V_{C}\times 10^{- 16}L\mu mol^{- 1}$$

where $N_{A}$ is Avogadro’s number and $V_{C}$ is the volume of each compartment.

**Appendix 1—table 2.** Parameter values used for the simulations shown in Figure 5.

$k_{co}$	$12.5\mu M^{- 1}s^{- 1}$	$C\rightarrow O$
$k_{oi1}$	$0.0125\mu M^{- 3}s^{- 1}$	$O\rightarrow i_{1}$
$k_{oi2}$	$10s^{- 1}$	$O\rightarrow i_{2}$
$k_{i1c}$	$0.00125s^{- 1}$	$i_{1}\rightarrow C$
$k_{i2c}$	$0.5s^{- 1}$	$i_{2}\rightarrow C$
$v_{p}$	$0.225\mu Ms^{- 1}$	Maximal rate of SERCA
$K_{p}$	$0.1\mu M$	SERCA binding constant
$K_{D}$	$0.1\mu M$	Affinity of IP_3_Rs for IP_3_
[*Ca*^2+^]_b_	$0.04\mu M$	Basal cytosolic [Ca^2+^]
$D_{C}$	$100\mu m^{2}s^{- 1}$	Ca^2+^ diffusion coefficient
$\Delta x$	$0.5\mu m$	Length of a compartment
$V_{c}$	$10^{- 16}L$	Volume of a compartment

The pseudocolor maps shown in Figure 5 display the maximum Δ[Ca^2+^]/[Ca^2+^]_b_ reached in each compartment during the simulation. These maps are intended to visualize the spatial distribution of the zones of IP₃R-mediated Ca^2+^ release. The algorithm was implemented in MATLAB R2021b.

## Appendix 2

### Dependency of the effective Ca^2+^ diffusion coefficient on Ca^2+^ concentration

While in pure water the diffusion coefficient of Ca^2+^ is very large (~500 μm^2^s^–1^), in the cytoplasm it was reported to be in the range of 13–65 μm^2^s^–1^, mainly due to interactions with Ca^2+^ buffers (Allbritton et al., 1992). This value was determined experimentally by injecting large amounts of ^45^Ca^2+^ at one end of a test tube filled with cytosolic extracts from *Xenopus* oocytes and measuring the spatial spread of radioactivity with time. Such measurements did not assess the dependency of the diffusion coefficient on the Ca^2+^ concentration. A theoretical expression for the effective diffusion coefficient of Ca^2+^ in the presence of buffers under the fast-buffering approximation was derived by Wagner and Keizer, 1994. This expression was later shown by Smith et al., 1996 to be valid for a large range of buffering conditions. In this framework, the effective diffusion coefficient is given by

$$\displaystyle  D_{eff}=\frac{D_{C}+\sum _{i=1}^{N}D_{B_{i}}\theta _{i}\left (c\right)}{1+\sum _{i=1}^{N}\theta _{i}\left (c\right)}$$

with

$$\displaystyle \theta _{i}\left (c\right)=\frac{B_{T,i}K_{d,i}}{\left (K_{d,i}+c\right)^{2}}$$

Here, $D_{C}$ is the diffusion coefficient of free Ca^2+^, $D_{B_{i}}$ is the diffusion coefficient of buffer species *i*, $B_{T,i}$ is the total concentration of buffer *i*, $K_{d,i}$ is its Ca^2+^-binding affinity, and $c$ denotes the free Ca^2+^ concentration. The dimensionless quantity $\theta _{i}\left (c\right)$ is the buffering capacity of species *i*.

It is worth mentioning that using the fast-buffer approximation is justified because the Ca²^+^–buffer reaction takes place much faster than Ca²^+^ diffuses over the relevant spatial scales. The characteristic reaction relaxation time can be estimated as τ_relax_ ≈ 1/(k_on_·B_tot_ +k_off_), whereas the diffusion timescale over a distance L is t_D_ ≈ L²/D_c_. For representative values (k_on_ = 100 µM⁻¹·s⁻¹, B_tot_ = 15 µM, K_d_ = 10 µM so k_off_ = K_d_·k_on_ = 1000 s⁻¹), τ_relax_ ≈ 1/ (100 µM⁻¹·s⁻¹·15 µM+1000 s⁻¹) ≈ 4×10^–4^ s. Over L=5 µm, t_D_ = 5^2^/D_c_, which for D_c_ = 220 µm²/s gives t_D_ ≈ 0.11 s. Thus, τ_relax_ is ~300 fold shorter than t_D_, so for practical purposes the fast-buffer approximation holds in this regime.

Immobile ($D_{B_{i}}=0$) or slowly diffusing buffers (small $D_{B_{i}}$) slow down the redistribution of Ca^2+^ and, therefore, reduce the effective diffusion coefficient $D_{eff}$ in the Ca^2+^ concentration ranges around and larger than their $K_{d}$. In contrast, fast buffers (large $D_{B_{i}}$) can transport Ca^2+^ away from the source and effectively increase Ca^2+^ mobility. When several buffers with different $K_{d,i}$, $B_{T,i}$, and $D_{B_{i}}$ coexist, their combined effect can make $D_{eff}\left (c\right)$ a non-monotonic function of [Ca^2+^], such that effective Ca^2+^ diffusion coefficient can be larger or smaller than the diffusion coefficient estimated in the experiments of Allbritton et al., 1992.

To illustrate how this mechanism can generate concentration-dependent Ca^2+^ mobility, we consider a simple but physiologically plausible mixture of three buffers with distinct properties chosen to be broadly consistent with well-known cytosolic Ca²^+^-binding proteins (e.g. calbindin-D28k, calmodulin, and parvalbumin; Eisner et al., 2023).

- **Buffer 1**: immobile, low-affinity buffer

$$\displaystyle K_{d,1}=10\mu M, \ \ \ B_{T,1}=15\mu M, \ \ \ D_{B_{1}}=0\mu m^{2}/s$$

- **Buffer 2**: mobile, moderate-affinity buffer

$$\displaystyle K_{d,2}=10\mu M, \ \ \ B_{T,2}=85\mu M, \ \ \ D_{B_{2}}=15\mu m^{2}/s$$

- **Buffer 3**: mobile, high-affinity buffer

$$\displaystyle K_{d,3}=0.1\mu M, \ \ \ B_{T,3}=15\mu M, \ \ \ D_{B_{3}}=120\mu m^{2}/s$$

These parameters are not meant to represent specific molecules, but to span a realistic range: an immobile buffer, a generic mobile Ca^2+^ buffer (with a diffusion coefficient on the order of 10–20 µm^2^/s), and a fast-diffusing Ca^2+^ ligand (with a larger diffusion coefficient and lower concentration, conceptually similar to ADP). Many endogenous Ca^2+^-binding species, including both proteins and small metabolites, fall within or between these regimes and, collectively, can act either as ‘sinks’ that confine Ca^2+^ or as ‘carriers’ that help it spread.

Appendix 2—figure 1 shows $D_{\mathrm{eff}}\left (c\right)$ computed from the expressions given above for this buffer mixture. Because different buffers have different $K_{d}$ and $D_{B}$, the effective diffusion coefficient value varies with $c$ in a non-monotonic way: at some [Ca^2+^] ranges, immobile or slow buffers dominate and reduce Ca^2+^ mobility, whereas at other ranges the contribution of fast mobile carriers is more prominent and increases $D_{\mathrm{eff}}$.

To connect this analysis with spatial [Ca^2+^] profiles, we simulated Ca^2+^ release from a point source in a two-dimensional reaction–diffusion system, including explicit Ca^2+^–buffer binding (i.e. not using the equation to calculate $D_{\mathrm{eff}}$ directly, but the underlying reaction–diffusion equations including Ca^2+^-buffers binding and unbinding with the same parameters as in Appendix 2—figure 1A). We considered two cases: a small-amplitude Ca^2+^ release (Appendix 2—figure 1B) and a large-amplitude Ca^2+^ release (Appendix 2—figure 1C), both with identical buffer parameters and diffusion coefficients. Appendix 2—figure 1B and C show the resulting [Ca^2+^] profiles 10 ms after release.

This example illustrates that in the presence of multiple buffers with different kinetics and mobilities low-amplitude Ca²^+^ elevations can spread further than high-amplitude Ca²^+^ signals. The reason is that low [Ca²^+^] transients are handled mainly by fast, mobile buffers, whereas higher [Ca²^+^] transients increasingly engage slowly diffusing buffers, which then dominate and reduce Ca²^+^ mobility, confining the signal.

Importantly, this is not a universal statement about all possible buffer mixtures, but a specific example showing how realistic combinations of mobile and immobile buffers can produce a situation in which lower-amplitude Ca^2+^ signals have a larger spatial extent than higher-amplitude Ca^2+^ signals.

## Appendix 3

### Global Ca²^+^ responses in the IP_₃_R cluster model at higher stimulation and stronger Ca^2+^ buffering

In the stochastic cluster model used in Figure 5, IP_3_R clusters are represented at discrete spatial sites. Each cluster senses the local Ca²^+^ concentration and its stochastic gating depends on this local [Ca²^+^] and on [IP_3_]. Buffers are not included explicitly. Instead, Ca²^+^ diffusion in the cytosol is described by $D_{\mathrm{eff}}$, which accounts for the combined action of endogenous Ca²^+^-binding species.

In the regime relevant for CCRICs, we use $D_{\mathrm{eff}}=100\Micro m^{2}/s$ and a sub-threshold [IP_3_]=0.07 µM, below the level that produces global Ca²^+^ responses in the model. With these values, the model reproduces key properties of CCRICs: fast kinetics, small amplitude, and large spatial extent.

To simulate the behaviour of the model at higher IP3 stimulation levels, above-threshold IP_3_ concentration, we increased [IP_3_] to 0.1 µM (kept constant in time and space) and used a smaller effective diffusion coefficient, $D_{\mathrm{eff}}=40\Micro m^{2}/s$ expected for the stronger buffering and lower Ca²^+^ mobility with higher-amplitude Ca²^+^ signals (Appendix 2—figure 1).

Under these conditions, the same cluster model generates a global Ca²^+^ response with larger amplitude and longer duration, rather than a loss of activity due to excessive inhibition of the clusters (Appendix 3—figure 1). Thus, within a single modeling framework, low [IP_3_] and $D_{\mathrm{eff}}=100\Micro m^{2}/s$ give rise to CCRIC-like responses, whereas higher [IP_3_] and a more strongly buffered regime ($D_{\mathrm{eff}}=40\Micro m^{2}/s$) produce robust global Ca²^+^ signals, comparable to global responses observed experimentally (Appendix 3—figure 1).

## References

[bib1] Abul-Milh M, Wu Y, Lau B, Lingwood CA, Foster DB (2001). Induction of epithelial cell death including apoptosis by enteropathogenic *Escherichia coli* expressing bundle-forming Pili. Infection and Immunity.

[bib2] Allbritton NL, Meyer T, Stryer L (1992). Range of messenger action of calcium ion and inositol 1,4,5-trisphosphate. Science.

[bib3] Bain C, Keller R, Collington GK, Trabulsi LR, Knutton S (1998). Increased levels of intracellular calcium are not required for the formation of attaching and effacing lesions by enteropathogenic and enterohemorrhagic *Escherichia coli*. Infection and Immunity.

[bib4] Baldwin TJ, Ward W, Aitken A, Knutton S, Williams PH (1991). Elevation of intracellular free calcium levels in HEp-2 cells infected with enteropathogenic *Escherichia coli*. Infection and Immunity.

[bib5] Baldwin TJ, Lee-Delaunay MB, Knutton S, Williams PH (1993). Calcium-calmodulin dependence of actin accretion and lethality in cultured HEp-2 cells infected with enteropathogenic *Escherichia coli*. Infection and Immunity.

[bib6] Baruch K, Gur‐Arie L, Nadler C, Koby S, Yerushalmi G, Ben‐Neriah Y, Yogev O, Shaulian E, Guttman C, Zarivach R, Rosenshine I (2011). Metalloprotease type III effectors that specifically cleave JNK and NF‐κB. The EMBO Journal.

[bib7] Chantret I, Rodolosse A, Barbat A, Dussaulx E, Brot-Laroche E, Zweibaum A, Rousset M (1994). Differential expression of sucrase-isomaltase in clones isolated from early and late passages of the cell line caco-2: Evidence for glucose-dependent negative regulation. Journal of Cell Science.

[bib8] Chatterjee A, Caballero-Franco C, Bakker D, Totten S, Jardim A (2015). Pore-forming activity of the *Escherichia coli* Type III secretion system protein EspD. Journal of Biological Chemistry.

[bib9] Chen HD, Frankel G (2005). Enteropathogenic *Escherichia coli*: unravelling pathogenesis. FEMS Microbiology Reviews.

[bib10] Creasey EA, Delahay RM, Daniell SJ, Frankel G (2003). Yeast two-hybrid system survey of interactions between LEE-encoded proteins of enteropathogenic *Escherichia coli*. Microbiology.

[bib11] Croxen MA, Law RJ, Scholz R, Keeney KM, Wlodarska M, Finlay BB (2013). Recent advances in understanding enteric pathogenic *Escherichia coli*. Clinical Microbiology Reviews.

[bib12] Dong X, Shu L, Zhang J, Yang X, Cheng X, Zhao X, Qu W, Zhu Q, Shou Y, Peng G, Sun B, Yi W, Shu Q, Li X (2023). Ogt-mediated O-GlcNAcylation inhibits astrocytes activation through modulating NF-κB signaling pathway. Journal of Neuroinflammation.

[bib13] Edwards LA, Bajaj-Elliott M, Klein NJ, Murch SH, Phillips AD (2011). Bacterial-epithelial contact is a key determinant of host innate immune responses to enteropathogenic and enteroaggregative *Escherichia coli*. PLOS ONE.

[bib14] Eisner D, Neher E, Taschenberger H, Smith G (2023). Physiology of intracellular calcium buffering. Physiological Reviews.

[bib15] Falcke M (2003). Deterministic and stochastic models of intracellular Ca^2+^ waves. New Journal of Physics.

[bib16] Foskett JK, White C, Cheung KH, Mak DOD (2007). Inositol trisphosphate receptor Ca2+ release channels. Physiological Reviews.

[bib17] Foubister V, Rosenshine I, Finlay BB (1994). A diarrheal pathogen, enteropathogenic *Escherichia coli* (EPEC), triggers a flux of inositol phosphates in infected epithelial cells. The Journal of Experimental Medicine.

[bib18] Gao X, Wan F, Mateo K, Callegari E, Wang D, Deng W, Puente J, Li F, Chaussee MS, Finlay BB, Lenardo MJ, Hardwidge PR (2009). Bacterial effector binding to ribosomal protein S3 subverts NF-kappaB function. PLOS Pathogens.

[bib19] Garmendia J, Phillips AD, Carlier MF, Chong Y, Schüller S, Marches O, Dahan S, Oswald E, Shaw RK, Knutton S, Frankel G (2004). TccP is an enterohaemorrhagic *Escherichia coli* O157:H7 type III effector protein that couples Tir to the actin-cytoskeleton. Cellular Microbiology.

[bib20] Gillespie DT (1976). A general method for numerically simulating the stochastic time evolution of coupled chemical reactions. Journal of Computational Physics.

[bib21] Giogha C, Lung TWF, Mühlen S, Pearson JS, Hartland EL (2015). Substrate recognition by the zinc metalloprotease effector NleC from enteropathogenic *Escherichia coli*. Cellular Microbiology.

[bib22] Gomes TAT, Elias WP, Scaletsky ICA, Guth BEC, Rodrigues JF, Piazza RMF, Ferreira LCS, Martinez MB (2016). Diarrheagenic *Escherichia coli*. Brazilian Journal of Microbiology.

[bib23] Guignot J, Segura A, Tran Van Nhieu G (2015). The serine protease EspC from enteropathogenic *Escherichia coli* regulates pore formation and cytotoxicity mediated by the Type III secretion system. PLOS Pathogens.

[bib24] Guignot J, Tran Van Nhieu G (2016). Bacterial control of pores induced by the Type III secretion system: mind the gap. Frontiers in Immunology.

[bib25] Hazen TH, Donnenberg MS, Panchalingam S, Antonio M, Hossain A, Mandomando I, Ochieng JB, Ramamurthy T, Tamboura B, Qureshi S, Quadri F, Zaidi A, Kotloff KL, Levine MM, Barry EM, Kaper JB, Rasko DA, Nataro JP (2016). Genomic diversity of EPEC associated with clinical presentations of differing severity. Nature Microbiology.

[bib26] Kraus M, Wolf B, Wolf B (1996). Crosstalk between cellular morphology and calcium oscillation patterns Insights from a stochastic computer model. Cell Calcium.

[bib27] Levine MM, Nataro JP, Karch H, Baldini MM, Kaper JB, Black RE, Clements ML, O’Brien AD (1985). The diarrheal response of humans to some classic serotypes of enteropathogenic *Escherichia coli* is dependent on a plasmid encoding an enteroadhesiveness factor. The Journal of Infectious Diseases.

[bib28] Li S, Zhang L, Yao Q, Li L, Dong N, Rong J, Gao W, Ding X, Sun L, Chen X, Chen S, Shao F (2013). Pathogen blocks host death receptor signalling by arginine GlcNAcylation of death domains. Nature.

[bib29] Liu AR, Ramakrishnan P (2021). Regulation of nuclear factor-kappaB function by O-GlcNAcylation in inflammation and cancer. Frontiers in Cell and Developmental Biology.

[bib30] Lozer DM, Souza TB, Monfardini MV, Vicentini F, Kitagawa SS, Scaletsky ICA, Spano LC (2013). Genotypic and phenotypic analysis of diarrheagenic *Escherichia coli* strains isolated from Brazilian children living in low socioeconomic level communities. BMC Infectious Diseases.

[bib31] Mills E, Baruch K, Charpentier X, Kobi S, Rosenshine I (2008). Real-time analysis of effector translocation by the Type III secretion system of enteropathogenic *Escherichia coli*. Cell Host & Microbe.

[bib32] Monjarás Feria J, García-Gómez E, Espinosa N, Minamino T, Namba K, González-Pedrajo B (2012). Role of EscP (Orf16) in injectisome biogenesis and regulation of Type III protein secretion in enteropathogenic *Escherichia coli*. Journal of Bacteriology.

[bib33] Motolani A, Martin M, Wang B, Jiang G, Alipourgivi F, Huang X, Safa A, Liu Y, Lu T (2023). Critical role of Novel O-GlcNAcylation of S550 and S551 on the p65 subunit of NF-κB in pancreatic cancer. Cancers.

[bib34] Ornelas-Guevara R, Gil D, Voorsluijs V, Dupont G (2023). Computational investigation of IP3 diffusion. Scientific Reports.

[bib35] Özcan S, Andrali SS, Cantrell JEL (2010). Modulation of transcription factor function by O-GlcNAc modification. Biochimica et Biophysica Acta (BBA) - Gene Regulatory Mechanisms.

[bib36] Pallett MA, Berger CN, Pearson JS, Hartland EL, Frankel G (2014). The Type III secretion effector NleF of enteropathogenic *Escherichia coli* activates NF-κB early during infection. Infection and Immunity.

[bib37] Pearson JS, Giogha C, Ong SY, Kennedy CL, Kelly M, Robinson KS, Lung TWF, Mansell A, Riedmaier P, Oates CVL, Zaid A, Mühlen S, Crepin VF, Marches O, Ang CS, Williamson NA, O’Reilly LA, Bankovacki A, Nachbur U, Infusini G, Webb AI, Silke J, Strasser A, Frankel G, Hartland EL (2013). A type III effector antagonizes death receptor signalling during bacterial gut infection. Nature.

[bib38] Pearson JS, Giogha C, Wong Fok Lung T, Hartland EL (2016). The genetics of enteropathogenic *Escherichia coli* virulence. Annual Review of Genetics.

[bib39] Ramachandran RP, Spiegel C, Keren Y, Danieli T, Melamed-Book N, Pal RR, Zlotkin-Rivkin E, Rosenshine I, Aroeti B (2020). Mitochondrial targeting of the enteropathogenic *Escherichia coli* MAP triggers calcium mobilization, ADAM10-MAP Kinase signaling, and host cell apoptosis. mBio.

[bib40] Ruan HB, Ma Y, Torres S, Zhang B, Feriod C, Heck RM, Qian K, Fu M, Li X, Nathanson MH, Bennett AM, Nie Y, Ehrlich BE, Yang X (2017). Calcium-dependent O-GlcNAc signaling drives liver autophagy in adaptation to starvation. Genes & Development.

[bib41] Ruchaud-Sparagano MH, Mühlen S, Dean P, Kenny B (2011). The enteropathogenic *E. coli* (EPEC) Tir effector inhibits NF-κB activity by targeting TNFα receptor-associated factors. PLOS Pathogens.

[bib42] Sal-Man N, Deng W, Finlay BB (2012). EscI: A crucial component of the type III secretion system forms the inner rod structure in enteropathogenic *Escherichia coli*. Biochemical Journal.

[bib43] Savio LEB, de Andrade Mello P, da Silva CG, Coutinho-Silva R (2018). The P2X7 receptor in inflammatory diseases: Angel or demon?. Frontiers in Pharmacology.

[bib44] Serapio-Palacios A, Navarro-Garcia F (2016). EspC, an autotransporter protein secreted by enteropathogenic *Escherichia coli*, causes apoptosis and necrosis through caspase and calpain activation, including direct procaspase-3 cleavage. mBio.

[bib45] Smedler E, Uhlén P (2014). Frequency decoding of calcium oscillations. Biochimica et Biophysica Acta.

[bib46] Smith GD, Wagner J, Keizer J (1996). Validity of the rapid buffering approximation near a point source of calcium ions. Biophysical Journal.

[bib47] Swillens S, Dupont G, Combettes L, Champeil P (1999). From calcium blips to calcium puffs: theoretical analysis of the requirements for interchannel communication. PNAS.

[bib48] Thomas D, Lipp P, Tovey SC, Berridge MJ, Li W, Tsien RY, Bootman MD (2000). Microscopic properties of elementary Ca2+ release sites in non-excitable cells. Current Biology.

[bib49] Tran Van Nhieu G, Kai Liu B, Zhang J, Pierre F, Prigent S, Sansonetti P, Erneux C, Kuk Kim J, Suh PG, Dupont G, Combettes L (2013). Actin-based confinement of calcium responses during Shigella invasion. Nature Communications.

[bib50] Vandeput F, Combettes L, Mills SJ, Backers K, Wohlkönig A, Parys JB, De Smedt H, Missiaen L, Dupont G, Potter BVL, Erneux C (2007). Biphenyl 2,3′,4,5′,6‐pentakisphosphate, a novel inositol polyphosphate surrogate, modulates Ca ^2+^ responses in rat hepatocytes. The FASEB Journal.

[bib51] Voorsluijs V, Dawson SP, De Decker Y, Dupont G (2019). Deterministic limit of intracellular calcium spikes. Physical Review Letters.

[bib52] Wagner J, Keizer J (1994). Effects of rapid buffers on Ca2+ diffusion and Ca2+ oscillations. Biophysical Journal.

[bib53] Yan D, Quan H, Wang L, Liu F, Liu H, Chen J, Cao X, Ge B (2013). Enteropathogenic *Escherichia coli* Tir recruits cellular SHP-2 through ITIM motifs to suppress host immune response. Cellular Signalling.

[bib54] Yen H, Ooka T, Iguchi A, Hayashi T, Sugimoto N, Tobe T (2010). NleC, a Type III secretion protease, compromises NF-κB activation by targeting p65/RelA. PLOS Pathogens.

[bib55] Zhang L, Ding X, Cui J, Xu H, Chen J, Gong YN, Hu L, Zhou Y, Ge J, Lu Q, Liu L, Chen S, Shao F (2012). Cysteine methylation disrupts ubiquitin-chain sensing in NF-κB activation. Nature.

[bib56] Zhong Q, Roumeliotis TI, Kozik Z, Cepeda-Molero M, Fernández LÁ, Shenoy AR, Bakal C, Frankel G, Choudhary JS (2020). Clustering of Tir during enteropathogenic *E. coli* infection triggers calcium influx-dependent pyroptosis in intestinal epithelial cells. PLOS Biology.

[bib57] Zhong Q, Chatterjee S, Choudhary JS, Frankel G (2022). EPEC‐induced activation of the Ca ^2+^ transporter TRPV2 leads to pyroptotic cell death. Molecular Microbiology.

