# Supplementary figures and images for: Enteropathogenic Escherichia coli-mediated fast and coordinated Ca²+ responses regulate NF-κB activation
Source: eLife. 2026 Jul 22;14:RP108953. doi: 10.7554/eLife.108953 (PMC13391085; doi:10.7554/eLife.108953)

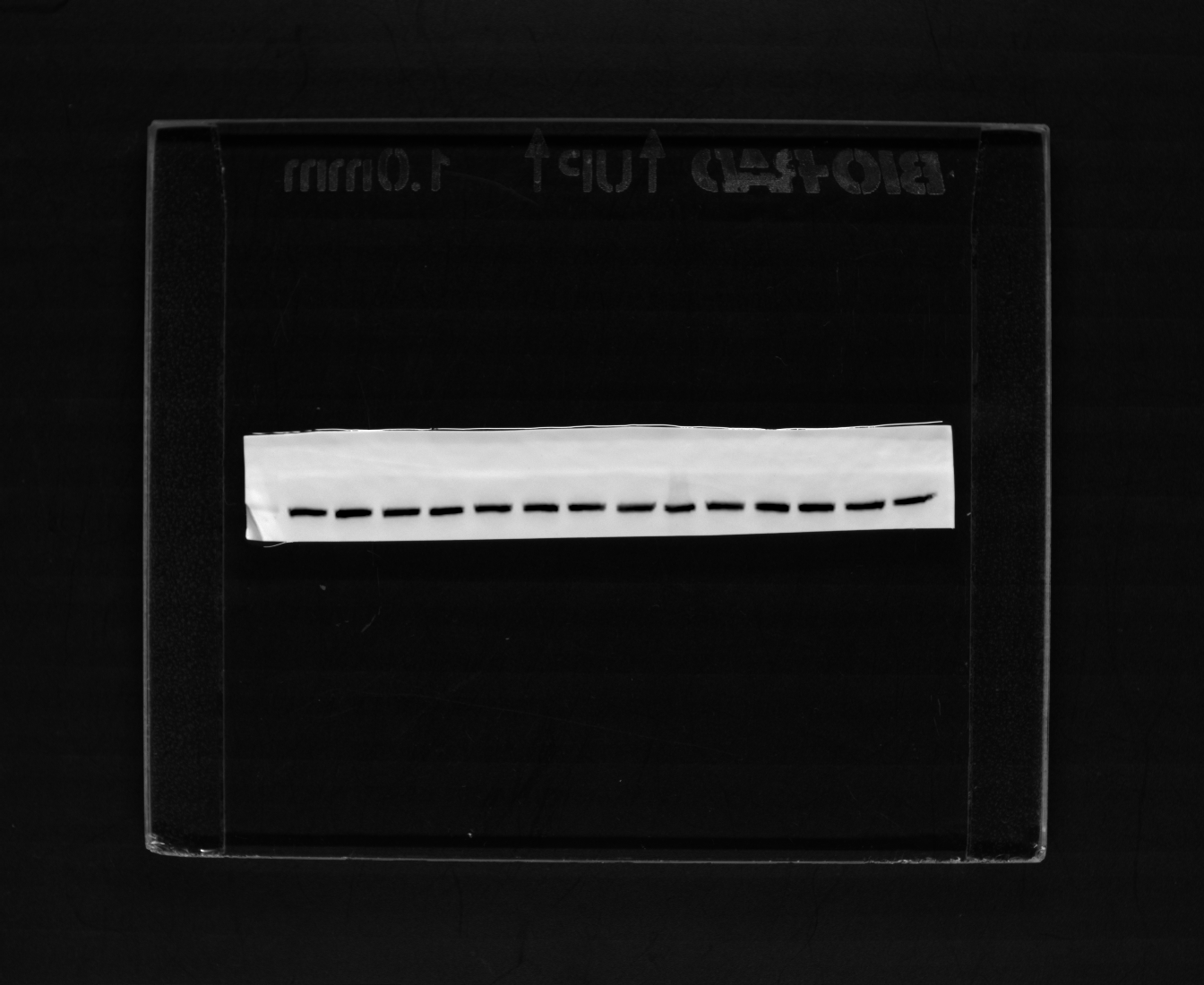

Supplement: Figure 6—source data 2. [file elife-108953-fig6-data2.zip › Figure 6-Source Data 2/Figure 6 A/Fig6A HSP90.tif]

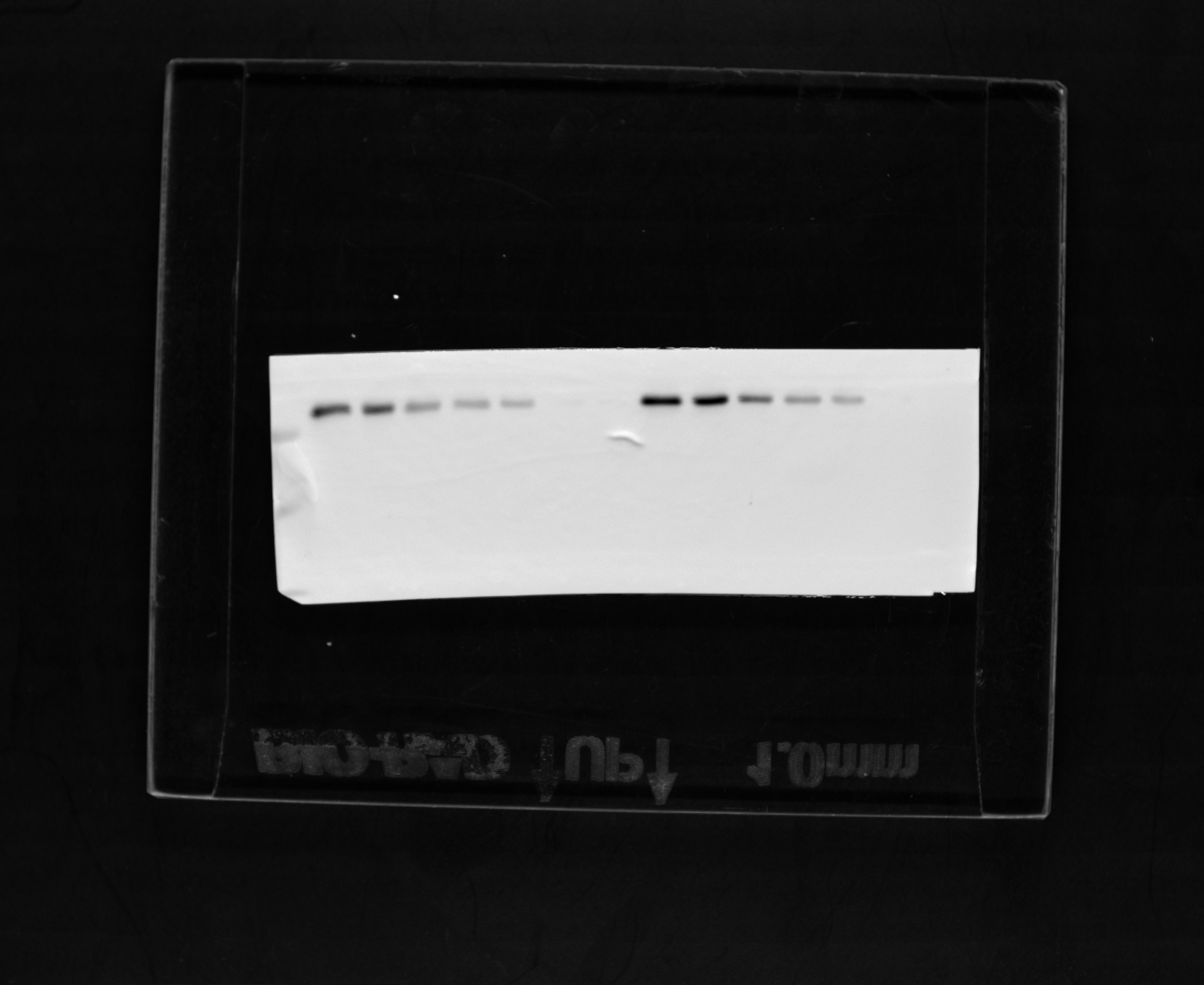

Supplement: Figure 6—source data 2. [file elife-108953-fig6-data2.zip › Figure 6-Source Data 2/Figure 6 A/Fig6A IKBa.tif]

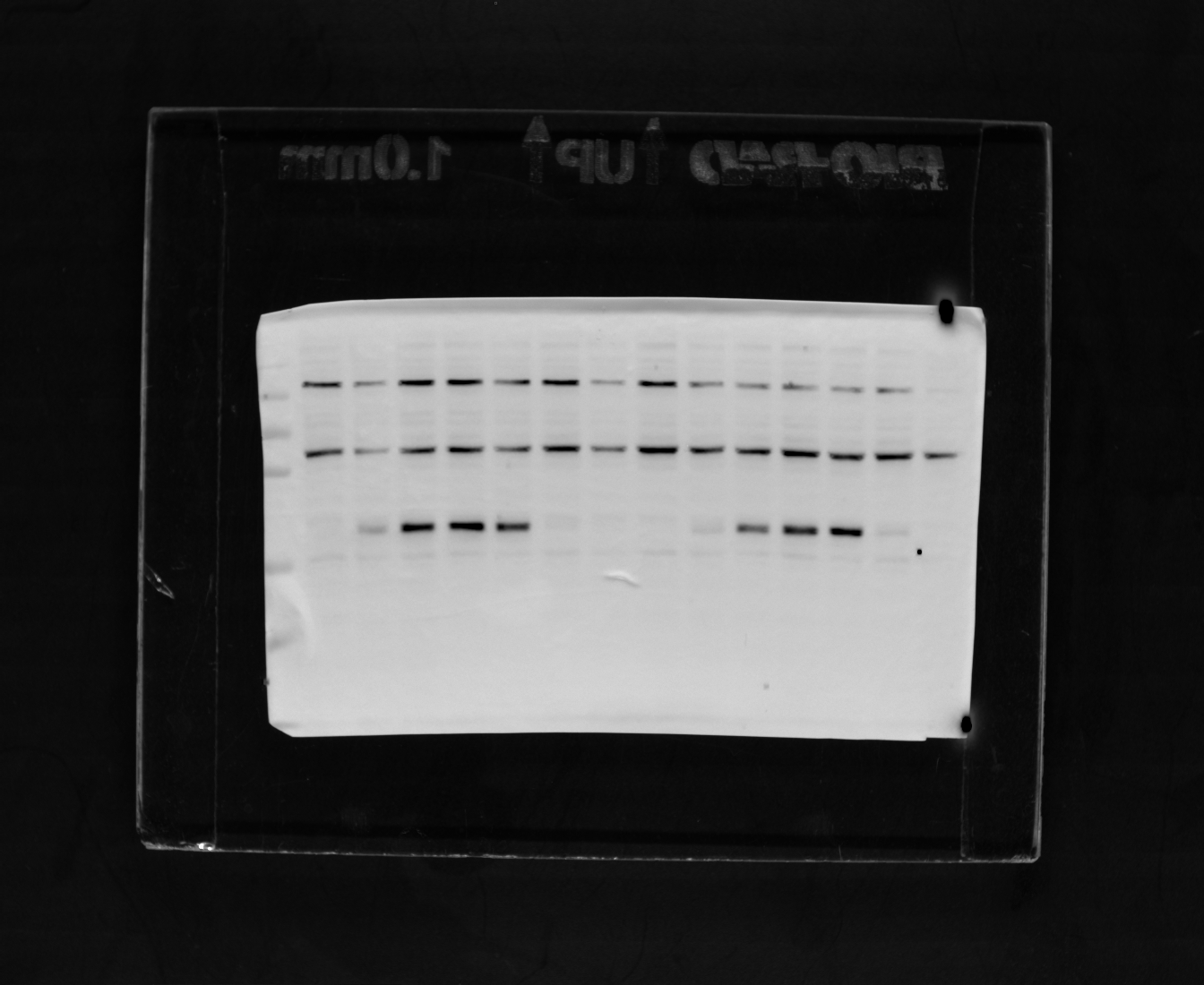

Supplement: Figure 6—source data 2. [file elife-108953-fig6-data2.zip › Figure 6-Source Data 2/Figure 6 A/Fig6A p-IKBa.tif]

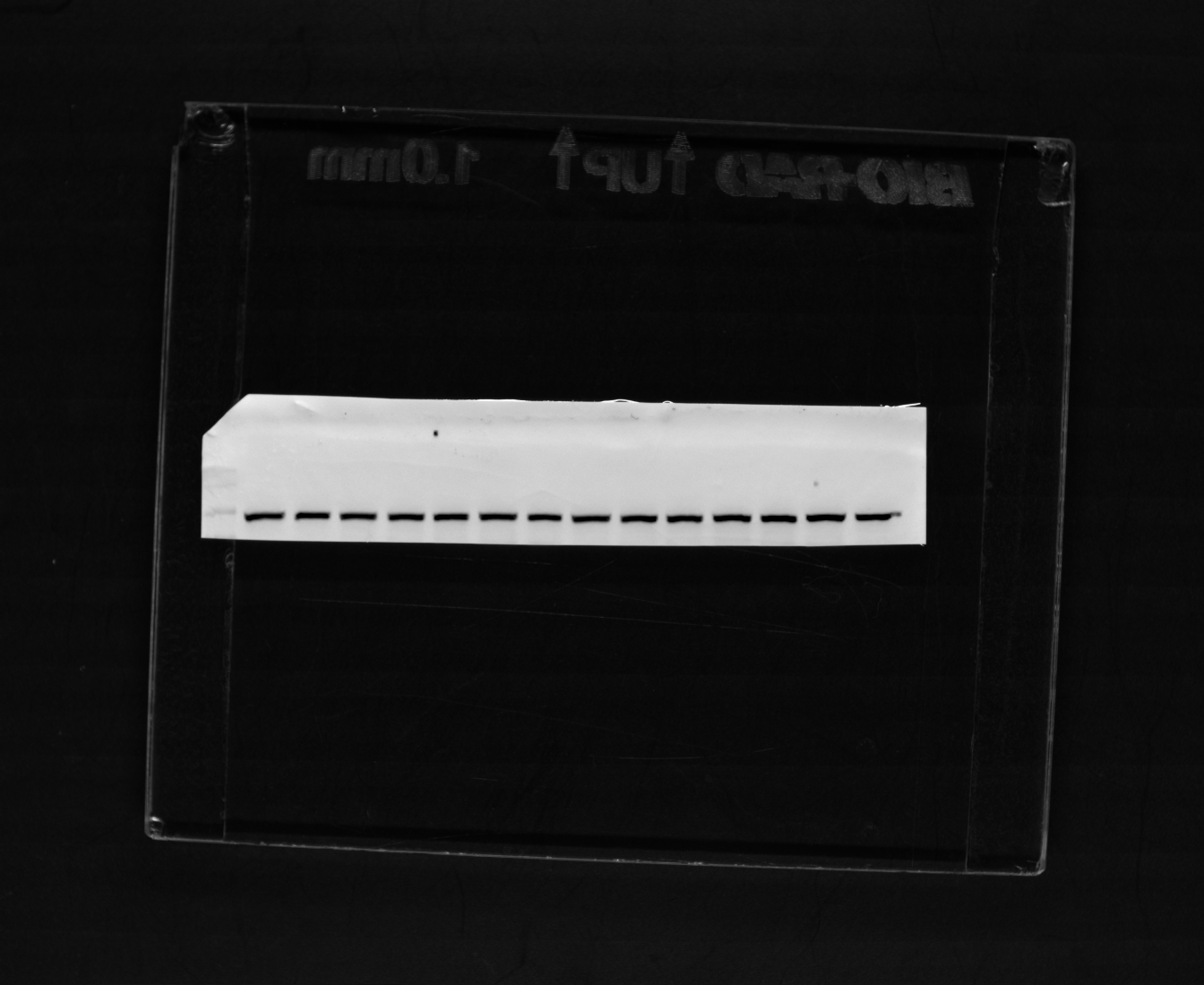

Supplement: Figure 6—source data 2. [file elife-108953-fig6-data2.zip › Figure 6-Source Data 2/Figure 6 D/Fig6D HSP90.tif]

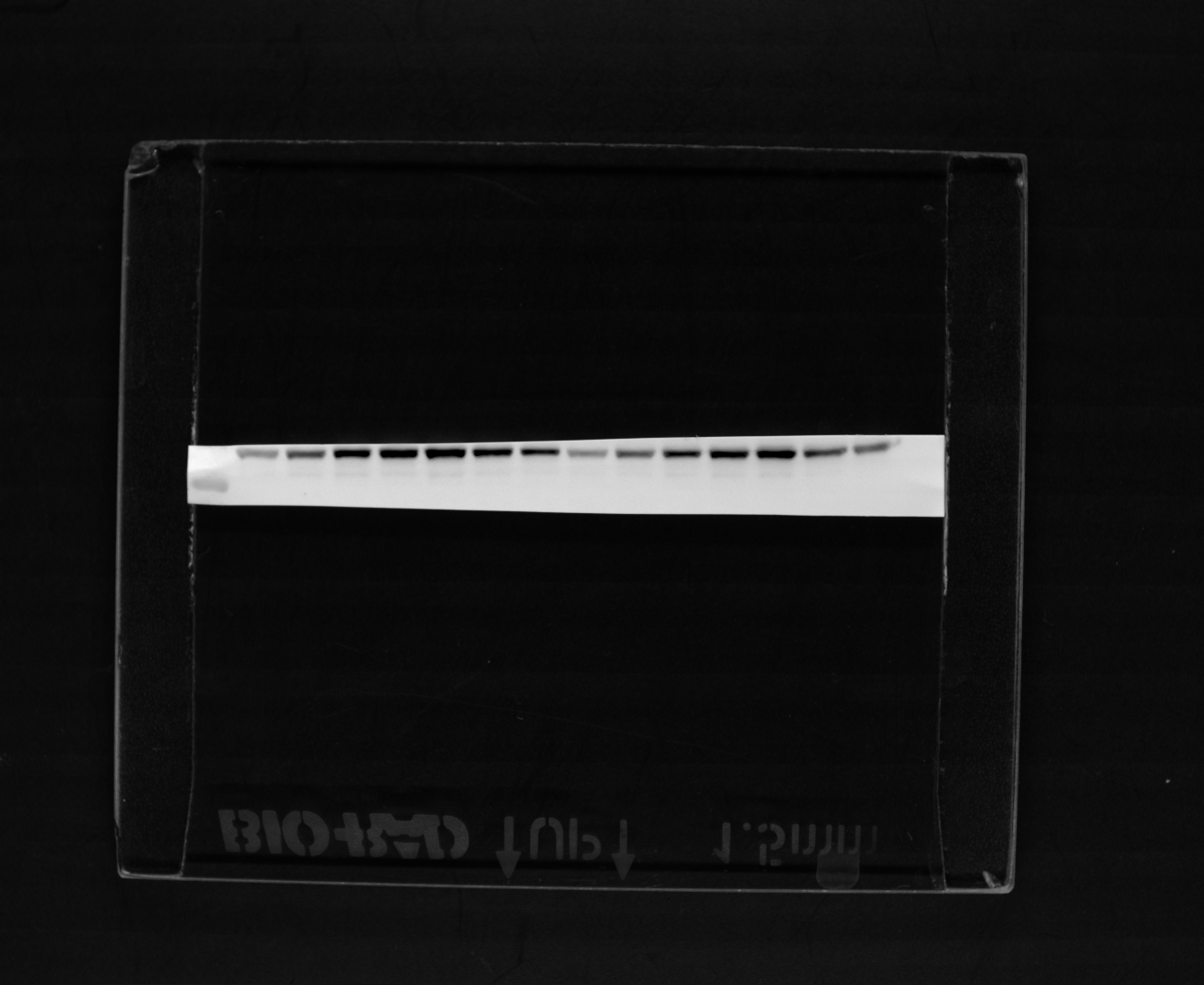

Supplement: Figure 6—source data 2. [file elife-108953-fig6-data2.zip › Figure 6-Source Data 2/Figure 6 D/Fig6D p-p65.tif]

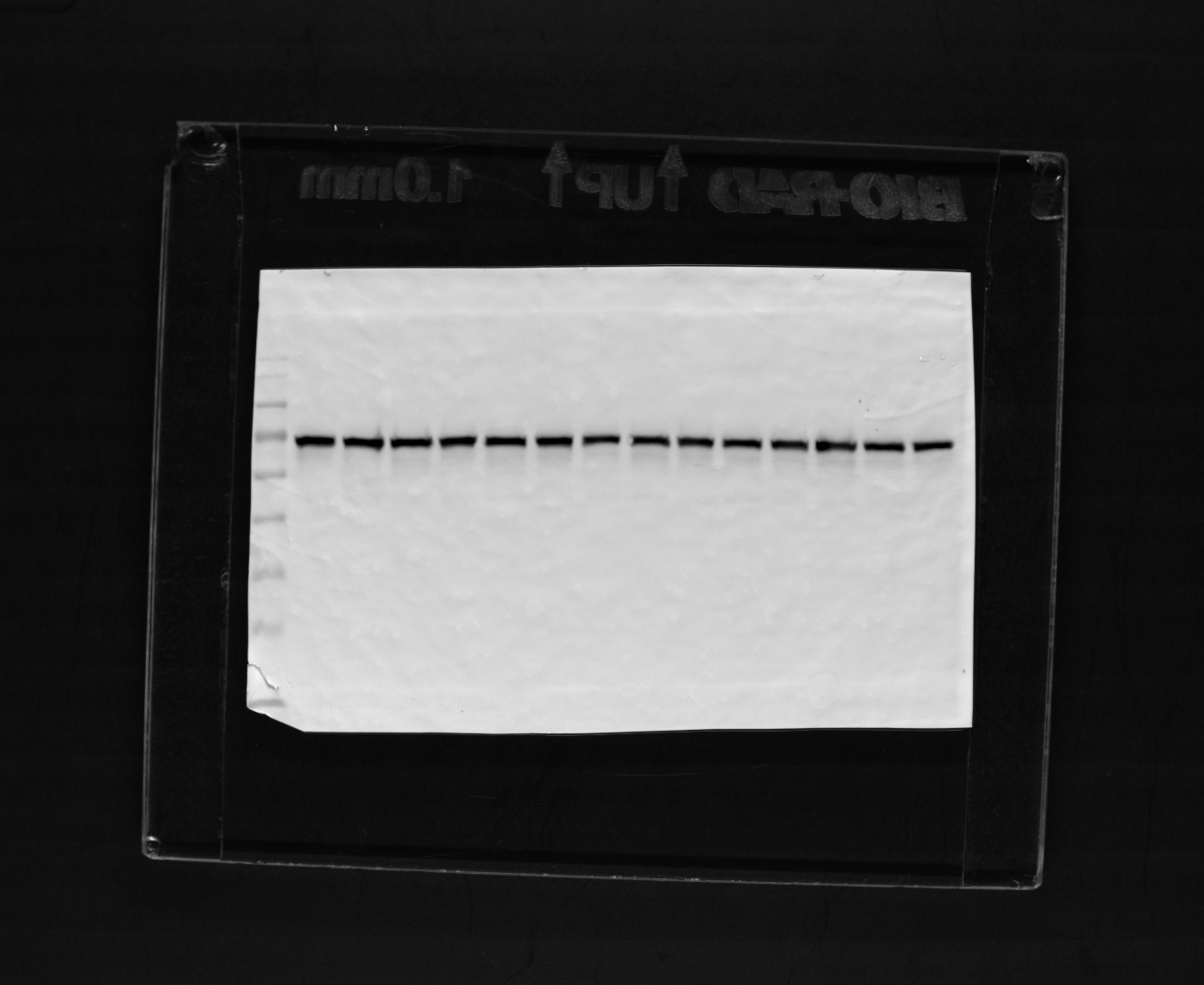

Supplement: Figure 6—source data 2. [file elife-108953-fig6-data2.zip › Figure 6-Source Data 2/Figure 6 D/Fig6D p65.tif]

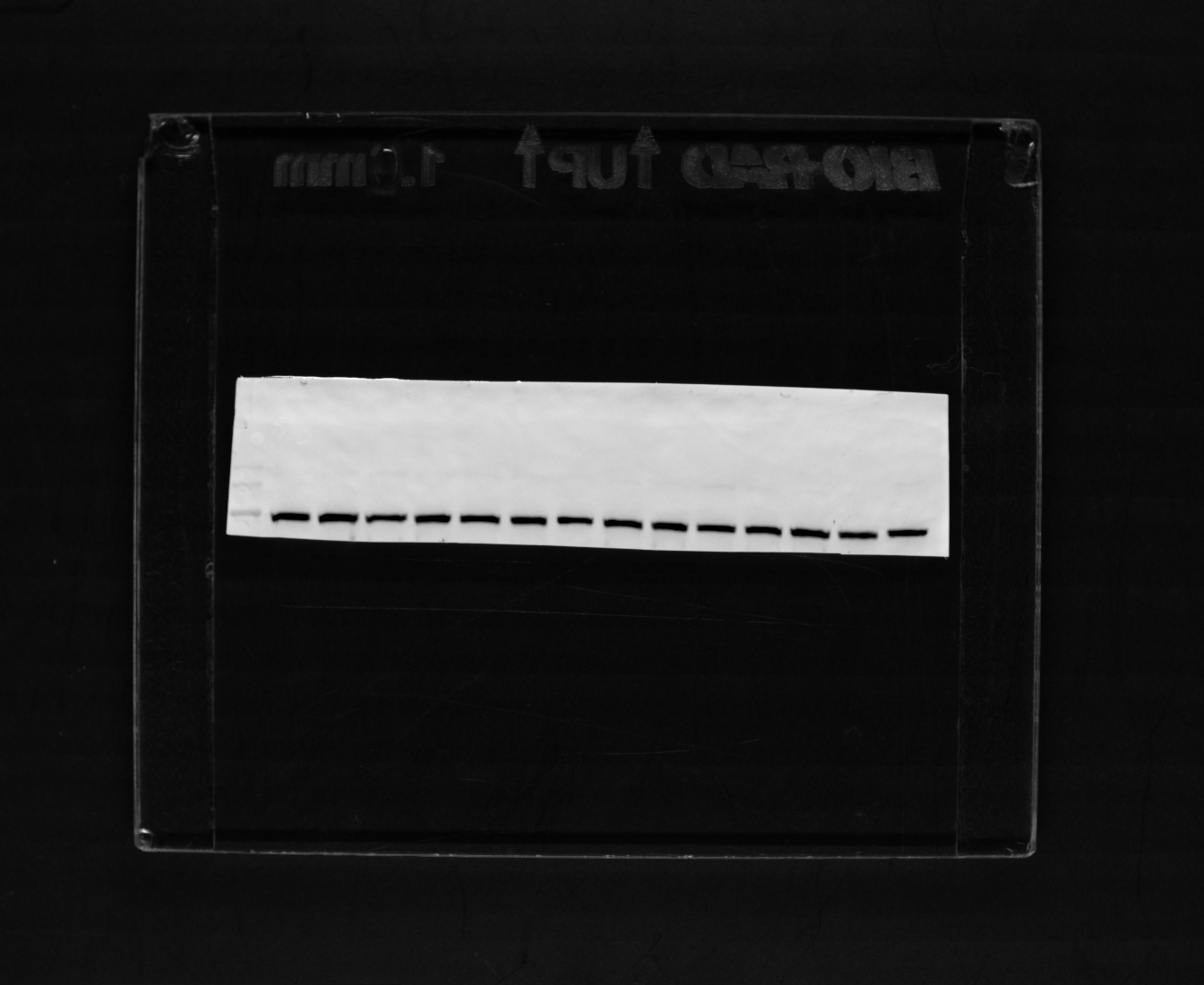

Supplement: Figure 6—source data 2. [file elife-108953-fig6-data2.zip › Figure 6-Source Data 2/Figure 6 F/Fig6F HSP90.tif]

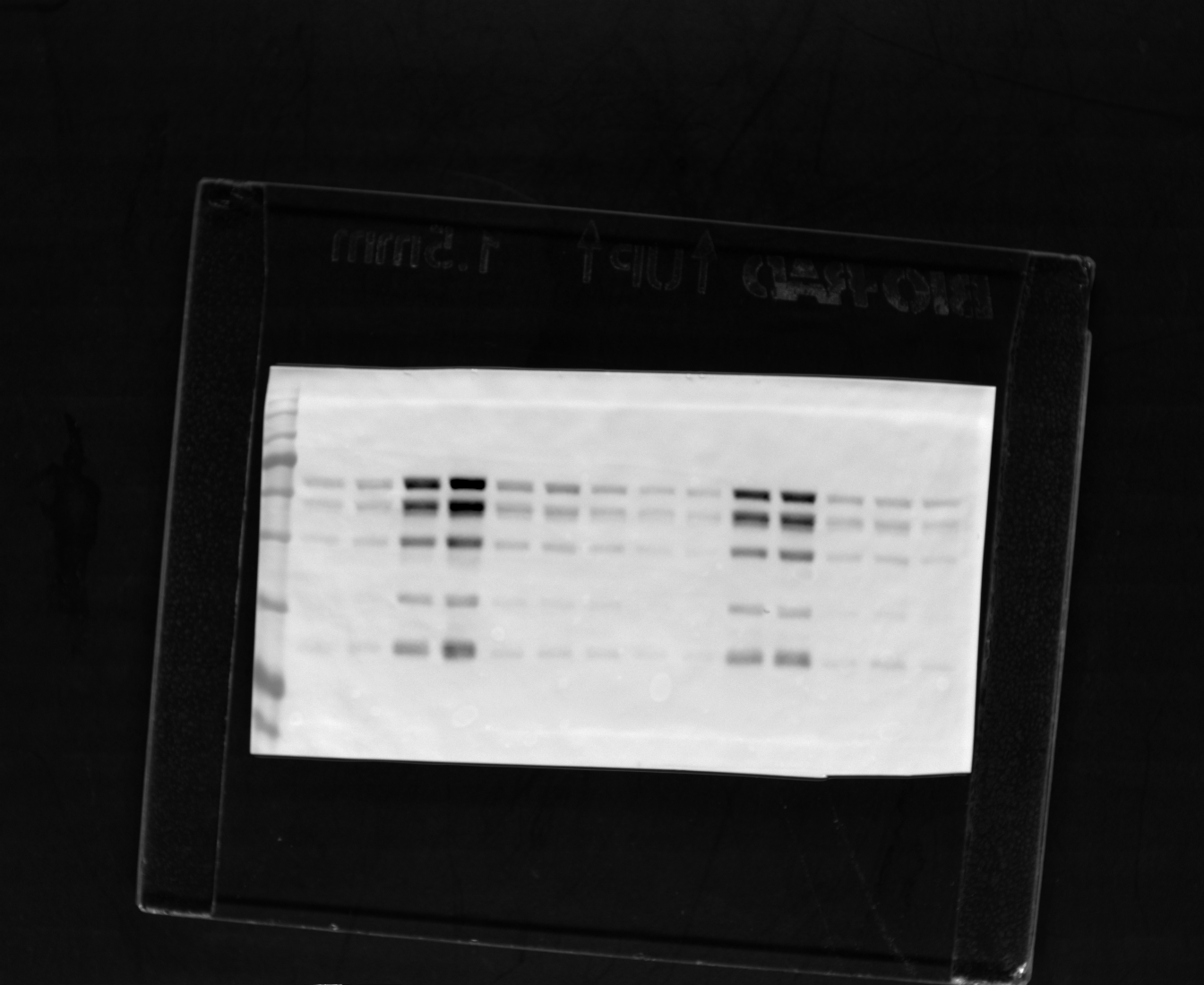

Supplement: Figure 6—source data 2. [file elife-108953-fig6-data2.zip › Figure 6-Source Data 2/Figure 6 F/Fig6F p-p65.tif]

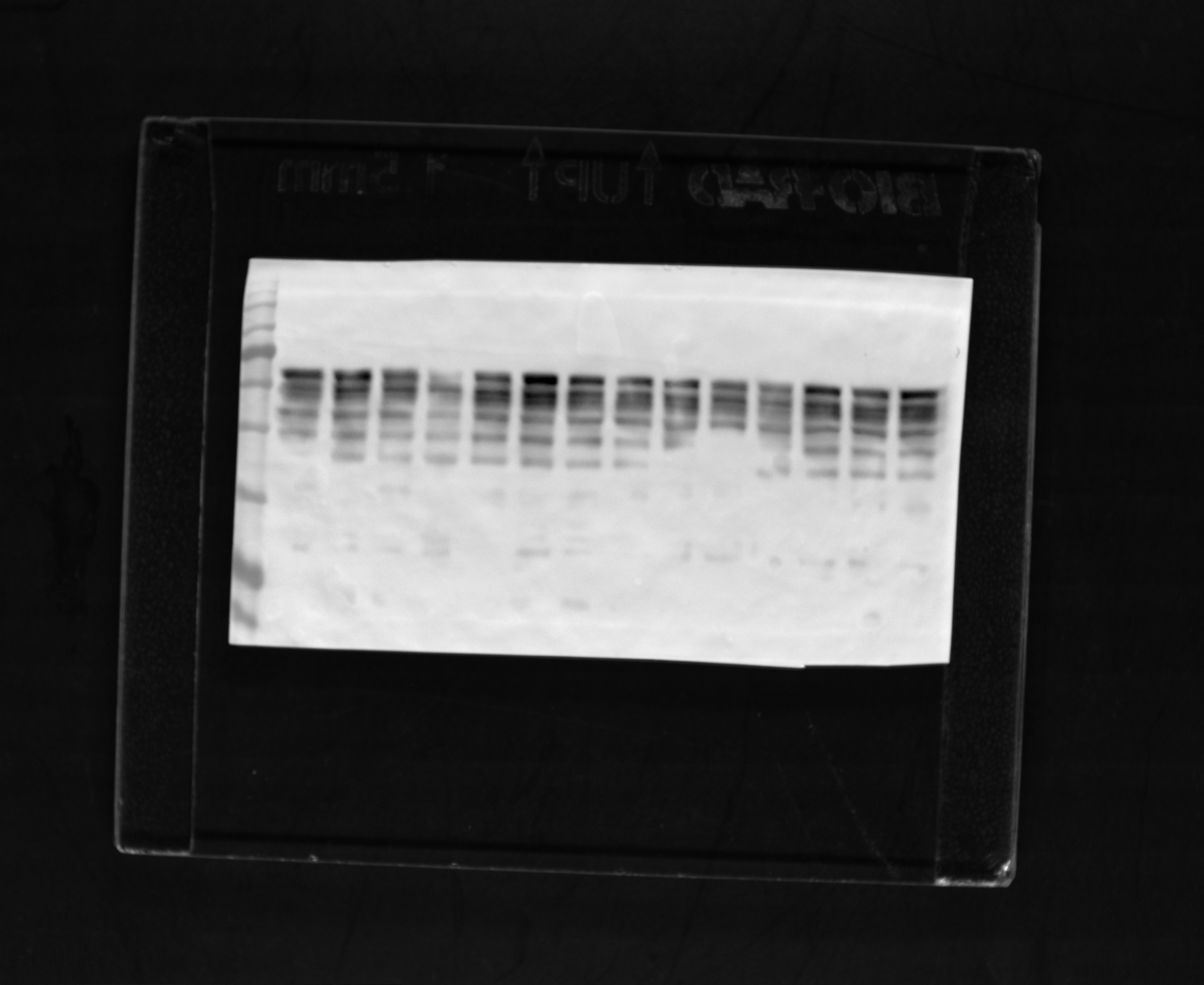

Supplement: Figure 6—source data 2. [file elife-108953-fig6-data2.zip › Figure 6-Source Data 2/Figure 6 F/Fig6F p65.tif]

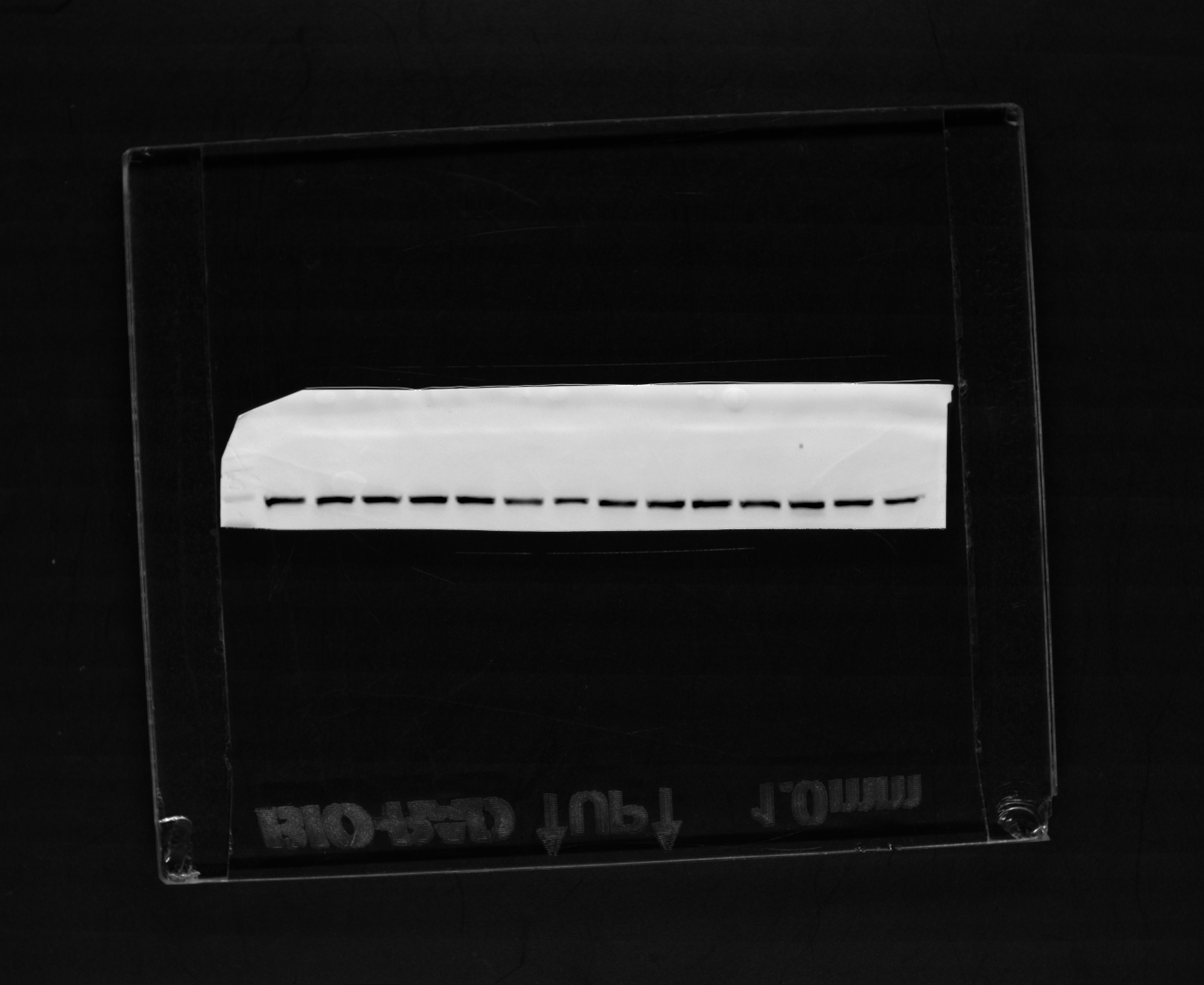

Supplement: Figure 6—source data 2. [file elife-108953-fig6-data2.zip › Figure 6-Source Data 2/Figure 6 G/Fig6G HSP90.tif]

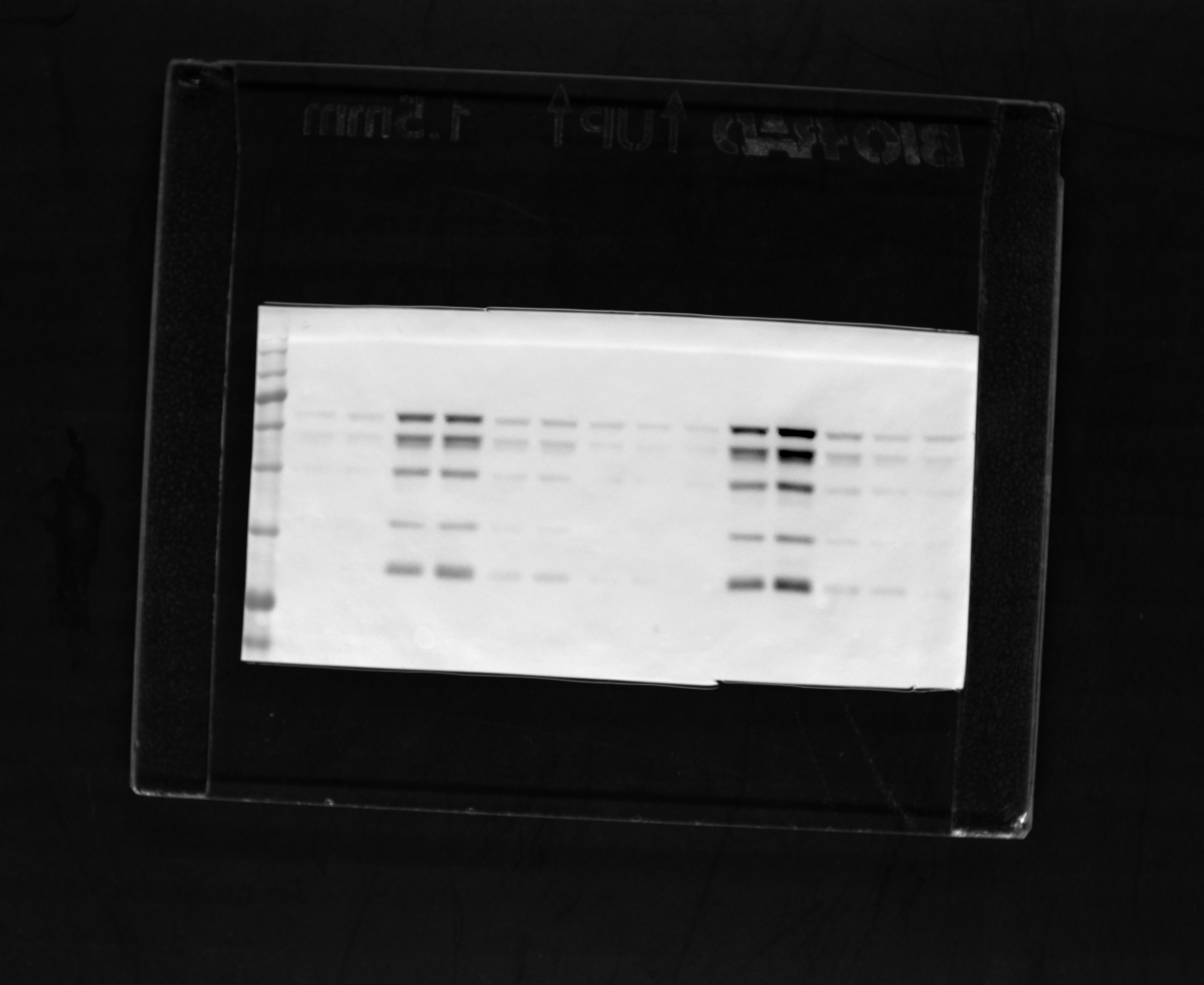

Supplement: Figure 6—source data 2. [file elife-108953-fig6-data2.zip › Figure 6-Source Data 2/Figure 6 G/Fig6G p-p65.tif]

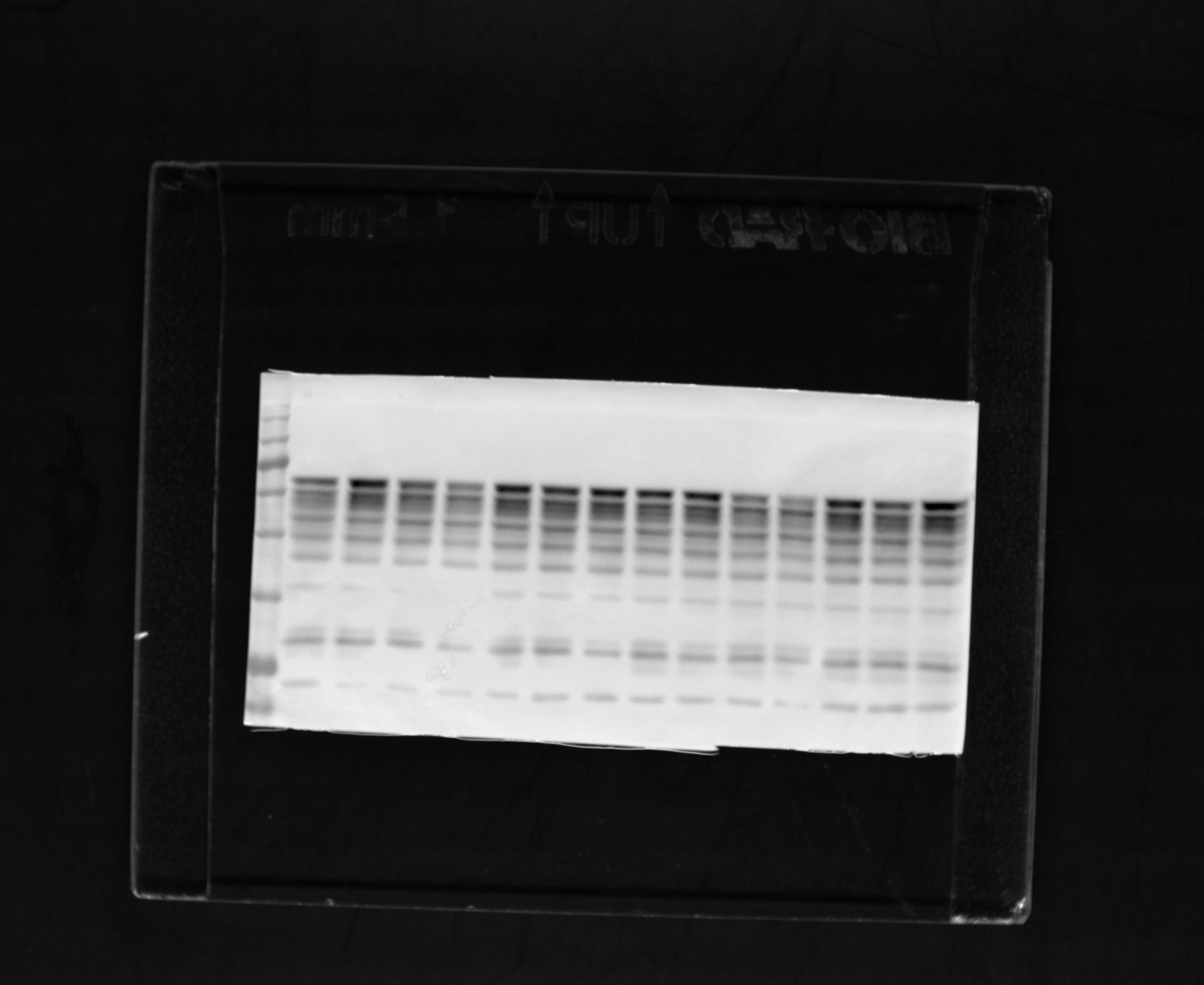

Supplement: Figure 6—source data 2. [file elife-108953-fig6-data2.zip › Figure 6-Source Data 2/Figure 6 G/Fig6G p65.tif]

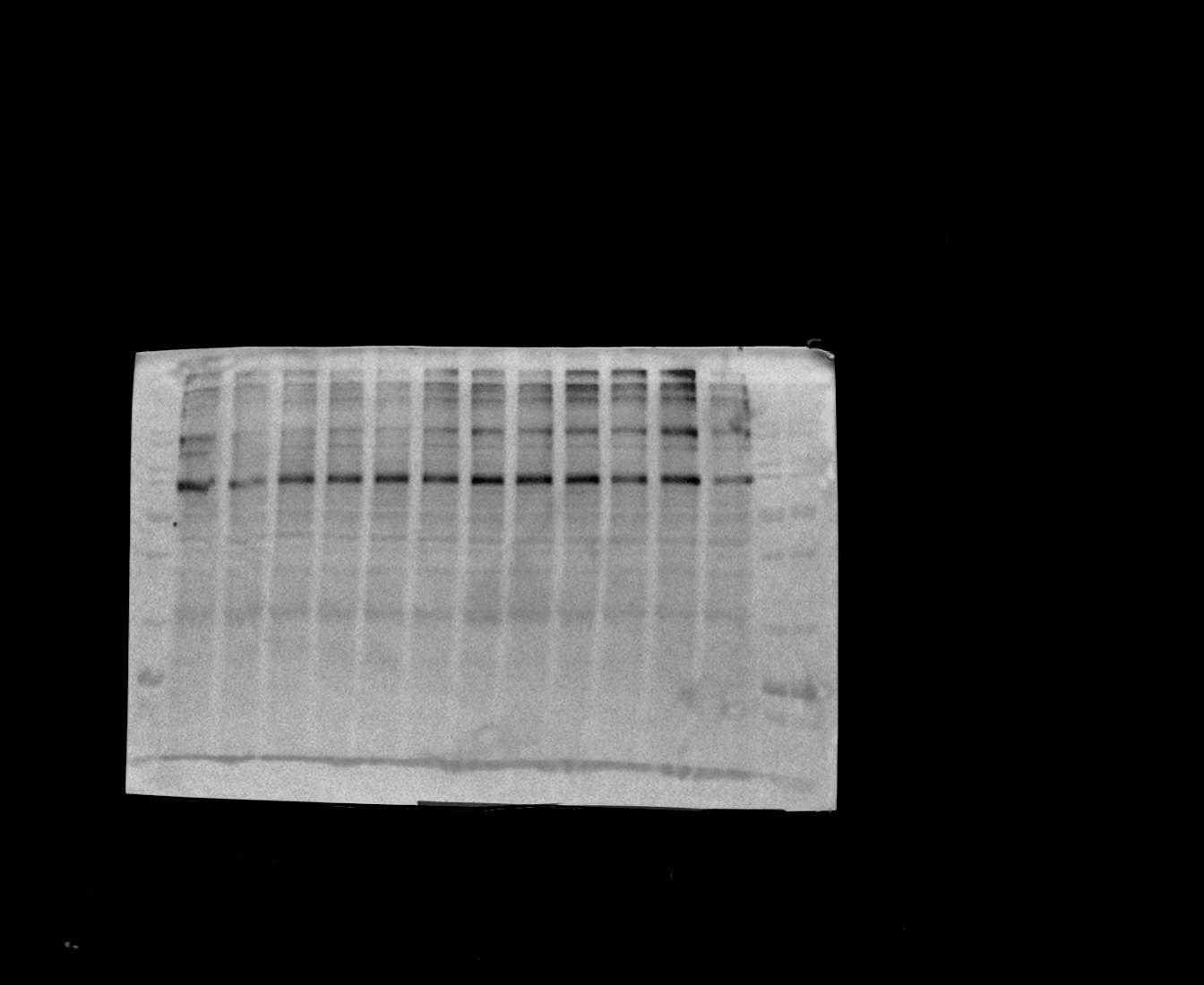

Supplement: Figure 6—figure supplement 1—source data 2. [file elife-108953-fig6-figsupp1-data2.zip › FigS6 Supplement data OGlNac composite.tif]

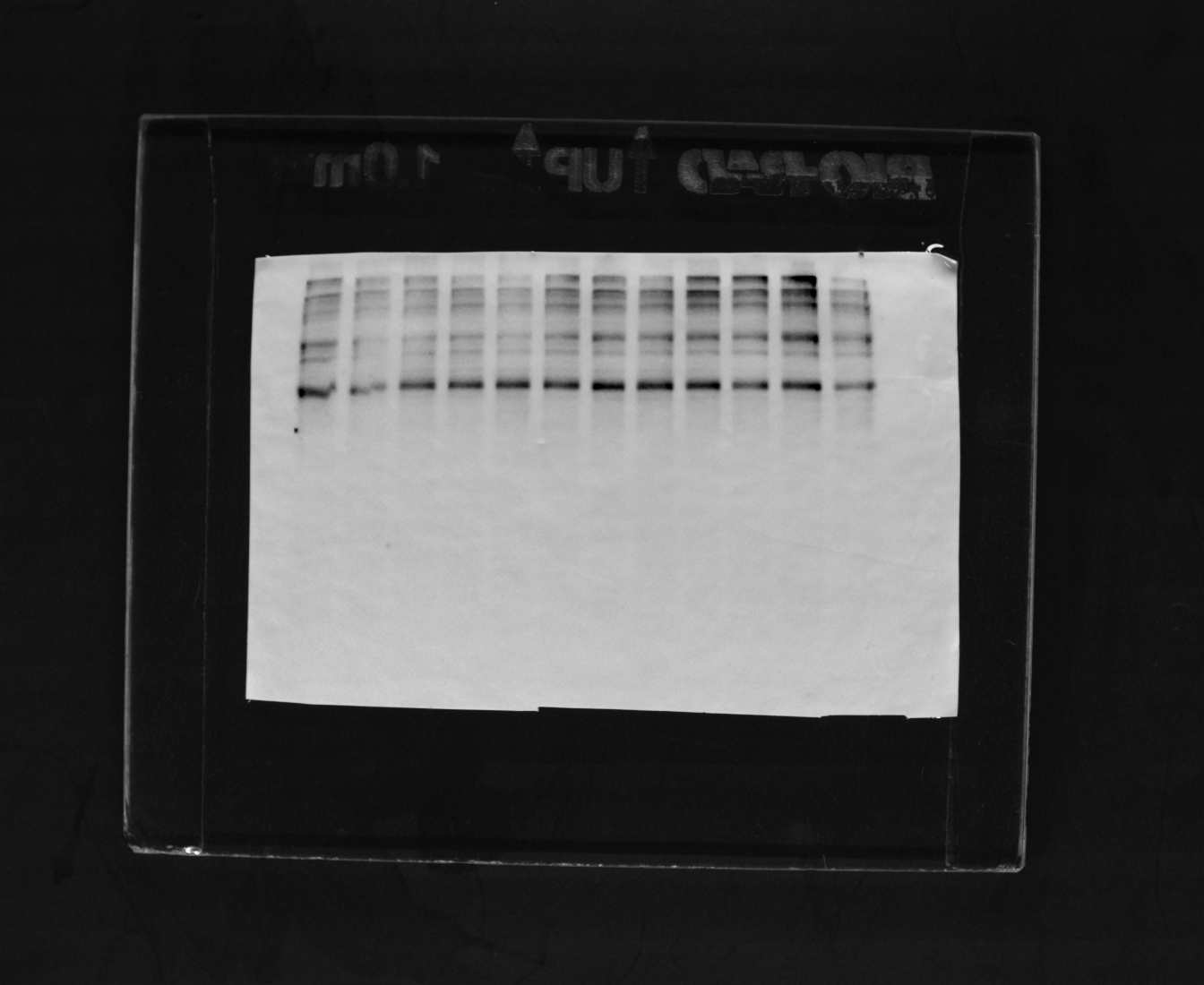

Supplement: Figure 6—figure supplement 1—source data 2. [file elife-108953-fig6-figsupp1-data2.zip › FigS6 Supplement data OGlNac.Tif]

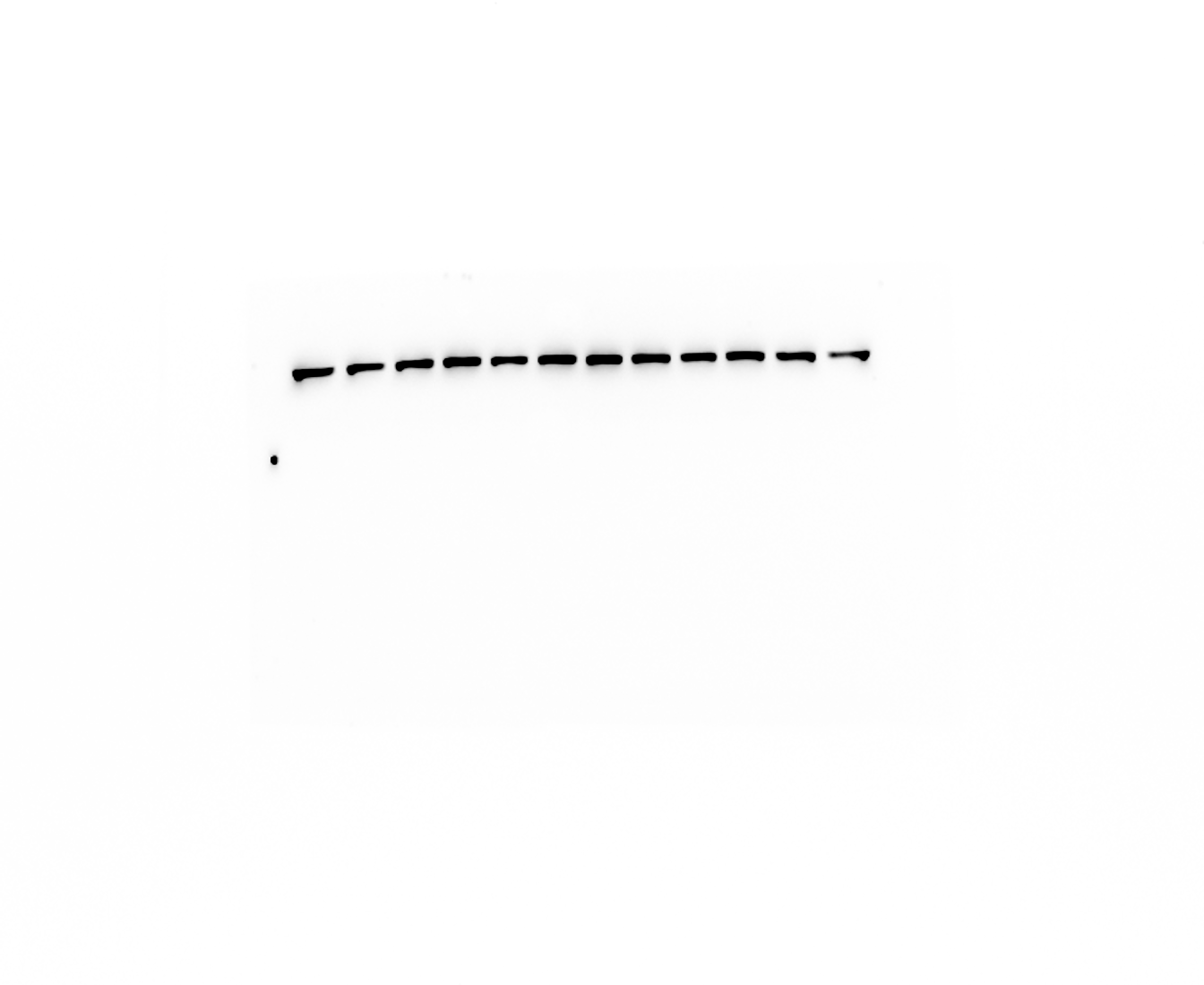

Supplement: Figure 6—figure supplement 1—source data 2. [file elife-108953-fig6-figsupp1-data2.zip › FigS6 Supplemental data HSP90.tif]

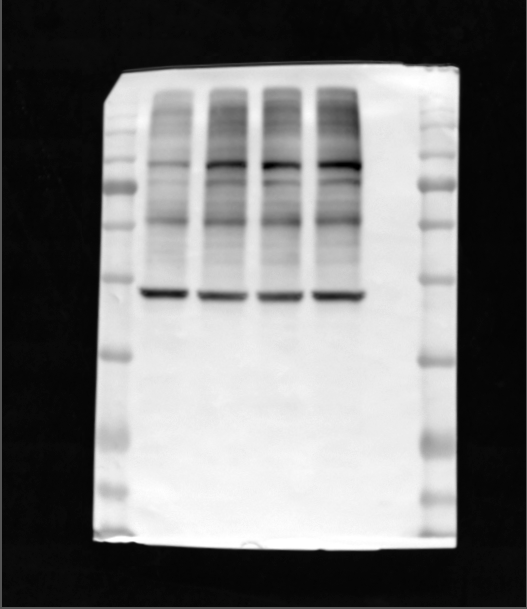

Supplement: Figure 7—source data 2. [file elife-108953-fig7-data2.zip › Input Actin .tif]

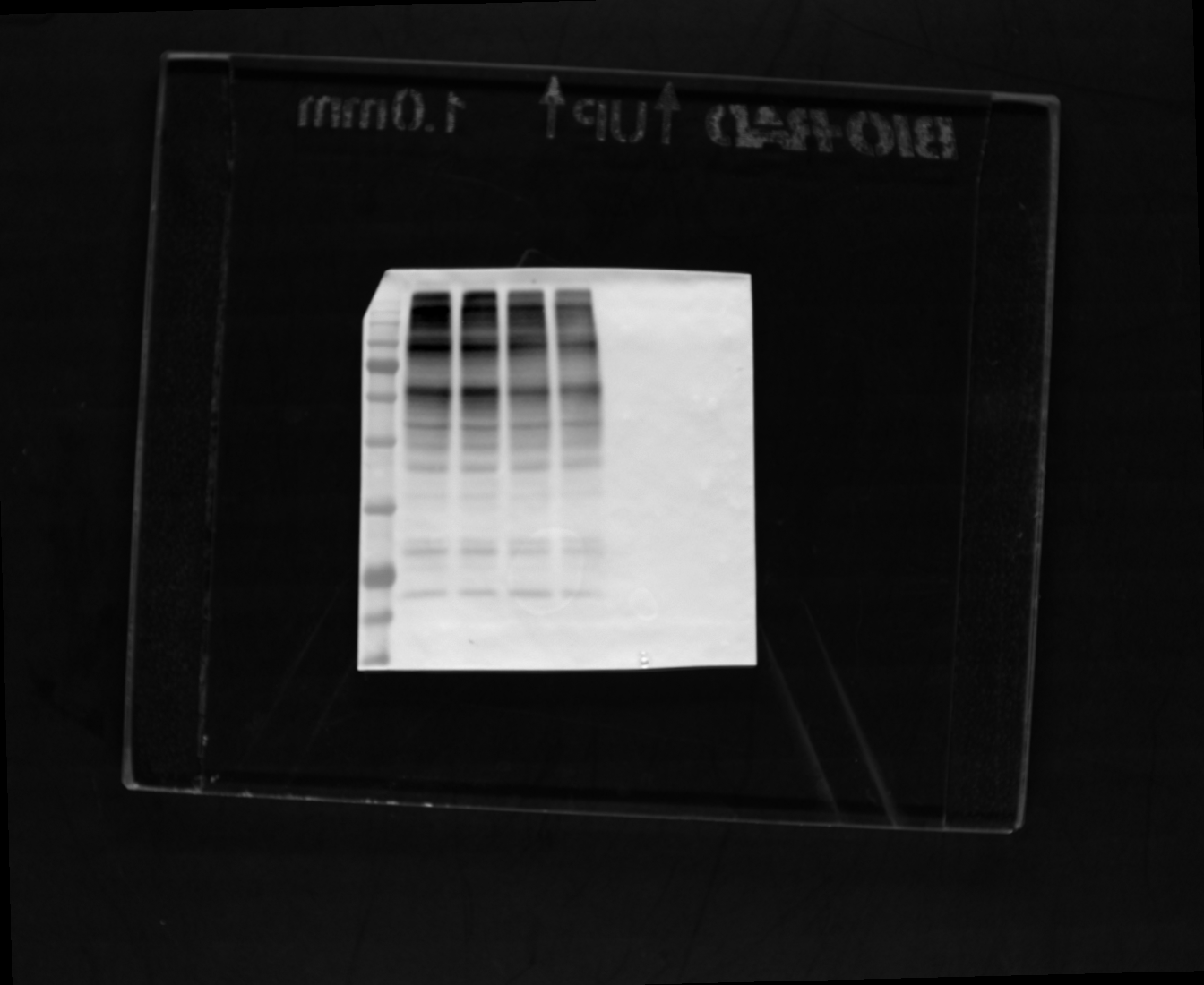

Supplement: Figure 7—source data 2. [file elife-108953-fig7-data2.zip › Input O-Glcnac.tif]

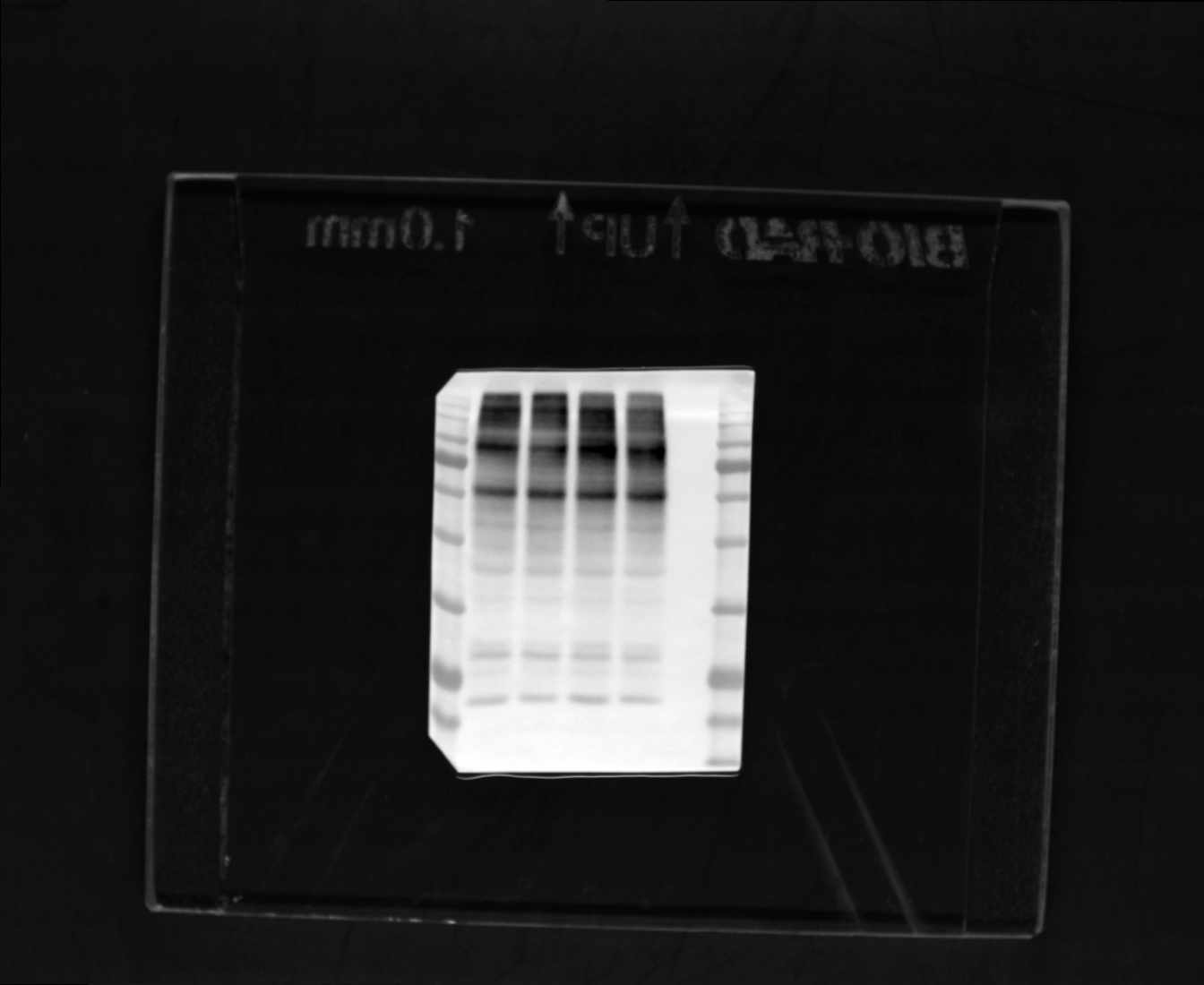

Supplement: Figure 7—source data 2. [file elife-108953-fig7-data2.zip › Input p65.tif]

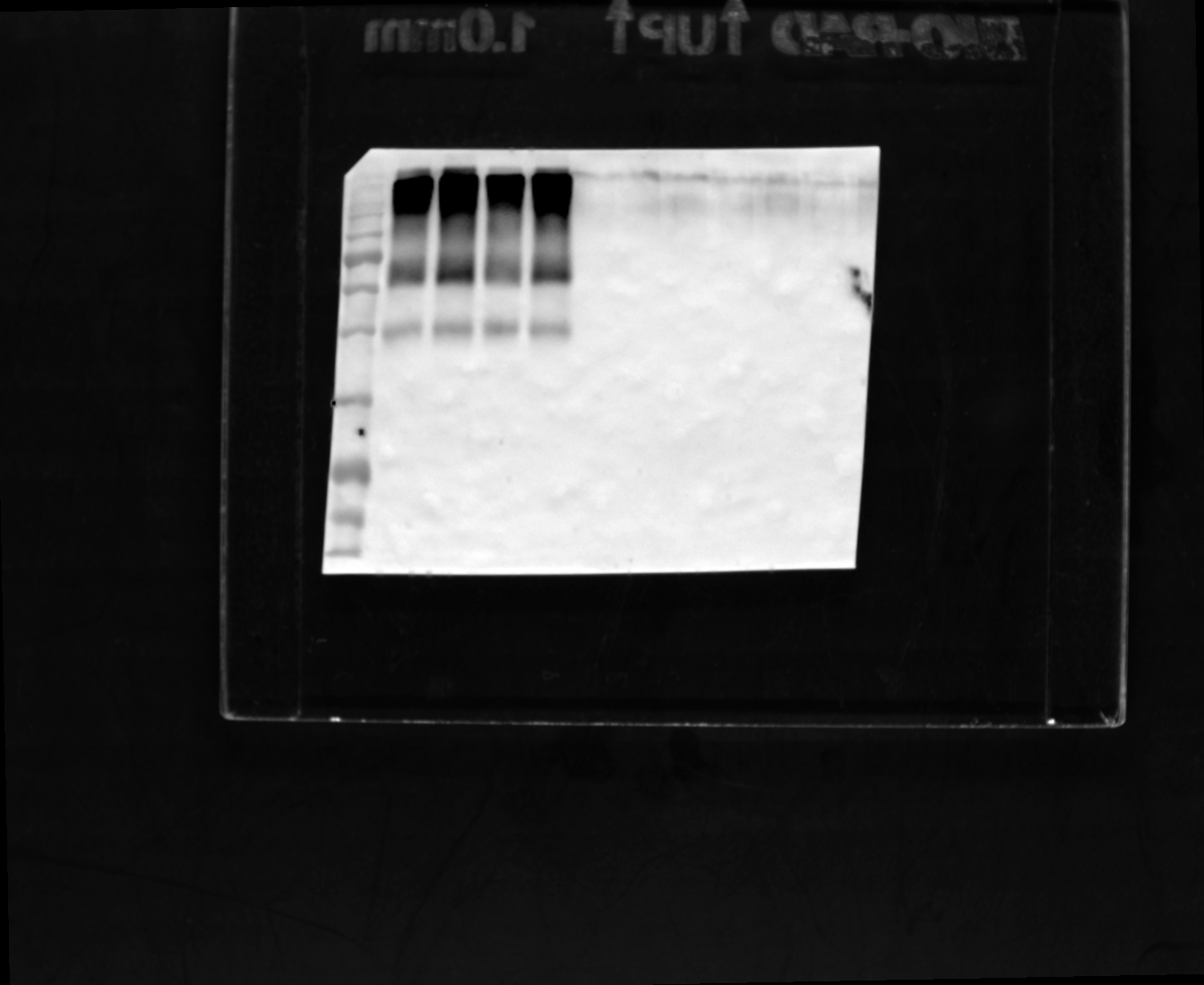

Supplement: Figure 7—source data 2. [file elife-108953-fig7-data2.zip › IP O-Glcnac.tif]

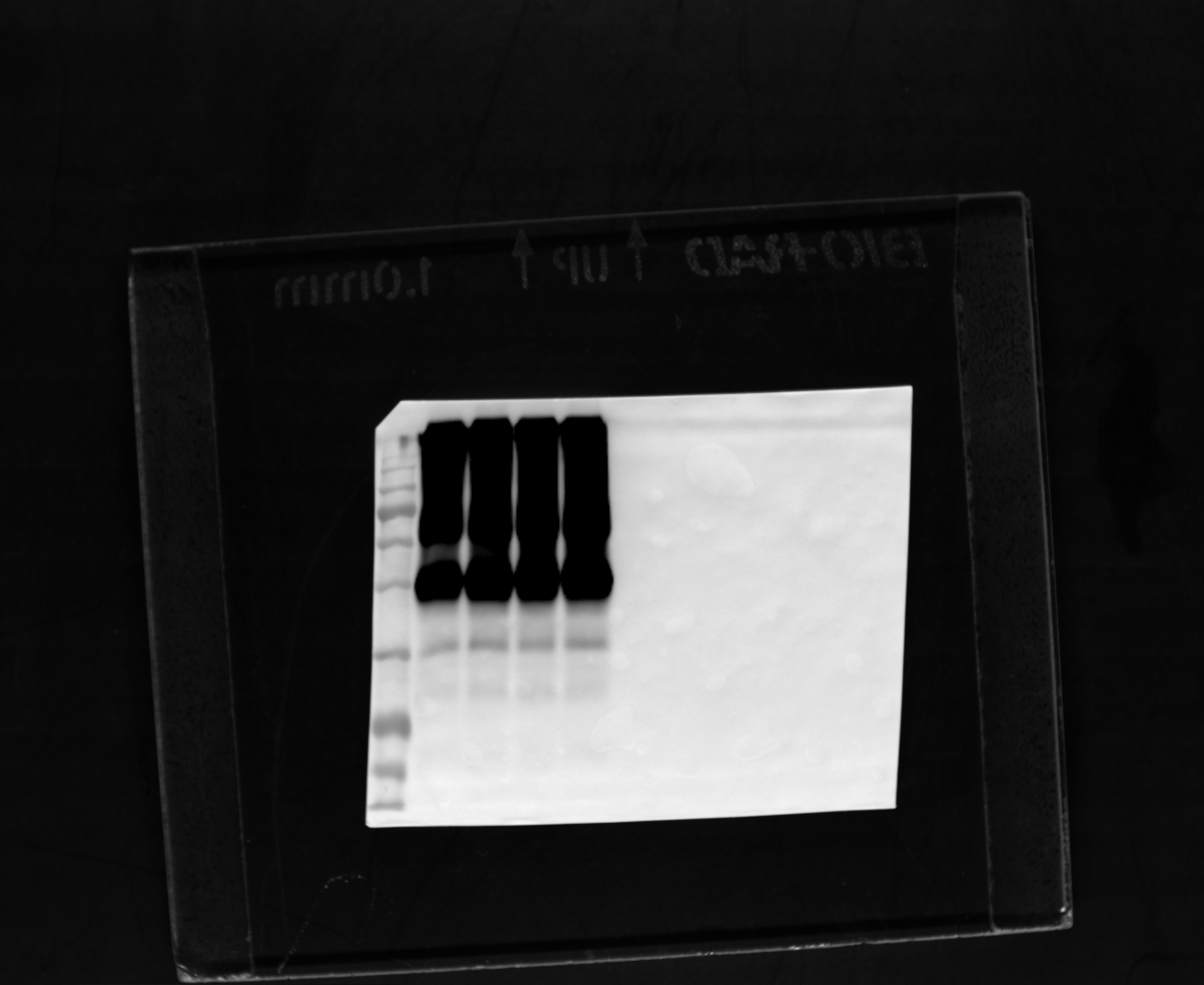

Supplement: Figure 7—source data 2. [file elife-108953-fig7-data2.zip › IP p65 Composite Molecular weight marker.Tif]

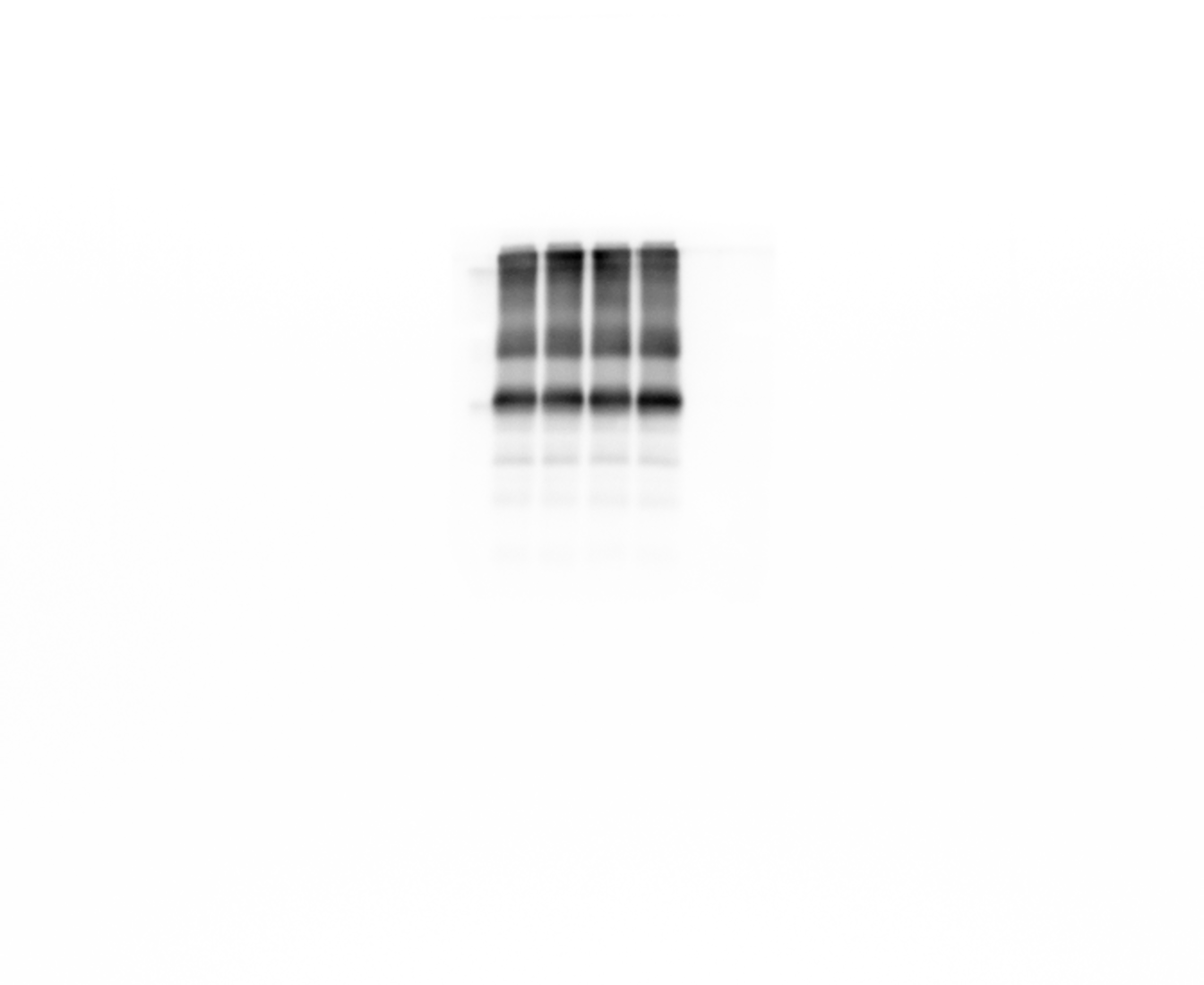

Supplement: Figure 7—source data 2. [file elife-108953-fig7-data2.zip › IP p65.tif]
