# Supplementary material for: Enteropathogenic Escherichia coli-mediated fast and coordinated Ca²+ responses regulate NF-κB activation
Source: eLife. 2026 Jul 22;14:RP108953. doi: 10.7554/eLife.108953 (PMC13391085; doi:10.7554/eLife.108953)
Supplement: Figure 7—source data 1. [file elife-108953-fig7-data1.zip › Figure 7A Source Data Input.pdf]

Fig 7A INPUT

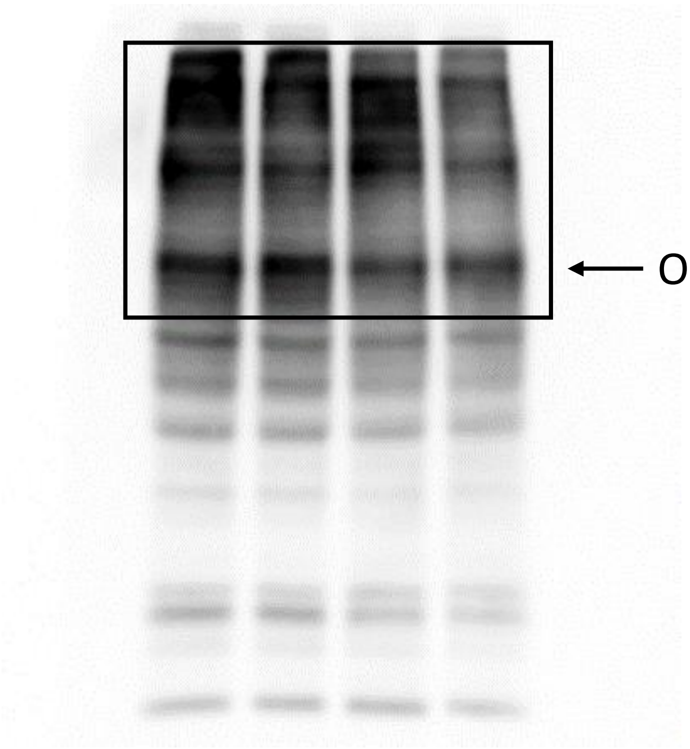

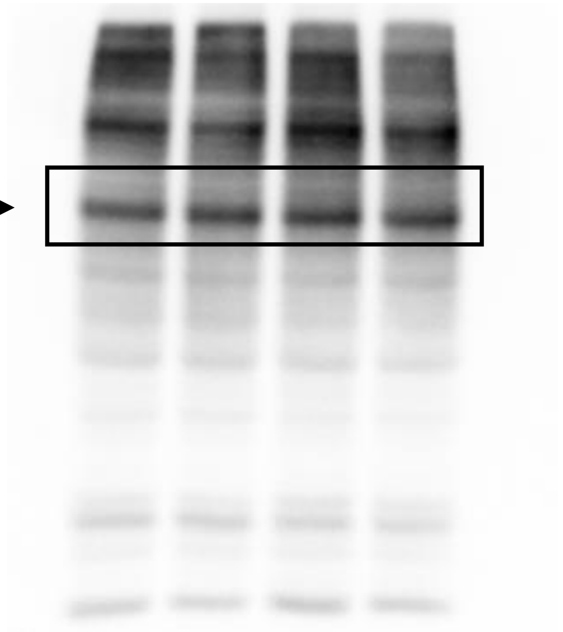

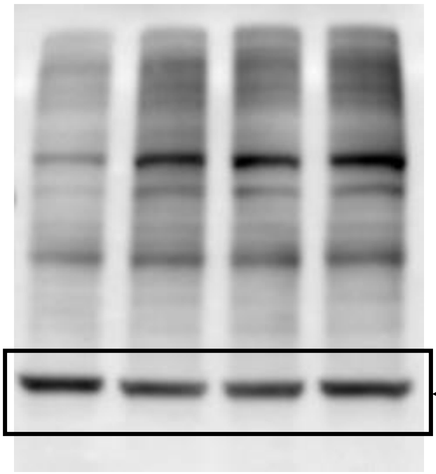

Figure 7, Source Data 1. Original membranes corresponding to the bottom of figure 7A. Specific lanes presented in the final manuscript are indicated with black box.
