# Supplementary material for: Enteropathogenic Escherichia coli-mediated fast and coordinated Ca²+ responses regulate NF-κB activation
Source: eLife. 2026 Jul 22;14:RP108953. doi: 10.7554/eLife.108953 (PMC13391085; doi:10.7554/eLife.108953)
Supplement: Peer review history

# Editor report: eLife Assessment

**DOI:** 10.7554/eLife.108953.3.sa0

**Authors:** Bree Aldridge

**Affiliations:** Tufts University School of Medicine United States

**Keywords:** Compelling, Important

This study reports **important** advances in our understanding of how enteropathogenic *E. coli* (EPEC) interacts at the intestinal interface. **Compelling** data describe a novel model of spatially coordinated calcium signaling to modulate NF-kB activation. These findings, which integrate imaging, genetics, and computational modeling, provide a new way to consider host-pathogen interactions in EPEC infections that may lead to improved therapies.

# Referee report: Reviewer #1 (Public review)

**DOI:** 10.7554/eLife.108953.3.sa1

Summary:

In their article, Guo and coworkers investigate the Ca²⁺ signaling responses induced by Enteropathogenic *Escherichia coli* (EPEC) in epithelial cells and how these responses regulate NF-κB activation. The authors show that EPEC induces rapid, spatially coordinated Ca²⁺ transients mediated by extracellular ATP released through the type III secretion system (T3SS). Using high-speed Ca²⁺ imaging and stochastic modeling, they propose that low ATP levels trigger "Coordinated Ca²⁺ Responses from IP₃R Clusters" (CCRICs) via fast Ca²⁺ diffusion and Ca²⁺-induced Ca²⁺ release. These responses may dampen TNF-α-induced NF-κB activation through Ca²⁺-dependent modulation of O-GlcNAcylation of p65. The interdisciplinary work suggests a new perspective on calcium-mediated immune response by combining quantitative imaging, bacterial genetics, and computational modeling.

Strengths:

The study provides a new concept for host responses to bacterial infections and introduces the concept of Coordinated Ca²⁺ Responses from IP₃R Clusters (CCRICs) as synchronized, whole-cell-scale Ca²⁺ transients with the fast kinetics typical of local events. This is elegantly done by an interdisciplinary approach using quantitative measurements and mechanistic modelling.

Comments on revised version.

The revised version of the manuscript has addressed all my raised points. I'd like to thank the authors for the work they have put into the revision to make this a very compelling publication.

# Referee report: Reviewer #2 (Public review)

**DOI:** 10.7554/eLife.108953.3.sa2

Summary:

The authors of this study are trying to resolve how cellular infection by enteropathogenic *E. coli* (EPEC) subverts cellular signaling pathways to promote infection and dampen immune responses. Specifically, alteration in calcium dynamics has been evidenced in the prior literature as a potential initiator of these adaptions, and this study provides ideas and mechanistic detail as to how cellular calcium dynamics may be subverted by pathogens.

Strengths:

The clear strengths of this paper relate to the new ideas inherent in the proposed hypothesis and their support from the experimental approaches used. Overall, the proposed work provides new ideas in this area, which will benefit from further investigation. Certainly, this is an interesting and challenging paradigm to pick apart mechanistically, and is important for improving treatments from intestinal infections. The authors have provided additional data to clarify and expand on concerns raised during the original review, and these additions are helpful.

Comments on revised version.

Thorough response to original review. No further comments.

# Author comment: Author response

**DOI:** 10.7554/eLife.108953.3.sa3

**Authors:** Fangrui GUO, Roberto Ornelas Guevara, Linda Oussaedine, Geneviève Dupont, Laurent COMBETTES, Guy Tran Van Nhieu

**Affiliations:** Institut de Biologie Intégrative de la Cellule Gif-sur-Yvette France; Universite Libre de Bruxelles (ULB) Brussels Belgium; Institut de Biologie Intégrative de la Cellule Gif-sur-Yvette France; Université Libre de Bruxelles Bruxelles Belgium; Institut de Biologie Intégrative de la Cellule Gif-sur-Yvette France; Institut de Biologie Intégrative de la Cellule Gif-sur-Yvette France

The following is the authors’ response to the original reviews.

**Public Reviews:**

**Reviewer #1 (Public review):**

Summary:

In their article, Guo and coworkers investigate the Ca²⁺ signaling responses induced by Enteropathogenic *Escherichia coli* (EPEC) in epithelial cells and how these responses regulate NF-κB activation. The authors show that EPEC induces rapid, spatially coordinated Ca²⁺ transients mediated by extracellular ATP released through the type III secretion system (T3SS). Using high-speed Ca²⁺ imaging and stochastic modeling, they propose that low ATP levels trigger "Coordinated Ca²⁺ Responses from IP₃R Clusters" (CCRICs) via fast Ca²⁺ diffusion and Ca²⁺-induced Ca²⁺ release. These responses may dampen TNF-α-induced NF-κB activation through Ca²⁺-dependent modulation of O-GlcNAcylation of p65. The interdisciplinary work suggests a new perspective on calcium-mediated immune response by combining quantitative imaging, bacterial genetics, and computational modeling.

Strengths:

The study provides a new concept for host responses to bacterial infections and introduces the concept of Coordinated Ca²⁺ Responses from IP₃R Clusters (CCRICs) as synchronized, whole-cell-scale Ca²⁺ transients with the fast kinetics typical of local events. This is elegantly done by an interdisciplinary approach using quantitative measurements and mechanistic modelling.

Weaknesses:

(1) The effect of coordination by fast diffusion for small eATP concentrations is explained by the resulting low Ca2+ concentration that is not as strongly affected by calcium buffers compared to higher concentrations. While I agree with this statement on the relative level, CICR is based on the resulting absolute concentration at neighboring IP3Rs (to activate them). Thus, I do not fully agree with the explanation, or at least would expect to use the modelling approach to demonstrate this effect. Simulations for different activation and buffer concentrations could strengthen this point and exclude potential inhibition of channels at higher stimulation levels.

We fully agree that CICR is determined by the local Ca^2+^ concentration at each IP_3_R cluster, not by a global cytosolic average. In our stochastic model, IP_3_ R clusters are represented as phenomenological entities at discrete spatial sites. Each cluster senses the local Ca^2+^ concentration at its position, and its stochastic gating depends only on this local [Ca^2+^] and on [IP3]. Buffers are not included explicitly. Instead, we use an effective Ca2+ diffusion coefficient Deff, which accounts for the effect of endogenous Ca^2+^ buffers. To reproduce the coordinated low-amplitude Ca^2+^ responses observed experimentally, we found that we had to use Deff = 100 µm^2^/s. In the supplementary analysis, we show that an effective diffusion coefficient of this order is indeed plausible for a realistic mixture of mobile and immobile Ca^2+^ buffers (Supplementary Note 2. Figure 1).

In the revised manuscript, we now provide a supplementary analysis (Supplementary Note 2) to justify this choice. Using an equation to compute the effective diffusion coefficient considering a plausible mixture of mobile and immobile buffers and an explicit reaction–diffusion model, we show that:

- The effective diffusion coefficient of Ca^2+^ becomes Ca^2+^ dependent, and

- There exists a regime in which low-amplitude Ca^2+^ elevations are characterized by an effective diffusion coefficient of Deff = 100 µm^2^/s and a larger spatial extent than higher-amplitude transients (Supplementary Note 2. Figure 1).

Thus, the value of Deff used in the cluster model is quantitatively consistent with classical buffering theory and with plausible cytosolic buffer mixtures. This provides a mechanistic basis for the observation that small-amplitude, short-lived events can nevertheless produce coordinated signals with large spatial extent and, occasionally, almost immediate activation of IP_3_R clusters at distant locations in both simulations and experiments.

In this respect, I would also include the details of the modelling, such as implementation environment, parameters, and benchmarking. The description in the Supplementary Methods is very similar to the description in the main text. In terms of reproducibility, it would be important to at least provide simulation parameters, and providing the code would align with the emerging standards for reproducible science.

We apologize for the lack of details of the modelling in the previous submission. In this revised version, we are providing with a full description of the model in the Supplementary Information, Note1.

To address the reviewer’s request for simulations at different activation levels, we now show an additional simulation in which [IP_3_] is higher (0.1 µM, constant in time and space) and Deff is set to 40 µm^2^/s (Supplementary Note 3). This lower effective diffusion coefficient is consistent with the stronger buffering and reduced Ca^2+^ mobility expected for higher-amplitude signals. In this case, the same phenomenological cluster model generates a global Ca^2+^ response with larger amplitude and longer duration, rather than a loss of activity due to excessive inhibition (Supplementary Note 3, Figure 1, left panel). The Supplementary Note 3. Figure 1, right panel shows the 2D cell geometry, where dots indicate the random positions of IP_3_R clusters whose behavior is described by our phenomenological cluster model.

(2) Quantitative characterization of CCRICs:

The paper would benefit from a clearer definition of the term CCRICs and quantitative descriptors like duration, amplitude distribution, frequency, and spatial extent (also in relation to the comment on the EGTA measurements below). Furthermore, it remains unclear to me whether CCRICs represent a population of rapidly propagating micro-waves or truly simultaneous events. Maybe kymographs or wave-front propagation analyses (at least from simulations if experimental resolution is too bad) would strengthen this point.

We agree and completed the description of the CCRICs by adding:

In the Results section, p. 8, l. 27:

“…with a duration of 2.1 ± 1.0 sec (mean ± SEM) (N = 4, 128 responses)”. p. 9, l. 13:

“In rare instances (less than 3%), typical local “Puff” responses elicited by these ATP concentrations could also be detected often occurring at the cell periphery (Figs. 4B, red region and 4C, red arrow; Fig. S6D, blue trace) (N > 20, cells > 500). As expected from the small concentrations of Ca^2+^ released at puff sites, no increase in cytosolic Ca^2+^ was detected in a distal cell region (Fig. S6D, top), indicating that isotropic Ca^2+^ diffusion from a puff release site cannot account for Ca^2+^ increase over large cell area. Puffs could also be detected concomitantly with CCRICs in different ROIs of the same cell (Fig. S6D, bottom). In contrast to puffs, CCRICs often showed responses of comparable amplitude in distal regions over the whole cell (Figs. 4C and S6A, B), suggesting the contribution from IP_3_R cluster activation by Ca^2+^-Induced Ca^2+^ Release (CICR). Within a given cell, the vast majority of CCRICs appeared quasi-synchronized at the fatest acquisition rate of 22 ms / frame that we could achieve. However, in few instances a delay could be detected in the elicitation of a peak in distant region of a cell (Fig. S6C). These observations suggest that the quasi-synchronization of CCRICs result from the fast diffusion of Ca^2+^ leading to the activation of IP_3_R clusters over large cell area, which may be delayed in a some instances. Scrutinizing of CCRICs showed that while their profiles were comparable, the amplitude of these responses varied in different regions of the cell, with often a single 1 µm^2^ region, likely corresponding the initial firing cluster, showing a prominent amplitude and other regions with smaller amplitude for a given response (Figs. 4B and 4C). For example, in Fig. 4C, the highest amplitude is observed in the red region for peaks 1 and 3, whereas it is observed and in the purple region for peak 2. Thus, for a given CCRIC, the respective contribution of local IP_3_R cluster activation and isotropic diffusion of Ca^2+^from other release sites in Ca^2+^ increase may vary in different regions of the cell”.

In the Discussion section, 2nd sentence p. 12:

“CCRICs showed rapid kinetics with an average duration of ca 2.1 seconds and amplitude corresponding to an increase in Ca^2+^ cytosolic concentration of a few hundreds nM, seemingly smaller than that of puffs (Fig. S6D), often occurring repeatedly with a frequency of up to 12 CCRICs / min over the whole cell.”

We have tried to clarify the notion of coordination versus synchronization of CCRICs by showing the delay observed in some instances in the elicitation of CCRICs at distal regions of the cell, now illustrated shown in Fig S6C.

(3) Specificity of pharmacological tools:

Suramin and U73122 are known to have off-target effects. Control experiments using alternative P2 receptor antagonists like PPADS or inactive U73343 analogs would strengthen the causal link.

As suggested by the referee, we have performed complementary experiments showing the inhibitory effects of PPADS and absence of effects of U73343 on EPEC-induced Ca2+ responses including CCRICs now shown in the amended Fig. S2.

**Reviewer #2 (Public review):**

Summary:

The authors of this study are trying to resolve how cellular infection by enteropathogenic *E. coli* (EPEC) subverts cellular signaling pathways to promote infection and dampen immune responses. Specifically, alteration in calcium dynamics has been evidenced in the prior literature as a potential initiator of these adaptations, and this study provides ideas and mechanistic detail as to how cellular calcium dynamics may be subverted by pathogens.

Strengths:

The clear strengths of this paper relate to the new ideas inherent in the proposed hypothesis and their support from the experimental approaches used. Overall, the proposed work provides new ideas in this area, which will benefit from further investigation. Certainly, this is an interesting and challenging paradigm to pick apart mechanistically, and is important for improving treatments from intestinal infections.

Weaknesses:

Additional insight is needed in three specific areas to convincingly support the conclusions drawn by the authors. These three areas are: first, a better description of the infection-associated calcium signals. Second, a mechanistic definition of the relevant purinoceptors versus other pathways to increase cellular calcium. Third, an effort to show that the proposed pathways have relevance in a polarized epithelial cell.

(1) first, a better description of the infection-associated calcium signals.

We agree and have added a more detailed description of the CCRICs in the results and discussion section, as detailed in response to referee 1, Weakness 2 by adding:

In the Results section, p. 8, l. 27:

“…with a duration of 2.1 ± 1.0 sec (mean ± SEM) (N = 4, 128 responses)”. p. 9, l. 13:

“In rare instances (less than 3%), typical local “Puff” responses elicited by these ATP concentrations could also be detected often occurring at the cell periphery (Figs. 4B, red region and 4C, red arrow; Fig. S6D, blue trace) (N > 20, cells > 500). As expected from the small concentrations of Ca^2+^ released at puff sites, no increase in cytosolic Ca^2+^ was detected in a distal cell region (Fig. S6D, top), indicating that isotropic Ca^2+^ diffusion from a puff release site cannot account for Ca^2+^ increase over large cell area. Puffs could also be detected concomitantly with CCRICs in different ROIs of the same cell (Fig. S6D, bottom). In contrast to puffs, CCRICs often showed responses of comparable amplitude in distal regions over the whole cell (Figs. 4C and S6A, B), suggesting the contribution from IP_3_R cluster activation by Ca^2+^-Induced Ca^2+^ Release (CICR). Within a given cell, the vast majority of CCRICs appeared quasi-synchronized at the fatest acquisition rate of 22 ms / frame that we could achieve. However, in few instances a delay could be detected in the elicitation of a peak in distant region of a cell (Fig. S6C). These observations suggest that the quasi-synchronization of CCRICs result from the fast diffusion of Ca^2+^ leading to the activation of IP_3_R clusters over large cell area, which may be delayed in a some instances. Scrutinizing of CCRICs showed that while their profiles were comparable, the amplitude of these responses varied in different regions of the cell, with often a single 1 µm^2^ region, likely corresponding the initial firing cluster, showing a prominent amplitude and other regions with smaller amplitude for a given response (Figs. 4B and 4C). For example, in Fig. 4C, the highest amplitude is observed in the red region for peaks 1 and 3, whereas it is observed and in the purple region for peak 2. Thus, for a given CCRIC, the respective contribution of local IP_3_R cluster activation and isotropic diffusion of Ca^2+^ from other release sites in Ca^2+^ increase may vary in different regions of the cell” In the Discussion section, 2nd sentence p. 12:

“CCRICs showed rapid kinetics with an average duration of ca 2.1 seconds and amplitude corresponding to an increase in Ca^2+^ cytosolic concentration of a few hundreds nM, seemingly smaller than that of puffs (Fig. S6D), often occurring repeatedly with a frequency of up to 12 CCRICs / min over the whole cell.”

We have tried to clarify the notion of coordination versus synchronization of CCRICs by showing the delay observed in some instances in the elicitation of CCRICs at distal regions of the cell, now illustrated shown in Fig S6C.

CRICCs are observed over the whole cell or very large cell area. We agree that this point as well as comparison with previously described puffs needed clarification. We have added the following sentences in the discussion and inserted the seminal Thomas et al. 1999 citation in the references, p. 13, l. 18:

“Consistently, while CRICCs were detected in the vast majority of cells at these very low agonist concentrations, in rare instances, local “puff-like” responses were also detected at the cell periphery. These observations are in contrast to previously described Ca^2+^ puffs preceding global responses reported to occur preferentially in perinuclear area (Thomas et aL., 1999). These earlier studies, however, involved higher agonist concentrations (1-5 µM ATP) expected to lead to the release of higher IP_3_ concentrations, which may preferentially stimulate larger IP_3_R clusters at the perinuclear region because of the higher density of IP_3_ Rs. In addition, larger IP_3_ clusters may release higher amounts of Ca^2+^ for which, as opposed to CCRICs, diffusion would be restrained by Ca^2+^ buffers thereby favoring the spatial confinement of the response. “

(2) Second, a mechanistic definition of the relevant purinoceptors versus other pathways to increase cellular calcium

We do not believe that CCRICs are specific to EPEC, since they are also elicited by low agonist concentrations. The discrete action of Type III translocons leading to the release of small amounts of extracellular ATP at the onset of EPEC prompted us to perform fast Ca^2+^ imaging at low agonists concentrations (150 nM ATP, 100 nM histamine now shown in Fig. S4), which to our knowledge, differ from higher agonist concentrations used in all previous studies describing puffs. Our modelling studies support the notion that CCRICs correspond to generic Ca^2+^ release-dependent responses triggered by low levels of IP3.

We now show inhibition of CCRICs by PPADS, another purinergic receptor antagonist, and extracellular ATP depletion by addition of hexokinase in the extracellular medium in Figs. S4 and S7.

Knocking down ATP receptors represents a challenging task since HeLa cells were shown to express transcripts for most of the described 8 P2Xs and 7 P2Ys purinergic receptors (10.1016/j.bbamem.2009.03.006). Mostly, we do not believe that CCRICs are triggered by a specific ATP receptor and do not expect to see inhibition of CCRICs in single knock-down experiments. Our experimental and modelling studies suggest that CCRICs are not specific to EPEC nor to a particular ATP receptor, but instead correspond instead to generic Ca^2+^ elicited at low agonist concentrations such as ATP or histamine.

Zhong et al., 2020 indeed previously showed a role for Ca^2+^ influx mediated by the TRPV2 receptor in EPEC-mediated cell death. However, this influx occurred following 8 hours of cell infection with EPEC. We do not detect significant cell death or Ca^2+^ influx at the onset of infection corresponding to the 12 hours infection kinetics that we used. Our experiments indicate that CCRICs do not involve Ca^2+^ influx.

(3) Third, an effort to show that the proposed pathways have relevance in a polarized epithelial cell.

We agree and have performed complementary experiments showing induction of CCRICs by EPEC and eATP in polarized intestinal epithelial cells, now shown in Figure S8.

**Recommendations for the authors:**

**Reviewer #1 (Recommendations for the authors):**

(1) Statistical treatment and data presentation:

Some figure legends lack clarity on replicates (n = cells vs N = independent experiments). Timecourse quantifications of p-IκB and p-p65 should include normalized fold-change plots with clear statistical tests.

To clarify, we replaced “n” by “cells”. The number of determinations and independent experiments (N) has been added in the legends to all relevant Figures and Supplementary Figures.

As requested, we now show the p-IκB and p-p65 plots as plots normalized to basal p-IκB and p-p65 levels. We mentioned in legend to Fig. 6 that we used an ANCOVA test showing significance of the effects of eATP on TNF-∝-induced IκB- and p65 phosphorylation.

(2) Clarification on the temperature used in imaging (why measured at 35{degree sign} C)?

We have added the following clarification in the Materials and Methods section p. 14, l. 21:

“Imaging was then carried out at 35°C to allow for bacterial type III secretion, …”

(3) Figure 4A:

The image shows a lower image acquisition interval than every 2s that is stated in the caption.

We apologize for the mistake. The legend to Fig. 4A now reads:

“Image acquisition every 52 ms (A)…”

(4) Figure 4B:

The color of ROIs could be more intense for better identification.

We have replaced the colors of blue and green ROIs, by light cyan and purple ROIs

(5) Figure 4c:

I don't understand the meaning of the dashed lines described by "The dashed red and green lines point at the aggregation of responses throughout the cell" in the caption or in the text.

We apologize for the lack of clarity and have re-written the corresponding text p. 9, l.25 as follows:

“Scrutinization of CCRICs showed that while their profiles were comparable, the amplitude of these responses varied in different regions of the cell, with often a ca 3 µm^2^ single region, likely corresponding to a source point release, showing a prominent amplitude and other regions with smaller amplitude for a given response (Figs. 4B and 4C). For example, in Fig. 4C, the highest amplitude is observed in the red region for peaks 1 and 3, whereas it is observed and in the purple region for peak 2. Thus, for a given CCRIC, the respective contribution of local IP3R cluster activation and isotropic diffusion of Ca^2+^ from other release sites in Ca^2+^ increase may vary in different regions of the cell.”

(6) Figure S4A:

The responses for EGTA are not really pointed out. Are the traces meant to show events?

We have added arrowheads in traces corresponding to ATP + EGTA-AM treatment pointing at “flattened Ca^2+^ responses”. The Legend to Fig. S4A now includes the sentence: “ATP + EGTA-AM treatment led to an inhibition of Ca^2+^ responses, associated with small variations in the Ca^2+^ baseline, that were arbitrarily scored as flattened Ca^2+^ pseudo-responses (ATP+EGTA-AM, red arrows).”

(7) Figure S5:

Could not identify the purple arrow for the less mobile cluster.

We agree that the former Figure lacked clarity and have remade Figure S5, now Figure S6, with higher magnification of panels with fast acquisition. The previously purple arrows pointing at larger and less mobile clusters are now shown in black in these enlarged panels. The legend has been changed accordingly.

(8) There are some typos and suboptimal formulations throughout the manuscript, such as:

P8: "minute amount" could be changed to low, minor or similar.

“minute” amounts of eATP was replaced by “low amounts of eATP”.

P8: put a "%" to the numbers 61.2 {plus minus} 5.8.

“%” was added.

P16: "manuscript".

Thank you.

**Reviewer #2 (Recommendations for the authors):**

Suggestions relate to the following three topics.

First, a better description of the infection-associated calcium signals. The authors emphasize throughout the paper that their imaging data challenge established concepts in the calcium signaling field (discussion). I do not see the calcium imaging data explained either with data or textually with sufficient clarity to evaluate this assertion. A start would be a clear description of the characteristics of the EPEC-evoked calcium signals relative to other local and global domains of calcium signaling previously described in HeLa cells. Prior work has shown that PI-coupled agonists evoke local calcium signals that are perinuclear in HeLa cells (PMID: 10660296), but the relationship of EPEC-evoked transients to these previously defined responses is not clear.

We agree and have added a more detailed description of the CCRICs in the results and discussion section, as detailed in response to referee 1, Weakness 2.

Most importantly, it is ambiguous where in the HeLa cell recordings are made. Are these recordings close to the plasma membrane and/or deeper within the cell? The only spatial information is provided in Figure 3A, and these responses are not well described in the text or presented in a way that comparisons can be made to responses from a PI-coupled agonist.

CRICCs are observed over the whole cell or very large cell area. We agree that this point as well as comparison with previously described puffs needed clarification. We have added the following sentences in the discussion and inserted the seminal Thomas et al. 1999 citation in the references, p. 13, l. 18:

“Consistently, while CRICCs were detected in the vast majority of cells at these very low agonist concentrations, in rare instances, local “puff-like” responses were also detected at the cell periphery. These observations are in contrast to previously described Ca^2+^ puffs preceding global responses reported to occur preferentially in perinuclear area (Thomas et aL., 1999). These earlier studies, however, involved higher agonist concentrations (1-5 µM ATP) expected to lead to the release of higher IP_3_ concentrations, which may preferentially stimulate larger IP_3_R clusters at the perinuclear region because of the higher density of IP_3_Rs. In addition, larger IP_3_ clusters may release higher amounts of Ca^2+^ for which, as opposed to CCRICs, diffusion would be restrained by Ca^2+^ buffers thereby favoring the spatial confinement of the response. “

If I understand the described responses correctly, could not these rapid local responses result from a change in cellular calcium buffering capacity consequent to infection? Are the authors proposing that these responses occur in other cells also, or represent a pathogen-specific signaling mode?

We do not believe that CCRICs are specific to EPEC, since they are also elicited by low agonist concentrations. The discrete action of Type III translocons leading to the release of small amounts of extracellular ATP at the onset of EPEC prompted us to perform fast Ca^2+^ imaging at low agonists concentrations (150 nM ATP, 100 nM histamine now shown in Fig. S4), which to our knowledge, differ from higher agonist concentrations used in all previous studies describing puffs. Our modelling studies support the notion that CCRICs correspond to generic Ca^2+^ release-dependent responses triggered by low levels of IP3.

Second, evidence supporting a mechanistic role of ATP comes from prior literature, together with the authors' presented data showing the effects of PLC (to inhibit IP3), pharmacological inhibition (suramin, a non-selective purinoceptor blocker), and the effects of T3SS-deficient mutants (to prevent ATP release). However, there are missing steps here to mechanistically identify how ATP is working. First, does degradation of extracellular ATP (e.g., apyrase) block these responses? Second, given HeLa cells are easily amenable to knockdown approaches, does knockdown of particular ATP receptors, or TRPV2 as suggested in the prior literature, impact the calcium signal dynamics?

We now show inhibition of CCRICs by PPADS, another purinergic receptor antagonist, and extracellular ATP depletion by addition of hexokinase in the extracellular medium in Figs. S4 and S7.

Knocking down ATP receptors represents a challenging task since HeLa cells were shown to express transcripts for most of the described 8 P2Xs and 7 P2Ys purinergic receptors (10.1016/j.bbamem.2009.03.006). Mostly, we do not believe that CCRICs are triggered by a specific ATP receptor and do not expect to see inhibition of CCRICs in single knock-down experiments. Our experimental and modelling studies suggest that CCRICs are not specific to EPEC nor to a particular ATP receptor, but instead correspond instead to generic Ca^2+^ elicited at low agonist concentrations such as ATP or histamine.

Zhong et al., 2020 indeed previously showed a role for Ca^2+^ influx mediated by the TRPV2 receptor in EPEC-mediated cell death. However, this influx occurred following 8 hours of cell infection with EPEC.

We do not detect significant cell death or Ca^2+^ influx at the onset of infection corresponding to the 12 hours infection kinetics that we used. Our experiments indicate that CCRICs do not involve Ca^2+^ influx.

Third, while the use of HeLa cells provides advantages for imaging and mechanistic assays, the effort to replicate findings in an intestinal cell line would heighten relevance, given the likely importance of cell type and cell polarity on the pathogen-evoked responses.

We agree and have performed complementary experiments showing induction of CCRICs by EPEC and eATP in polarized intestinal epithelial cells, now shown in Figure S8.
